# Recipes of Ancient Egyptian kohls more diverse than previously thought

**DOI:** 10.1038/s41598-022-08669-0

**Published:** 2022-04-08

**Authors:** Marabel Riesmeier, Jennifer Keute, Margaret-Ashley Veall, Daniel Borschneck, Alice Stevenson, Anna Garnett, Alice Williams, Maria Ragan, Thibaut Devièse

**Affiliations:** 1 Research Laboratory for Archaeology and the History of Art, University of Oxford, Oxford, OX1 3TG UK; 2 CEREGE, Aix-Marseille University, CNRS, IRD, INRAE, Collège de France, Technopôle de l’Arbois, 13545 Aix-en-Provence, France; 3 University College London, Petrie Museum, London, WC1E 6BT UK; 4 Department of History and Philosophy of Science, University of Cambridge, Cambridge, CB2 3RH UK; 5 Canadian Conservation Institute, Ottawa, ON K1B 4S7 Canada; 6 UCL Institute of Archaeology, University College London, London, WC1E 6BT UK; 7 St Barbe Museum and Art Gallery, Lymington, SO41 9BH UK

**Keywords:** Infrared spectroscopy, Mass spectrometry, X-ray diffraction

## Abstract

Kohl, a dark eye cosmetic, is a well-known part of Ancient Egyptian culture. Modern chemical analyses of kohls have largely found lead-based inorganic constituents, whereas earlier studies argued for a much broader range of constituents. Furthermore, organic materials in kohls remain severely understudied. This raises questions regarding the true diversity of materials and recipes used to produce kohls. We analysed the contents of 11 kohl containers from the Petrie Museum collection in London. The objects selected cover a broad range of times and locations in Egypt. Our multi-analytical approach allowed us to characterise both inorganic and organic components. Our data show that inorganic ingredients in kohl recipes are not only lead-based but also manganese- and silicon-based. Our analyses also revealed that organic ingredients derived from both plant and animal sources were commonly used in kohl recipes and sometimes even represent the main constituent. All these findings point towards more varied recipes than initially thought and significantly shift our understanding of Ancient Egyptian kohls.

## Introduction

Kohl is a dark eye cosmetic, popular in Ancient Egypt and many other cultures throughout the ages. It has also been used by Romans, Chinese, Japanese, Phoenicians, Indians and Muslims^1^. Kohl has been used since at least 5000 BC and continues to be used today^2^. The word ‘kohl’ is of Arabic origin^1^ and has been used to define drugs and cosmetics of various compositions applied to the eyes^3^. It generally refers to dark eye cosmetics in the form of a fine powder containing one or more ingredients^1^ as well as dried out cakes, sticks^4^, stones^5^ and pastes^6^ made from such powders. It was and is still used on lashes and brows and to draw a line around the perimeter of the eye^7^. Make-Up was used in Ancient Egypt not only for aesthetic reasons but also for hygienic, therapeutic and religious functions^8^. The traditional black or sometimes green kohl was part of sacred rites and also served medicinal purposes^9^. The archaeological record shows that green eye paints were more common than black ones in predynastic times, but around the start of the (proto)dynastic period, black became more common and largely replaced green^10^. There are, however, some uncertainties regarding the variety of organic and inorganic ingredients that have been used in kohl recipes. Lead-based materials make up the main components in 85 out of 87 samples analysed with any modern methods before this study^10–16^ (discounting repeat analyses of the same samples, see Supplementary Table S1 for a comprehensive list). However, the components identified in earlier analyses of 74 kohls, summarized by Lucas and Harris^17^, are considerably more diverse. The discrepancy raises the question of whether the diversity of inorganic materials used in kohls may be greater than captured by modern analyses thus far.

Although there has been a considerable amount of research on the use of inorganic substances, especially lead-based ones, in kohl recipes from Ancient Egypt, the organic materials possibly present in kohls have received much less attention. Analyses performed pre-1930 on kohls revealed the presence of wax, fatty matter, balms and resins in very few specimens^17^. Lucas and Harris, however, doubt whether these specimens were eye-paints or other medicinal preparations. They speculate that kohl paste was prepared with water or water-soluble gum^17^. To our knowledge, only one modern study reports the presence of organic materials in kohls ^11^. Significant amounts of fatty acids were identified via gas chromatography/mass spectrometry (GC/MS) in four specimens of kohl. The use of organic materials in kohls thus remains understudied and debated.

**Figure 1:**
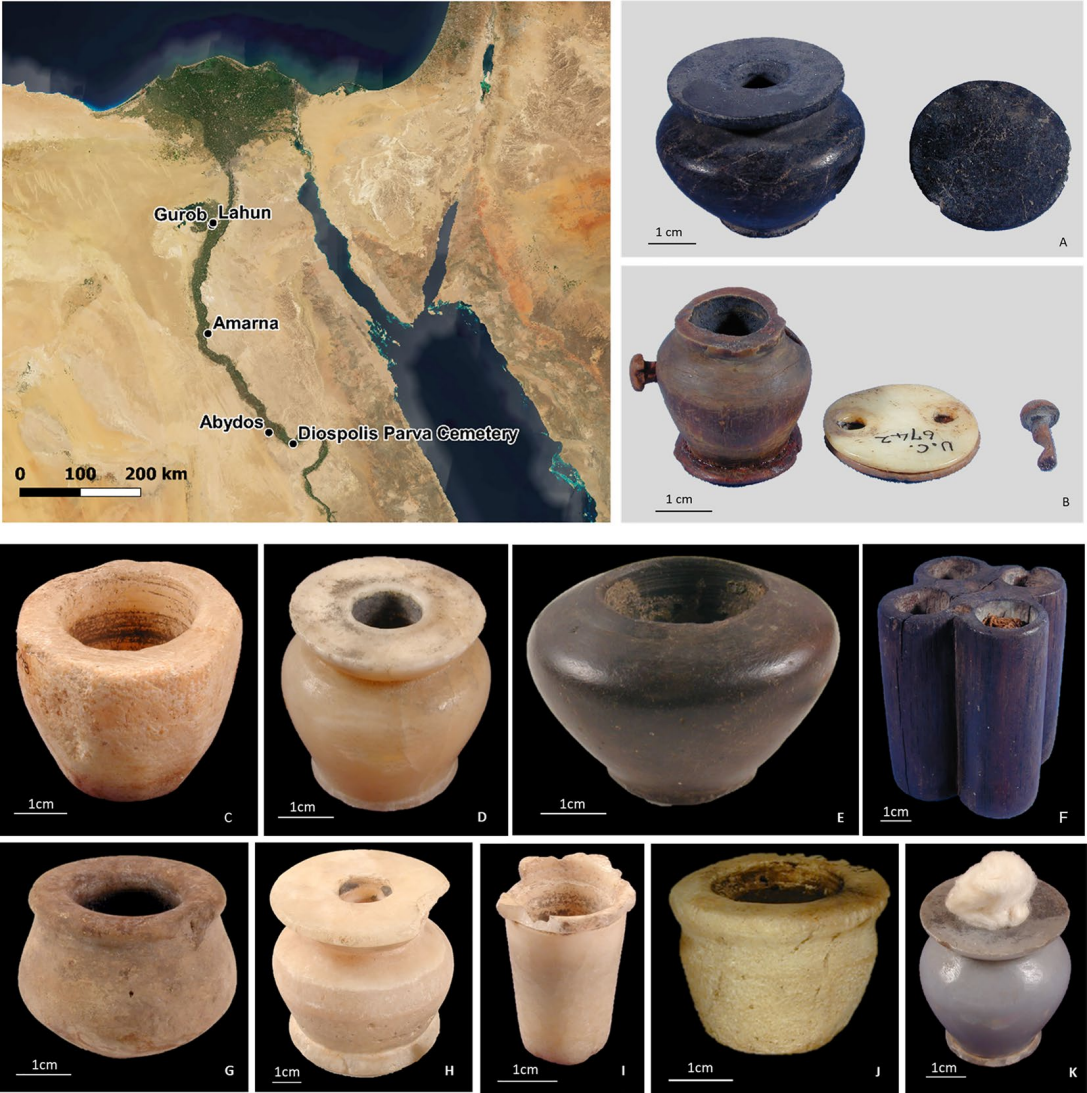
Map and pictures of the 11 kohl containers from the Petrie Museum. Objects UC7321 (**A**) and UC6742 (**B**) are from Lahun; objects UC43078 (**C**), UC42810 (**D**), UC43107 (**G**), UC43148 (**H**) and UC43159 (**I**) are from Abydos; object UC46343 (**E**) is from Amarna; object UC7890 (**F**) is from Gurob; object UC31613 (**J**) is from Diospolis Parva Cemetery; and object UC64751 (**K**) is of unknown provenance. Full description of each object is provided in the Supplementary Table S15. The map was created using QGIS (QGIS.org, 2022. QGIS Geographic Information System. QGIS Association).

The purpose of our study was to investigate the variety of both organic and inorganic ingredients through the study of twelve different kohls from eleven kohl containers from the Old Kingdom to the New Kingdom kept in the collection of the Petrie Museum in London (Fig. 1).

## Results

The samples were first screened using Fourier Transform Infrared Spectroscopy (FTIR). Inorganic components were then characterised by Scanning Electron Microscopy/Energy Dispersive X-Ray Spectroscopy (SEM/EDS) and Powder X-Ray Diffractometry (PXRD) while organic ones were identified by Gas Chromatography/Mass Spectrometry (GC/MS). Results are presented following this analytical strategy.

### FTIR screening

Samples can be divided into three main groups based on the results of the FTIR analyses: (1) inorganic dominant, (2) mixed organic and inorganic, and (3) unknowns. The detailed FTIR interpretation for each specimen analysed can be found in the Supplementary Information Appendix 2 (Supplementary Figs. S1–S57 and Supplementary Tables S2–S12). Four specimens produced FTIR spectra corresponding to inorganic materials. Quartz was identified in UC31613 (Supplementary Fig. S3), while peaks from UC43159 (Supplementary Fig. S8) are indicative of the presence of gypsum^18,19^. The FTIR profile for UC7321 (Supplementary Fig. S38) is attributed to cerussite (PbCO_3_) and consistent with known references^20–22^. Peaks from UC7890b (Supplementary Fig. S52) indicate the presence of atacamite (Cu_2_Cl(OH)_3_)^20,23^. Weak signals associated with C–H stretches of aliphatics^24^ were identified for the samples from the objects UC43148, UC46348, UC6742, UC7890a and UC43107 (Supplementary Figs. S23, S33, S43, S48, and S56, respectively). Spectra from UC7890a and UC46348 are more complex than those of the other specimens from 1650 cm^−1^ onwards, yielding peaks that may be assigned to amides, COO– anions, C–C=C ring structures, C–H, C–O, C–O–C, and –OH functional groups attributed to either aliphatics, aromatics, phenols, or alcohols^24^. The FTIR spectra of the specimens UC42810, UC43078 and UC64751 (Supplementary Figs. S13, S18 and S28, respectively) do not exhibit peaks that could be attributed to any particular organic or inorganic source. All three samples also exhibited a left baseline drift, which may be accounted for by the presence of material with a rough surface or inorganic components such as elemental manganese, lead, and iron^25^. Based on these preliminary findings, it was decided to analyse inorganic constituents by SEM/EDS and PXRD and organics using GC/MS.

### Identification of inorganic ingredients

Results are presented in three sections, discussing first the major and then the minor and trace components identified by SEM/EDS analyses and in conjunction with the associated PXRD data (Table 1). These groups are qualitative assessments of the elemental composition based on relative abundances^26^. The assigned elements are based on the characterized peaks in the EDS spectra.

**Table 1:** Results of qualitative SEM/EDS analysis and PXRD analyses. The terms major, minor, and trace components are based on relative abundances found in SEM/EDS analyses. Major is defined as more than 10 atomic %; minor as 1–10 atomic %; and, trace as less than 1 atomic %^26^.

Samples	Elemental composition (atomic %)			PXRD results
	Major	Minor	Trace	
UC43159	O (65.27) C (28.71)	Pb (1.68)	Al (0.35) Si (0.61) P (0.82) S (0.26) Cl (0.66) K (0.08) Ca (0.51) Mn (0.78) Fe (0.14) Cu (0.13)	Anglesite, Laurionite, Cerussite, Gypsum, Cotunnite, Calcite, Manganite
UC42810	O (65.35) C (28.92)	Si (1.28) Ca (1.19)	Pb (0.93) Al (0.53) Cl (0.38) Mg (0.35) Fe (0.34) P (0.24) S (0.18) Na (0.13) Mn (0.10) K (0.07)	Galena, Calcite, Cerussite, Quartz, Laurionite, Paralaurionite
UC43078	O (56.37) Mn (30.14) C (10.02)		Si (0.93) S (0.84) Ca (0.74) Al (0.33) Na (0.20) K (0.16) P (0.08)	Manganite, Gypsum, Graphite, Calcite
UC43148	O (60.27) C (18.56) Mn (16.51)	Si (1.15)	Al (0.76) Na (0.70) Ca (0.42) S (0.40) Pb (0.29) Cl (0.28) P (0.23) Mg (0.21) Cu (0.12) K (0.11)	Manganite, Calcite, Litharge, Galena, Quartz
UC64751	Pb (38.77) S (38.16) C (11.94)	O (6.61) Cl (4.52)		Cerussite, Laurionite, Hematite, Galena
UC46348	O (66.24) C (31.29)		Si (0.78) Mg (0.45) Al (0.36) Na (0.25) S (0.2) Ca (0.14) Fe (0.13) K (0.05) Cl (0.05) Zn (0.03) P (0.02)	Calcite, Lizardite, Gypsum, Quartz, Talc
UC7321	C (43.08) O (24.68) Pb (15.71) S (13.15)	Cl (1.35) Zn (1.04)	Fe (0.56) Ca (0.42)	Cerussite, Galena, Phosgenite, Litharge, Smithsonite, Gypsum
UC6742a	O (65.87) C (26.59)	Zn (2.98) S (2.59)	Pb (0.96) Cl (0.27) Ca (0.25) Si (0.23) Mg (0.14) Fe (0.12)	Galena, Quartz, Smithsonite, Anglesite, Zinc Sulphide, Phosgenite
UC7890a	O (66.46) C (33.09)		Na (0.34) Cl (0.03) Mg (0.02) Al (0.02) S (0.01) K (0.01) Ca (0.01)	Natroxalate, Whewellite, Glushinskite
UC7890b	C (42.30) O (37.93)	Si (8.36) Cu (3.98) Al (2.01) Cl (1.89) Ca (1.86)	K (0.55) Fe (0.38) P (0.29) Mg (0.28) S (0.16)	Quartz, Calcite, Atacamite, Whewellite
UC43107	O (59.41) Si (17.94)	Al (6.12) Cu (4.00) Ca (3.28) Na (2.18) Cl (1.75) K (1.69) Fe (1.65) Mg (1.04)	P (0.49) S (0.30) Ti (0.16)	not enough material left for PXRD analyses
UC31613	O (42.98) C (24.55) Mn (19.10)	Si (8.48) F (1.96)	Na (0.26) Mg (0.25) Al (0.86) Ca (0.70) K (0.10) Cu (0.21)	Manganite, Quartz, Biotite, Pyrolusite

#### Major components

The samples can be subdivided into four groups based on their major components identified by SEM/EDS analyses. First, three samples are based on manganese as well as carbon and oxygen as major components (UC31613, UC43078, UC43148). In all three, the manganese can be attributed to manganite (MnO(OH)) identified via PXRD (Supplementary Figs. S4, S19, and S24). UC31613 additionally yielded pyrolusite (MnO_2_). Secondly, two samples (UC64751, UC7321) contain sulphur and lead as major components. The PXRD analysis shows, that both samples contain galena (PbS) and cerussite (PbCO_3_) (Supplementary Figs. S29 and S39). In addition, UC64751 contains laurionite (PbCl(OH)) and UC7321 contains phosgenite ((PbCl)_2_CO_3_) and litharge (PbO). The presence of laurionite and phosgenite in Ancient Egyptian make-up likely stems from synthetic manufacture^11,13^. Thirdly, one sample is based on silicon and oxygen (UC43107). The FTIR spectrum of UC43107 (Supplementary Fig. S56) is consistent with silicates and possible aluminosilicates^27,28^. This sample could not be further characterised by PXRD due to a lack of material. The fourth group includes six samples (UC43159, UC42810, UC46348, UC6742a, UC7890a, UC890b) for which the main components are carbon and oxygen with relatively low amounts of other elements (Table 1). These findings highlight the likely organic carbon-based fraction of the vessel’s contents. Carbon black (amorphous phase) can be identified using Raman spectroscopy, but the technique was unavailable for this project^29^.

#### Minor components

Lead was found as a minor component in two samples (UC43159, UC7321). Both were found to contain a variety of lead minerals, including the likely synthetic phosgenite in UC7321 and laurionite as well as cotunnite (PbCl_2_) in UC43159 (Supplementary Figs. S39 and S9). Notably, UC43159 does not contain galena, which is relatively rare for lead-containing kohls (Table 1). Zinc was found as a minor component in two samples (UC6742a, UC7321). It occurrs as smithsonite (ZnCO_3_) and zinc sulphide, which is dark in colour in its unpurified form (Supplementary Figs. S44 and S39). Zinc and lead are commonly associated in ores^30^, which may also explain its presence in the lead-based UC7321. Silicon was identified as a minor component in three samples (UC31613, UC42810, UC43148). In all three, the presence of silicon is consistent with quartz identified via PXRD (Supplementary Figs. S4, S14, and S24). Furthermore, biotite was found in UC31613. This mineral has not previously been identified in kohls. Copper was identified in UC7890b and UC43107, alongside the elements commonly identifiable in copper-based pigments and chloride minerals, which may include oxides of aluminium, calcium, iron, magnesium, and sodium^31^. The combined presence of elemental copper and chlorine in UC7890b is consistent with the FTIR and PXRD identification of atacamite (Cu_2_Cl(OH)_3_) (Supplementary Figs. S52 and S53). While this mineral occurs naturally, atacamite is a known degradation product of malachite and has been found alongside this mineral pigment on Ancient Egyptian objects^32^. It is thus possible that the original green pigment was malachite; this finding is also consistent with previous records noting the use of this ingredient as a cosmetic from predynastic times to at least the Nineteenth Dynasty^4^ and has been found in kohl^2^. Calcium that may be attributed to calcite was found as a minor component in three samples (UC7890b, UC42810, UC43107). In the case of UC42810 and UC43107, it is possible that it was introduced as the result of contact with the inside of the vessel.

#### Trace components

The trace components exhibit the most varied range of elements, with relative abundances varying from sample to sample (Table 1). The discrepancies between trace elements identified via SEM/EDS and elements present in minerals found using PXRD can be explained by small sample sizes, comparatively low sensitivity of PXRD and likely some heterogeneity within the samples. The trace elements include aluminium, calcium, chlorine, copper, iron, lead, magnesium, manganese, phosphorus, potassium, silicon, sodium, sulphur, titanium and zinc. Many of these elements are probably derived from ore impurities associated with lead minerals, manganite, pyrolusite, and atacamite employed in the kohl recipes.

Lead was identified as a trace component in three samples (UC42810, UC43148, UC6742a). The PXRD analysis revealed some lead components that are likely synthetic^11,13^ for two of these objects: phosgenite in UC6742a and laurionite in UC42810 (Supplementary Figs. S14 and S44). Furthermore, paralaurionite (PbCl(OH)), which is dimorphic with laurionite, was identified in UC42810, representing the first known find of this mineral in kohl. The sample from UC43148, containing manganite as major mineral, also yielded galena and litharge (Supplementary Fig. S24). These lead minerals are of plausible natural origin but are not consistent with known manganite ores^33^. The trace amounts of lead minerals may have been intentionally added or they may be the result of cross-contamination during the production process. Either way, their presence may be indicative of admixing several minerals to produce one eye ointment, as written records show that they were often assembled according to complex prescriptions^9^﻿.

Iron was found as a trace element in six samples (UC43159, UC42810, UC46348, UC7321, UC6742a, UC7890b) although it could not be matched with any minerals using PXRD. Iron often co-occurs with manganese in ores^33^ and occasionally with lead^34^. In one sample (UC64751), hematite (Fe_2_O_3_) was found by PXRD (Supplementary Fig. S29), but no iron was detected via SEM/EDS. This is likely due to heterogeneity within samples.

Several minerals were identified in kohl for the first time. In UC46348, lizardite and talc were found (Supplementary Fig. S34). The elemental composition of the sample suggests that both are present in trace amounts. The two samples from separate tubes in the wooden kohl pot (UC7890a, UC7890b) yielded oxalate minerals (Supplementary Figs. S49 and S53), which are often found as the result of oxidative degradation of organic compounds^35,36^.

All samples coming from calcite containers (UC43159, UC42810, UC43078, UC43148, UC31613) contain minor to trace amounts of calcium. The presence of calcite was confirmed using PXRD in four of the five (UC43159, UC42810, UC43078, UC43148). It cannot be excluded that some of the calcite identified is contaminations from the vessel itself, although care was taken to avoid scratching the vessel, concentrating on loose material during the sampling process. The elements that make up the mafic vessel UC46348, a silicate rich in iron and magnesium, are also present in the sample. It may be the source of the identified lizardite, which may form from mafic^37^. No silicon was found in UC7321 despite the vessel consisting of the silicate material serpentine. The marl vessel UC43107 contains calcite and silicate; both calcium and silicon are present in UC43107 as major or minor components. Based on the variations between the vessel composition and the identified trace, it cannot be conclusively determined whether these trace components are part of the kohl or stem from the vessel. In the former case, a mineral containing the elements may have been intentionally added to the kohl or it may have been introduced during mixing or grinding processes.

#### SEM imaging

The shape of particles was also observed during SEM analyses. Particle sizes across the samples range between 5 and 400 μm. Images can be found in Appendix 2 in the Supplementary Information. The particles’ shapes also vary across samples. The particles for UC64751 are quite angular which may be consistent with the cubic structure of galena also identified via PXRD (Supplementary Figs. S27 and S29). This substance tends to form single crystals also when milled^38^.

### Identification of organic ingredients

#### Plant-derived organic compounds

The majority of samples analysed yielded mid-length even-chain monocarboxylic acids, notably C_12:0_, C_14:0_, C_16:0_, C_18:0_, as well as even short-chain dicarboxylic acids, namely C_4:0_, C_6:0_, and C_8:0_. Azelaic acid (C_9:0_ dicarboxylic acid) is also present in the majority of samples, suggesting a high degree of degradation^39^. While these compounds are not indicative of any specific organic source, they likely derive from a series of plant oils based on high abundances of C_16:0_ and the absence of odd-chain monocarboxylic acids^40,41^.

**Figure 2:**
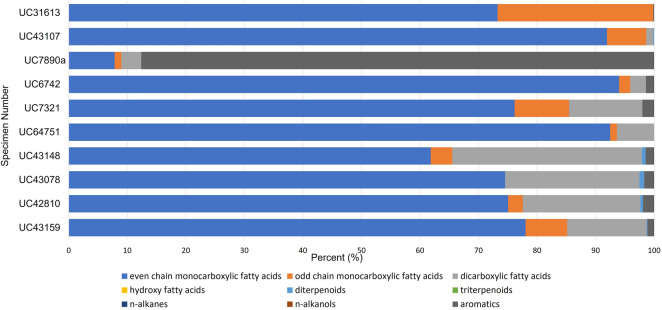
Percent distribution of main organic constituents identified by GC/MS. Chromatograms and detailed peak identification are described in Appendix 2 in the Supplementary Information.

Di- and triterpenoids were identified in the acidic and/or neutral fractions of five samples—UC43159, UC42810, UC43078, UC43148, UC43107—all in relatively low abundance (Fig. 2). The sample from UC42810 yielded a combination of dehydroabietic acid (DHA) and 7-oxo-DHA. These compounds, although not a complete suite of oxidised abietanes, are indicative of the presence of exudates from *Pinaceae* species^42,43^. DHA was also identified in isolation in UC43159, UC43078, and UC43148. Its presence alone has often been interpreted as derived from outside sources (i.e. wood smoke), and may not be due to the presence of resinous exudates^44^. Sample UC43107 yielded trace amounts of the triterpenoids alpha-amyrin and beta-amyrin. However, these compounds are not exudate-specific biomarkers.

**Figure 3:**
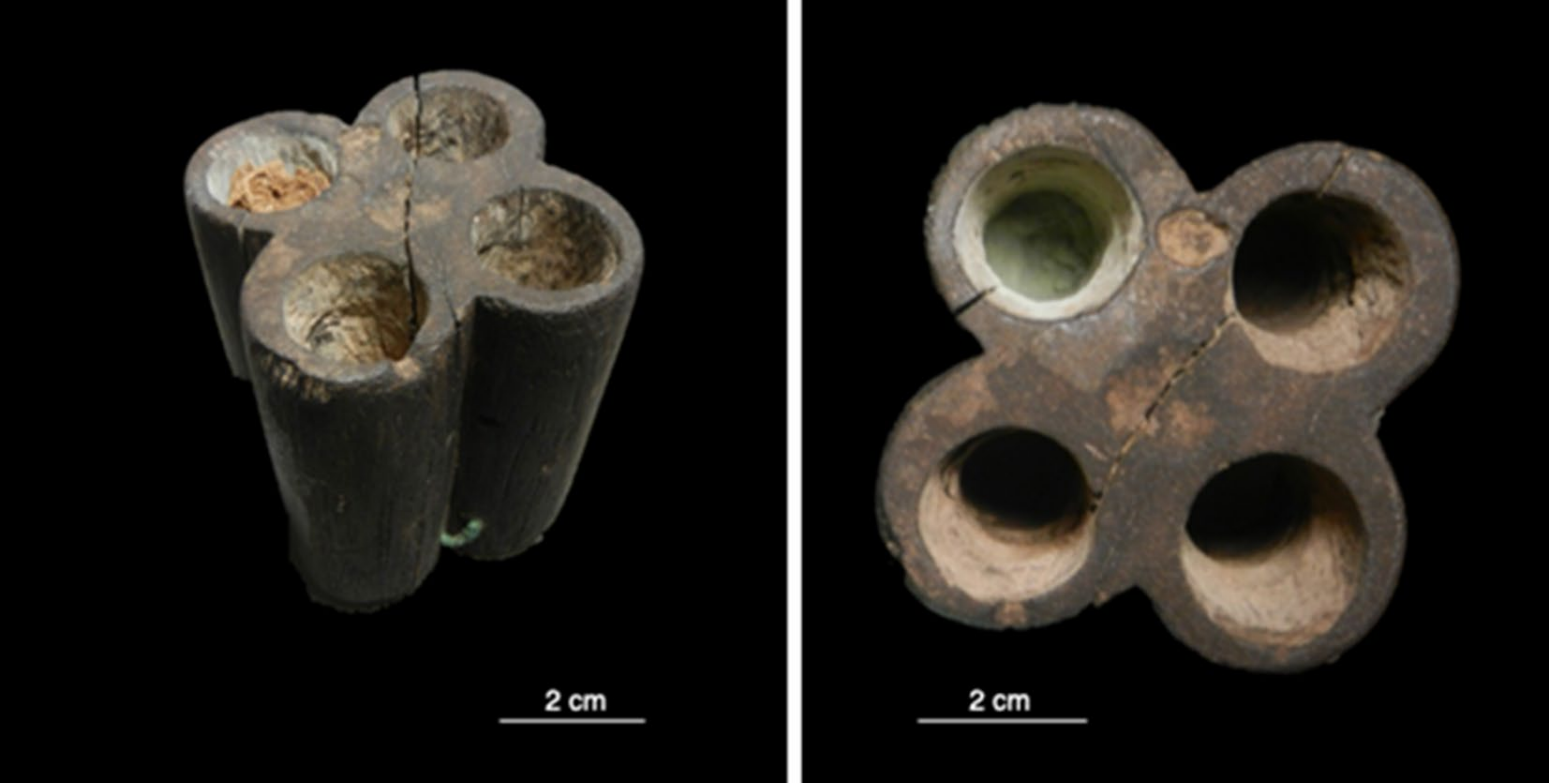
Photographs of the wooden kohl container UC7890 showing the 4 cavities for kohl powder, two of which still contained material that was analysed in this study. Image credit: Courtesy of the Petrie Museum of Egyptian Archaeology, UCL.

Sample UC7890a yielded benzoic acids, cinnamic acids, vanillic acids, resorcinol, syringic acid and ferulic acid. These compounds are the major components of benzoe resin, also known as gum benzoin, which comes from the Styraceae family, and a common resin in the Mediterranean area during antiquity^45^. However, benzoin resins have higher abundances of cinnamic acid to vanillic acid and the reverse is present in sample UC7890a, possibly indicating that these compounds do not derive from a benzoe resin. These compounds along with triterpenoid acids with an oleanane structure are also present in storax resin, another common Mediterranean resinous material^45^. Without additional triterpenoid acids, UC7890a cannot confidently be identified as storax-type resin. With no other additionally characteristic biomarkers, it is also possible that this sample contains an unknown aromatic plant exudate, likely from a balsam type plant. However, these markers are similarly associated with lignin and cellulose and are consistent with a plant-derived material^46–48^. Considering that this sample was obtained from UC7890, a wooden kohl pot, and without invasive sampling of the container itself, it cannot be ruled out that the aromatic compounds identified may be derived from the wood container itself and were diffused into the sampled kohl over time (Fig. 3).

#### Animal-derived organic compounds

Three samples (UC43148, UC46348 and UC6742) yielded saturated odd-chain monocarboxylic acids (C_15:0_ and C_17:0_), suggesting the presence of animal fats (Fig. 2). UC6742 may contain trace amounts of beeswax, based on the presence of both odd-numbered long chain *n*-alkanes (C25, C27, C29), with C27 being the most abundant, and 14-hydroxy- and 15-hydroxyhexadecanoic acid in relatively low amounts. Similar results have been identified in Egyptian mummification balms and Etruscan and later Roman ointments^49–52^.

#### Other compounds

All samples were screened for common biomarkers associated with source-specific or taxonomically specific material often found in archaeological contexts in Egypt and the surrounding regions of the Mediterranean area (Supplementary Table S13).

Six samples contain higher amounts of aromatic compounds in addition to various fats and oils of plant origins (UC43159, UC42810, UC43148, UC7890a, UC7321, UC46348). This ranges from 2 to 36% of the total extracted organic residue (Fig. 2). These compounds do not correspond to any known biomarker associated with Mediterranean resins (Supplementary Table S13). The presence of aromatics could be attributed to plant exudates being mixed into plant and animal fats for scent, binding and/or antibacterial purposes^53^. Lucas and Harris mention that oils and fats used in cosmetics were frequently scented, except when employed by the poorer classes^17^. Sample UC7890a contains the highest and most diverse suite of aromatic compounds. This sample, however, comes from a wood container. It is therefore possible that aromatics from the wood container have diffused into the organic binding medium over time. More research into the type of wood used for this kohl container would be required to confirm this hypothesis.

Eight unknown compounds were also observed in the samples analysed. Their corresponding mass spectra are reported in the Supplementary Information (Supplementary Table S14, Supplementary Fig. S58–S64). Each of these spectra has been compared with existing literature, including common biomarkers associated with source-specific or taxonomically specific material commonly found in archaeological contexts in Egypt and the Mediterranean and no match has been made. The majority of these unknown compounds are in low abundances, and in a small number of samples, with the exception of Unknown 1 (Supplementary Fig. S58). It is present in 8 samples and has a similar structure to azelaic acid.

## Discussion

This chemical study of Ancient Egyptian kohl vessels from the Petrie Museum collection has significantly expanded the known breadth of materials used to manufacture these cosmetics valued for both ritual and medicinal purposes throughout the Ancient Egyptian chronology. Indeed, the analytical results of this study on eleven kohl containers revealed a surprising variety of both inorganic and organic materials.

**Figure 4:**
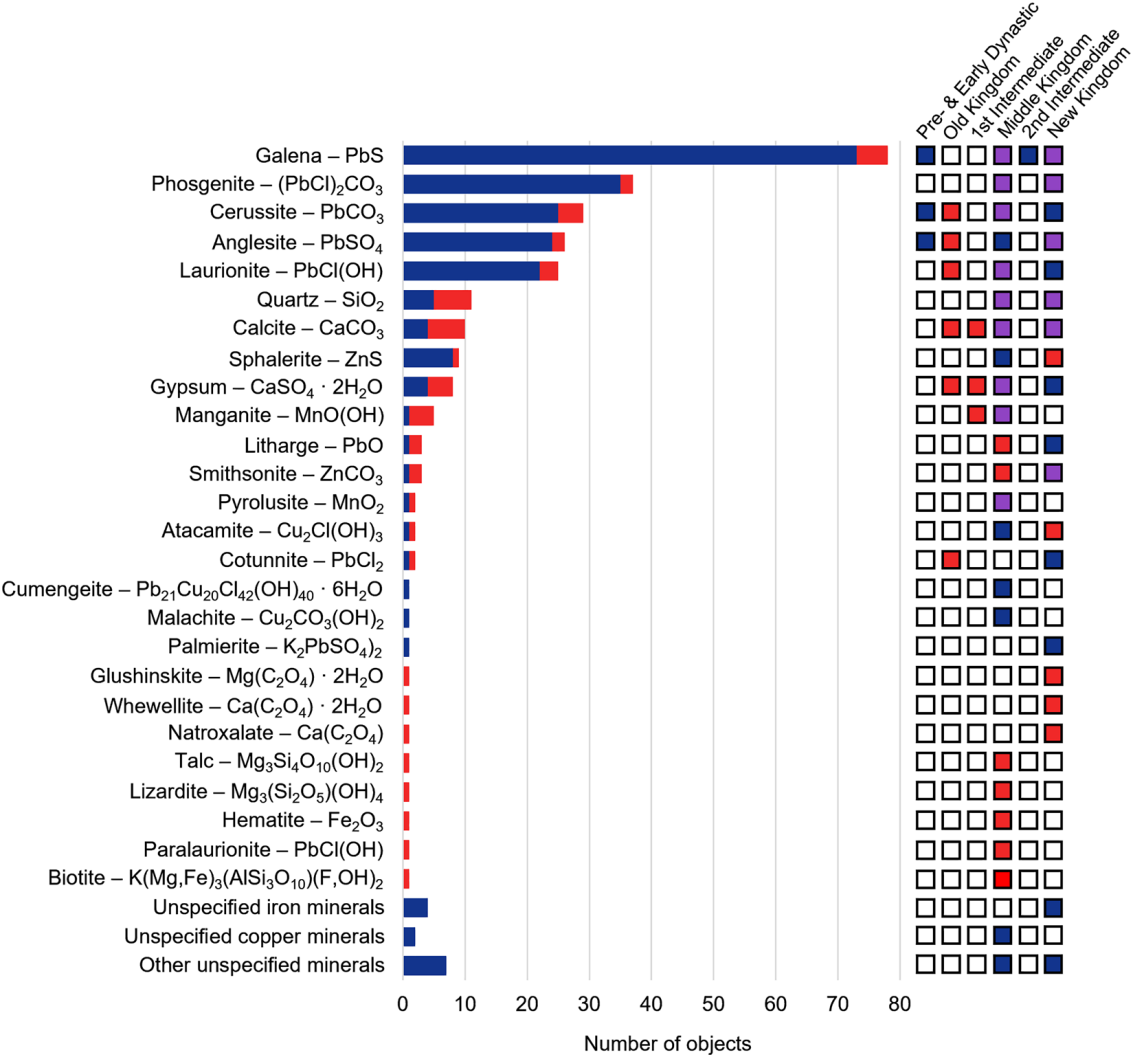
Overview of the inorganic constituents identified in kohls analysed since 1930, indicating the number of objects in which the constituent was identified in at least one analysis (see Supplementary Table 1 for details). The boxes on the right indicate the time periods of the kohl samples in which the listed ingredients were found based on literature (blue), this project (red), both (purple).

From an inorganic perspective, chemical analyses in the last few decades have identified a predominance of galena and other lead-based compounds in black kohls (Fig. 4, Supplementary Table S1). Of the 87 kohls that underwent inorganic compositional analysis by modern methods (synchrotron spectroscopic techniques, low voltage scanning electron microscopy [LVSEM], and quantitative evaluation of minerals by scanning electron microscopy [QEMSCAN]), 85 yielded lead-based mineral as the main component^6,10–16^. This includes naturally occurring minerals such as galena (PbS), cerussite (PbCO_3_) anglesite (PbSO_4_), litharge (PbO) as well as synthetic products such as laurionite (PbOHCl) and phosgenite (Pb_2_Cl_2_CO_3_). Manganese-based kohl, made of pyrolusite (MnO_2_) and manganite (MnO(OH)), in a lead-based mixture, has been identified in a single study^15^. Green kohls are similarly infrequent in published studies. These specimens have been found to contain malachite (Cu_2_[(OH)_2_|CO_3_]), cumengeite (Pb_21_Cu_20_(OH)_40_Cl_42_·6H_2_O), atacamite Cu_2_Cl(OH)_3_ and gypsum (CaSO_4_)^10^.

The variety of kohl ingredients identified in this study on the Petrie Museum Collection provides a contrast to previous analytical findings. We identified two lead-based, one silicon-based, three manganese-based, and six carbon-based kohl specimens. Interestingly, the range of inorganic ingredients in kohls identified in this study is more reminiscent of earlier chemical analyses, which revealed a larger variety of ingredients with only 47 out of 75 samples being lead-based (Supplementary Fig. 65)^17^.

The variability in inorganic materials identified in this study is exemplified in four ways. First, our chemical analyses allowed us to identify eight minerals that had not previously been found in kohl: biotite, paralaurionite, lizardite, talc, hematite, natroxalate, whewellite and glushinskite. Although some of these constituents may not have been added intentionally, the overall picture painted by our analyses remains much more diverse than previous analyses show. We also identified constituents that had very rarely been found in previous studies such as manganite, litharge, and smithsonite. The case of UC43159 provides especially strong evidence of admixing several minerals, as it contains both synthetic as well as naturally occurring lead salts in addition to manganite. Secondly, UC43078, UC43148 and UC31613 present rare evidence for the use of manganese-based materials in kohls, specifically manganite and pyrolusite. Previously, only one such sample had been analysed with modern methods, which furthermore contains considerably more lead than the specimens analysed in this study^15^. Thirdly, kohls that are high in elemental carbon (from carbonates and/or organic materials) have hardly been reported previously. In our study, the analysis of UC46348 revealed that a proportion of carbon is derived from animal source(s). Lastly, no Ancient Egyptian kohl consisting largely of silicates has previously been recorded. The silicon-based sample (UC7890b) is also exceptional in its green colour (Fig. 3), which can be attributed to atacamite identified by FTIR and PXRD. The addition of silicate materials may have been intentional, especially when present as a major constituent. However, it is also possible that they were introduced accidentally in the manufacturing process, due to conditions on site or during the excavation.

It is important to acknowledge that the differences observed in the inorganic composition of kohl recipes may have different origins such as the context of previous studies and the provenance of the samples as well as the difference within the techniques used and their specificities. Indeed, the research objectives may have been different (e.g. look at the crystalline content or specific elements). Some techniques are more suitable for tracking certain constituents (potentially increasing their importance in described recipes), while being unable to identify others. In our study, we opted for a combined use of FTIR, SEM/EDS, PXRD and GC/MS, allowing for a more holistic approach to the characterisation of materials possibly present in the objects analysed.

Additionally, the recipes used to produce cosmetics in Ancient Egypt may have changed with time and location. Results of this study and previous analyses show that the variety of materials is spread across both chronologically and geographically (Fig. 4, Supplementary Figs. S66, S67, S68, and Supplementary Table S1). No clear pattern of material use emerges. It must be noted that recently analysed specimens do not evenly represent archaeological time periods or site locations. Samples from the New Kingdom are especially over-represented. Apart from the samples analysed in this study, no analytical data is available on kohl samples from the Old Kingdom and 1st Intermediate Period. Notably, our analyses show that the synthetic component laurionite^11^ was already used in the Old Kingdom, which is much earlier than previously known.

The narratives of kohl production have largely been centred on inorganic minerals in previous case studies. In contrast, our study represents the first systematic study of organic components in kohls. It yielded six (out of eleven) specimens that likely consist predominantly of organic materials. All samples bar one (UC7890a) contain plant oils; three samples additionally contain animal-derived fat. Animal fats are consistent with written records, which indicate that a variety of animal extracts were used in eye ointments, most commonly goat, but also pig, tortoise, vulture and others^9^. Taxonomically distinctive ingredients identified in this study included *Pinaceae* resin and beeswax. These findings are also consistent with written records, which indicate that Ancient Egyptian kohl and eye ointments did include organic components, among them resins, plant extracts, leaves and seeds^9^. Overall, our findings indicate that organic materials are much more common in kohl recipes than previously suggested^17^. The function of all ingredients (organic and inorganic) employed in kohl recipes remains uncertain as there are health benefits (both actual and ritual) beyond the aesthetic purpose of these cosmetics^1^.

## Conclusion and perspectives

Our multi-analytical study of the contents of eleven containers indicates that kohls were heterogeneous mixtures that were obtained following a variety of recipes much more complex than initially thought. The methodologies employed in this study allowed for the systematic characterisation of both organic and inorganic fractions from a single microsample.

Collating data on a large enough ensemble of kohl containers, alongside having robust geographical and chronological information, is crucial to start investigating with confidence possible patterns in kohl recipes in relation to their location and/or their time period. The case study at the Petrie Museum may be considered as a starting point to such endeavours.

Materials collected and/or produced in Ancient Egypt often had multiple utilisations. Taking the example of Egyptian blue, it has been used in mummy portraits^54^ as well as mural paintings^55^. Similarly, organic materials such as oils, fats, resins, waxes, gums, and proteinaceous materials were utilised by Ancient Egyptians for medicinal, ritual, aesthetic, and quotidian needs in their current and afterlives^36,38,41–46^. This study shows that kohls, if analysed by robust multi-analytical approaches, can also reveal a range of materials that have been used in Ancient Egypt. They could lend, for example, additional perspective to aspects of pigments and binders used in Ancient Egypt, where the parallels in utilised materials and technical studies are comparatively small in number for an archaeological chronology spanning several thousand years^56^.

## Materials and methods

### Objects

Eleven kohl-containing vessels from the Petrie Museum collection were selected for analyses (details in Supplementary Table S15 and Appendix 2). Those selected objects contained enough residue that micro-sampling would not affect the overall appearance of the object. Of these, nine are pots made from inorganic materials (see Supplementary Table S15) while the objects UC6721 and UC7890 were made of horn and wood, respectively.

### Sampling

Samples of < 1 mg were removed with a scalpel from the interior of the vessels, with preference to naturally loose, fragmentary or cracked surfaces of the residue deposits.

### Chemicals and standards

All solvents used were of HPLC analysis or residue analysis grade. N,O-bis(trimethyl)silyltrifluoroacetamide (BSTFA) containing 1% trimethylchlorosilane (TMCS) was used for derivatisation while tridecanoic acid (IS1) and hexadecane (IS2) were used as internal standards.

### Methods

#### FTIR

The measurements were acquired on an Agilent Cary 640 spectrometer with a GladiATR Single Reflection diamond ATR accessory. Analyses were performed in absorbance mode, with spectra collected from 4000 to 400 cm^−1^ with 64 acquisition scans and a resolution of 4 cm^−1^.

#### SEM/EDS

Few micrograms of the samples were analysed using a JEOL JSM-6610LV Scanning Electron Microscope with an Oxford Instrument EDS detector. All samples were uncoated. Images were acquired between 3 and 5 kV, with a spot intensity of 53, in secondary electron detector mode (SED). Elemental composition analyses were conducted at 20 kV with a spot intensity of 53 using the backscatter electron detector (BSE). The dimensions of the acquisition areas were adjusted based on features visible on the uncoated surface in BSE mode. Elemental composition was based on the acquired sum spectrum.

Spectra interpretation and image management were completed using Inca software (Oxford Instruments, UK).

#### PXRD

The mineralogical analyses of the kohl specimens were carried out by X-ray diffraction using a PANalytical X’Pert Pro diffractometer equipped with a cobalt tube (= 1.79 Å) running at 40 kV and 40 mA. Samples were deposited with a few microliters of ethanol on low background silicon plates and analysed from 5° (2) to 70° (2) with a step size of 0.013° and a total counting time of 8 h. Samples were also spun at 15 rpm to improve statistics.

Phase identification was performed using the X’pert Highscore plus software (PANalytical) together with the PDF-2 ICDD database (International Center for Diffraction data, Powder Diffraction Files 2).

Due to the low amount of material available for the analyses, the samples were not grinded, contrarily to what is traditionally done for powder X-ray diffraction, to avoid losing material. This leads to two difficulties for the identification of the minerals: despite a high counting time, the diffraction peaks are often low in intensity, and the grains do not present every orientation to the beam so some diffraction peaks may be missing, or intensities may be very different from those of the mineral database.

#### GC/MS

Samples underwent organic extraction using a saponification and acidification reaction^57^. The extracts, were derivatised with N,O-bis(trimethyl)silyltrifluoroacetamide (BSTFA) containing 1% trimethylchlorosilane (TMCS) before analyses. These analyses were performed using an Agilent 7820A gas chromatograph coupled to an Agilent 5975 quadrupole mass spectrometer. Samples were injected in splitless at 280 °C. GC separation was performed on a fused silica capillary column HP-5MS (J&W Scientific, Agilent Technologies, U.S.A, 30 m length × 0.25 mm ID × 0.25 μm film thickness, 5% diphenyl/95% dimethylpolysiloxane stationary phase). Chromatographic conditions were as follows: initial temperature 80 °C, 2 min isothermal hold, 10 °C/min up to 200 °C, 4 min isothermal hold, 6 °C/min up to 280 °C, 40 min isothermal hold (adapted from Ribechini et al*.*^58^). The helium (purity 99.9995%) gas flow was set in constant flow mode at 1.2 mL/min. MS parameters were: electron impact ionization (EI) of 70 eV; ion source temperature 230 °C; interface temperature 280 °C; and a scan range of 50–700 *m/z*. The injection volume was 2 µL. Peak areas were obtained through AMDIS automatic spectral deconvolution, in Simple analysis mode. Peak assignment for the GC/MS was based on comparisons to mass spectra libraries (NIST 11 main EI MS library and an AMDIS mass spectra library) and published data^19,39,42,43,45,51,59–69^ (Supplementary Table S13).

## Supplementary Information

Supplementary Information 1.

Supplementary Information 2.
