# Supplementary material for: Recipes of Ancient Egyptian kohls more diverse than previously thought
Source: Sci Rep. 2022 Apr 8;12:5932. doi: 10.1038/s41598-022-08669-0 (PMC8994005; doi:10.1038/s41598-022-08669-0)
Supplement: Supplementary file 1 — Supplementary Information 1. [file 41598_2022_8669_MOESM1_ESM.docx]

# Recipes of Ancient Egyptian Kohl More Diverse Than Previously Thought

Marabel Riesmeier^1,a,⸸,*^, Jennifer Keute^1,⸸^, Margaret-Ashley Veall^1,b,⸸^, Daniel Borschneck^2^, Alice Stevenson^3,c^, Anna Garnett^3^, Alice Williams^3^, Maria Ragan^3,d^, Thibaut Devièse^1,2,⸸,*^

*^1^ University of Oxford, Research Laboratory for Archaeology and the History of Art, Oxford, OX1 3TG, United Kingdom*

*^2^ CEREGE, Aix-Marseille University, CNRS, IRD, INRAE, Collège de France, Technopôle de l’Arbois, 13545, Aix-en-Provence, France*

*^3^ University College London, Petrie Museum, London, WC1E 6BT, United Kingdom*

*^a^ University of Cambridge, Department of History and Philosophy of Science, Cambridge, CB2 3RH, United Kingdom*

*^b^ Canadian Conservation Institute, Ottawa, Ontario, Canada K1B 4S7*

*^c^ University College London, UCL Institute of Archaeology, London, WC1E 6BT, United Kingdom*

*^d^ St Barbe Museum and Art Gallery, Lymington, SO41 9BH, United Kingdom*

^*^[marabel.riesmeier@gmail.com](mailto:marabel.riesmeier@gmail.com); [thibaut.deviese@univ-amu.fr](mailto:thibaut.deviese@univ-amu.fr)

^⸸^these authors contributed equally

**Appendix 1 (separate Excel sheet)**

***Previous analyses of ancient Egyptian kohl specimens***

Supplementary Table S1

**Appendix 2 6**

***Additional information and data for each object analysed in this study***

Supplementary Figures S1 – S57

Supplementary Tables S2 – S12

**Appendix 3 68**

***GC/MS data interpretation and unknown compounds***

Supplementary Table S13 and Table S14

Supplementary Figures S58 – S64

**Appendix 4 73**

***Patterns, Time periods and locations***

Supplementary Figures S65 – 67

**Appendix 5**

***Materials and methods* 74**

Supplementary Table S15 Accession records and description of samples

**Additional References 77**

**Appendix 2**

***Additional information and data for each object analysed in this study.***

Subsequent object figure captions are based on original accession records of the Petrie Museum and reflect the assessments given by F. Petrie at the time the object arrived in the collections. These descriptions are not indicative of results presented in this study.

**UC31613**

**
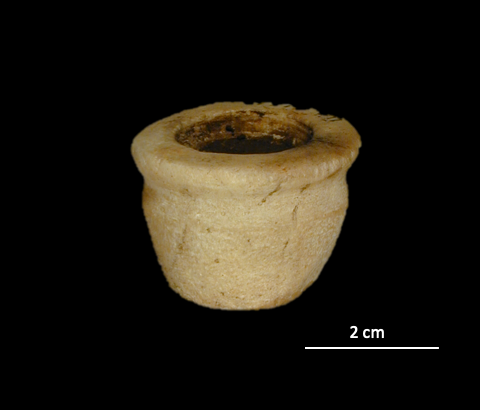
**

**Figure S1** Picture of a kohl pot (UC31613) from Diospolis Parva Cemetery, Middle Kingdom (Dynasty XII), kept at the Petrie Museum. Object description: Calcite thin brim kohl pot, with contents. Sample description: Residue sample taken from the inside, base of the vessel. Image credit: Courtesy of the Petrie Museum of Egyptian Archaeology, UCL.


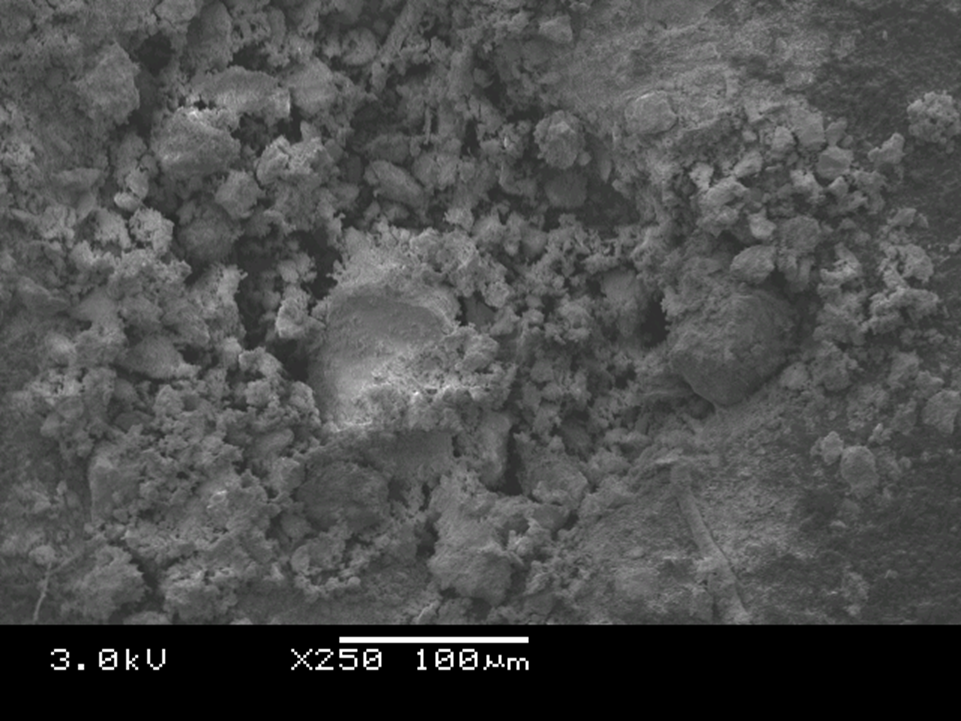


**Figure S2** SEM/EDS image of a microsample taken from the contents of the object UC31613. Images were acquired between 3-5 kV, with a spot intensity of 53, in secondary electron detector mode (SED). Elemental composition: Major: O (42.98) C (24.55) Mn (19.10) Minor: Si (8.48) F (1.96) Trace: Na (0.26) Mg (0.25) Al (0.86) Ca (0.70) K (0.1) Cu (0.21).


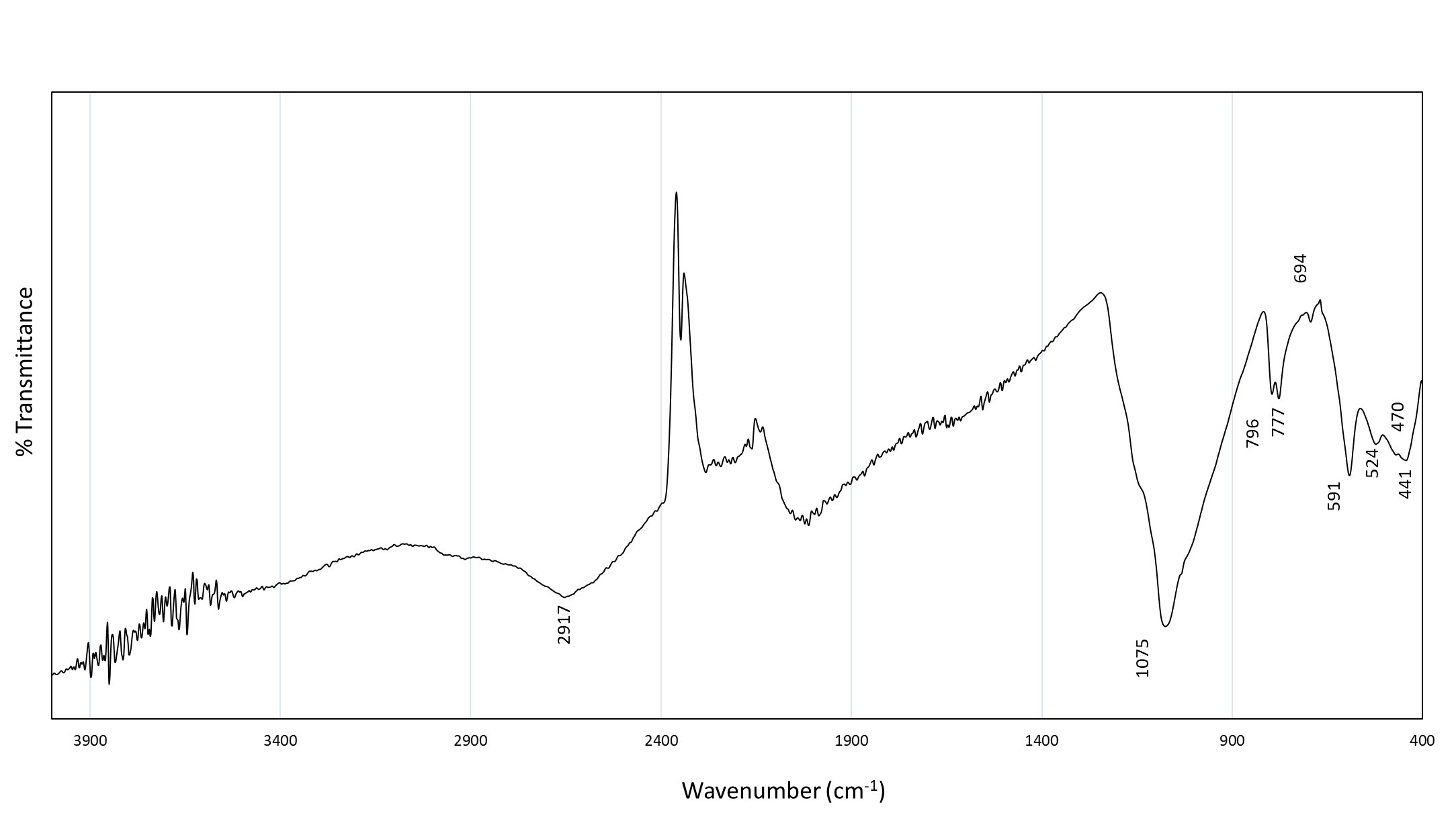


**Figure S3** FTIR spectrum of the sample from UC31613 displayed in percentage transmittance. Inorganic minerals from silicates (likely quartz) were identified by the presence of a broad band at 1075 cm^-1^, a doublet peak at 796 cm^-1^ and 777 cm^-1^, and a small peak at 694 cm^-1^. This is consistent with known references[^20^](https://paperpile.com/c/29uT4b/CiyO) (IRUG IMP00322).

**
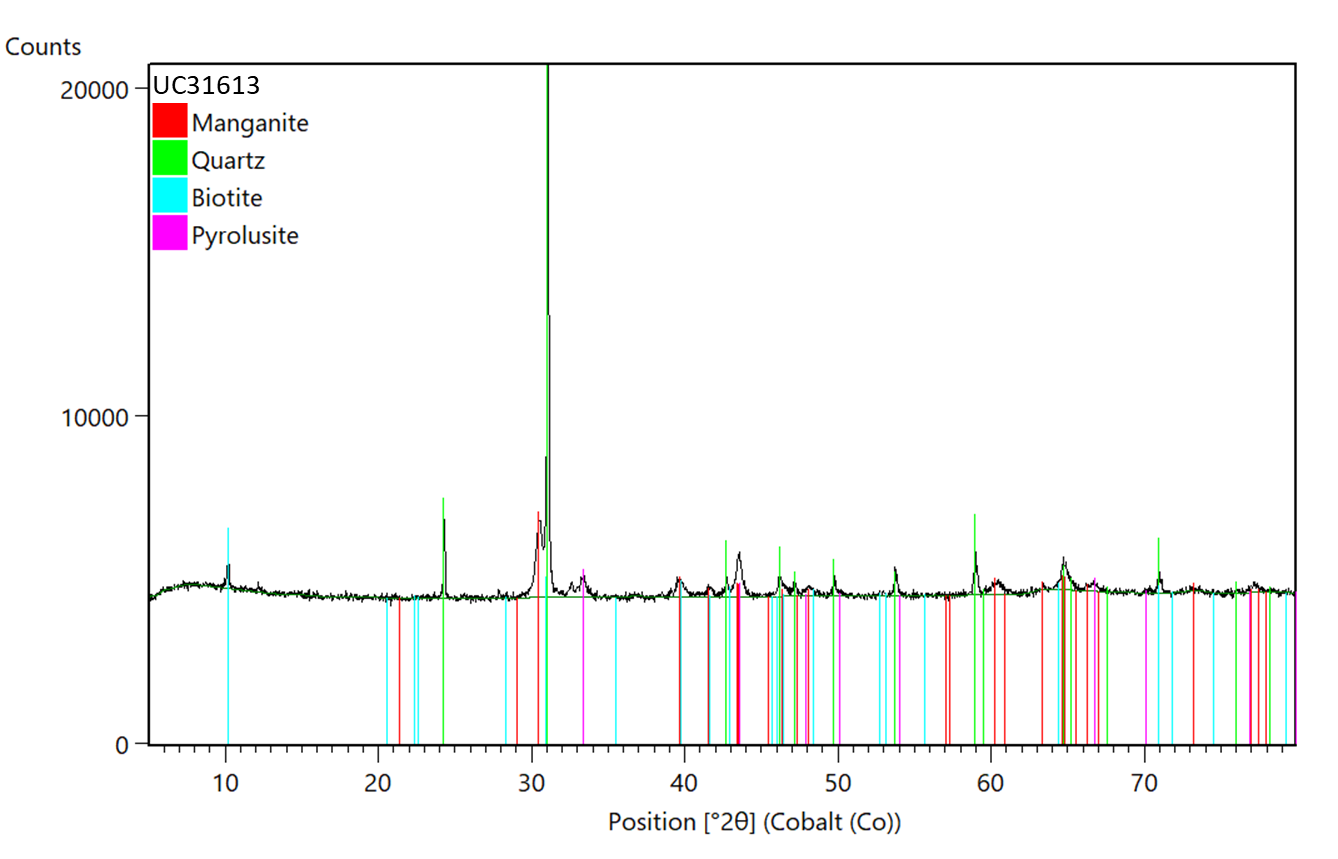
**

**Figure S4** X-ray diffractogram of the sample from UC31613. Manganite, Quartz, Biotite and Pyrolusite were identified.

**
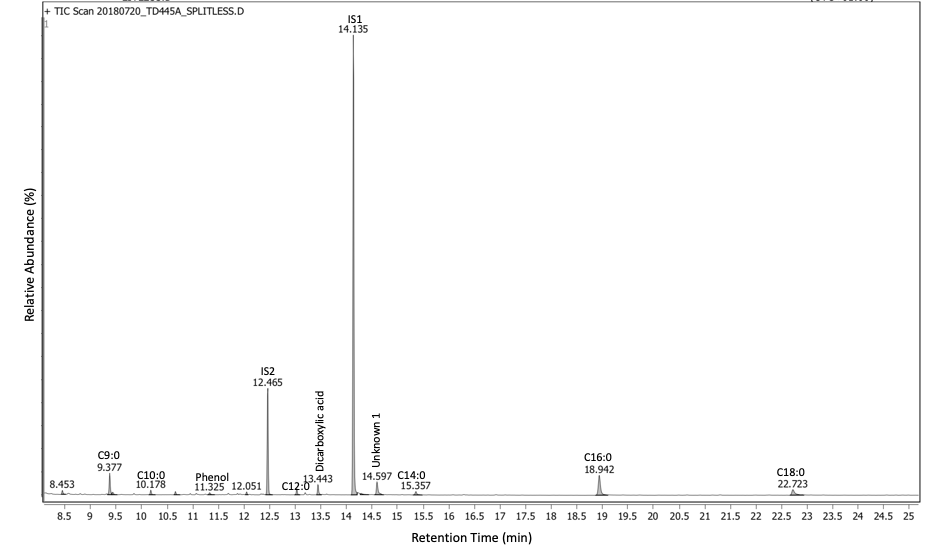

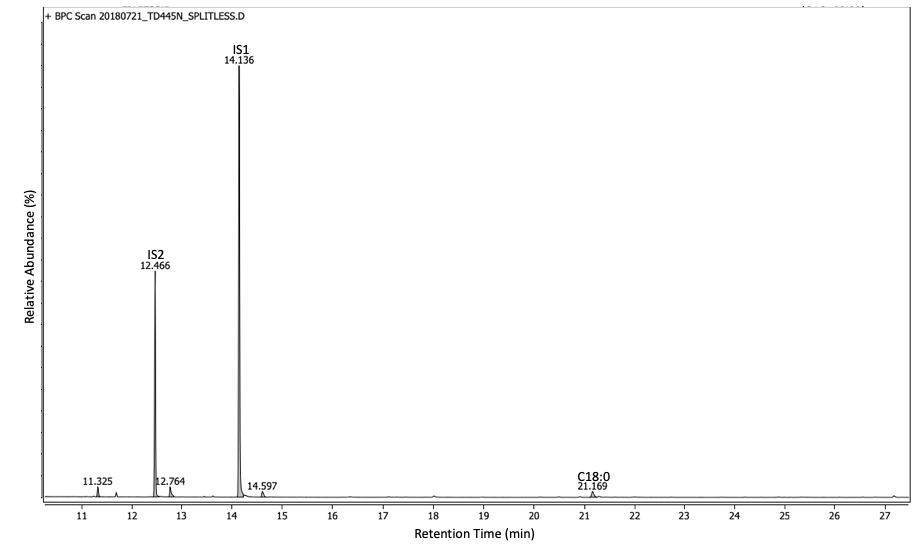
**

**Figure S5** GC/MS chromatograms obtained on the organic extract of the sample (UC31613). Top and bottom chromatograms correspond to the acidic and neutral fractions, respectively. Peak identifications are reported in Table S2.

**Table S2** Identification of the compounds present in the organic fraction of the sample (UC31613). TMS indicates a trimethylsilyl ester.

| **Time (min)** | **Compound** | **Peak area** |
| --- | --- | --- |
| *Acidic fraction* | | |
| 9.3779 | nonanoic acid, TMS | 977342 |
| 11.8748 | undecanoic acid, TMS | 27208 |
| 12.4647 | hexadecane (IS2) | 4724842 |
| 12.7444 | 4-benzoic acid, TMS | 6569 |
| 13.0323 | dodecanoic acid, TMS | 260776 |
| 14.1388 | tridecanoic acid, TMS (IS1) | 2101146 |
| 15.358 | tetradecanoic acid, TMS | 189936 |
| 18.9411 | hexadecanoic acid, TMS | 1748255 |
| 22.723 | octadecanoic acid, TMS | 603811 |
| 27.9842 | monopalmatin, TMS | 26815 |
| *Neutral Fraction* | | |
| 8.1398 | glycerol, TMS | 74166 |
| 9.3781 | nonanoic acid, TMS | 25117 |
| 12.4655 | hexadecane (IS2) | 6307588 |
| 14.1389 | tridecanoic acid, TMS (IS1) | 2380662 |
| 18.9555 | hexadecanoic acid, TMS | 23890 |
| 22.7299 | octadecanoic acid, TMS | 12678 |
| 27.1806 | diisooctyl phthalate | 73833 |

**UC43159**

**
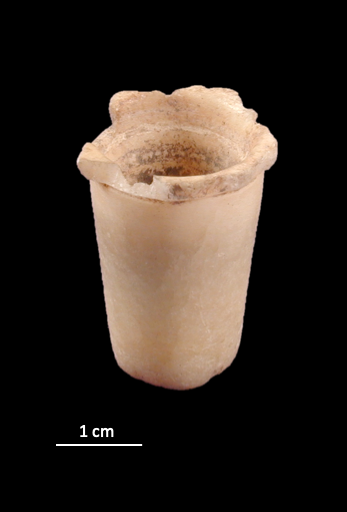
**

**Figure S6** Picture of a kohl pot (UC43159) from Abydos, Old Kingdom, kept at the Petrie Museum. Object description: calcite cylinder vase, everted rim, chipped and broken all around circumference, tapered to flat base, kohl on interior. Sample description: black residue observed, sample from inside vessel. Image credit: Courtesy of the Petrie Museum of Egyptian Archaeology, UCL.


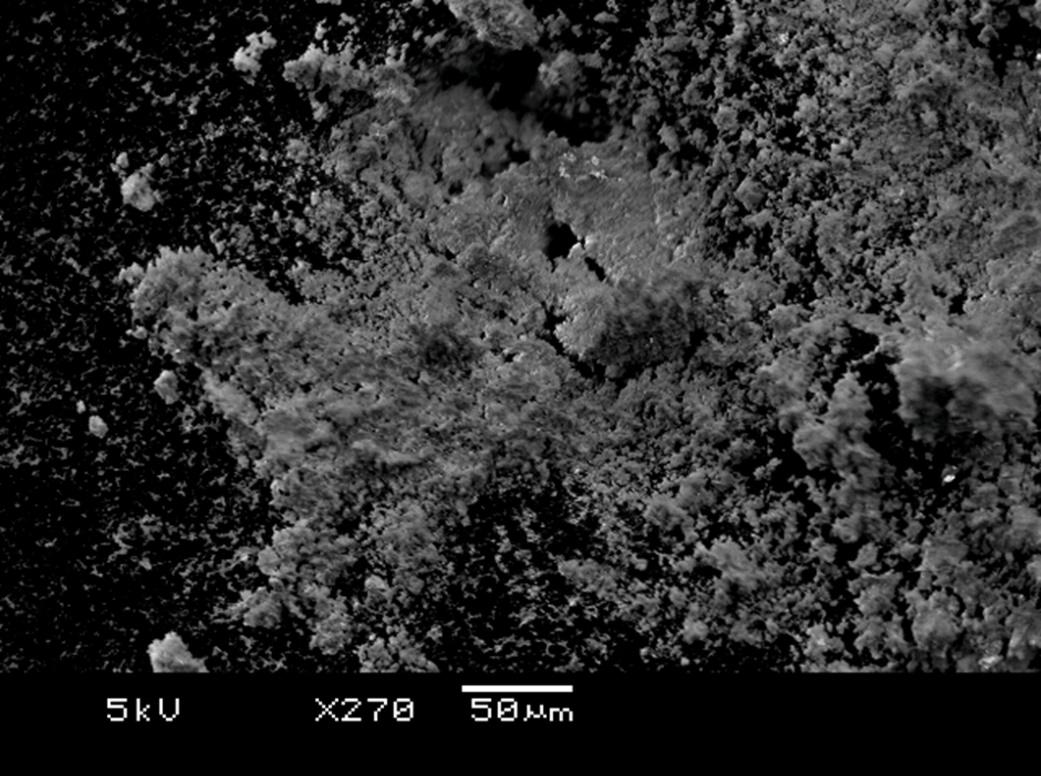


**Figure S7** SEM/EDS image of the sample taken from the content of the object UC43159. Images were acquired between 3-5 kV, with a spot intensity of 53, in secondary electron detector mode (SED). Elemental Composition: Major: O (65.27) C (28.71), Minor: Pb (1.68), Trace: Al (0.35) Si (0.61) P (0.82) S (0.26) Cl (0.66) K (0.08) Ca (0.51) Mn (0.78) Fe (0.14) Cu (0.13).


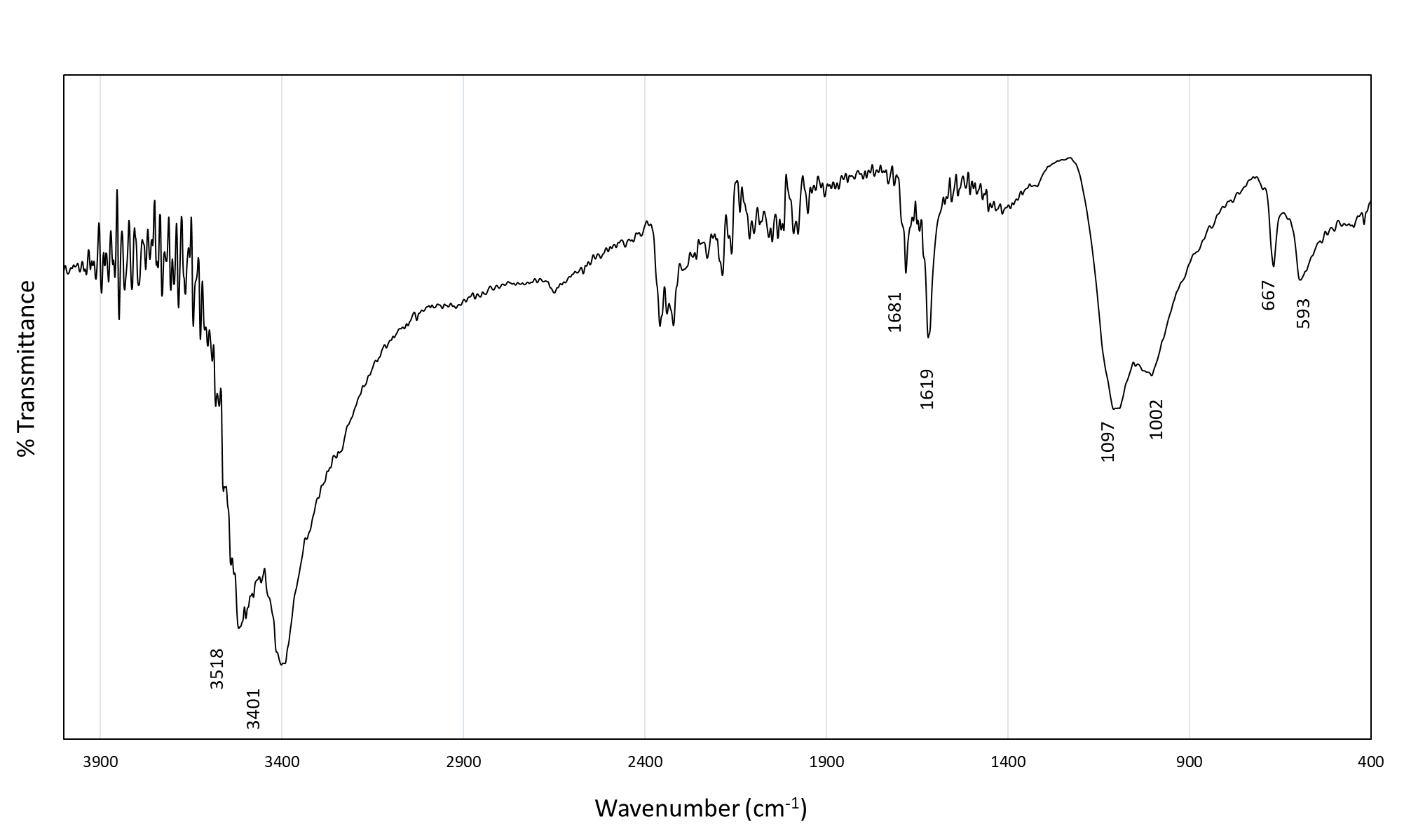


**Figure S8** FTIR spectrum of the sample from UC43159 displayed in percentage transmittance yielded peaks associated with inorganic materials. Peaks at 3518 cm^-1^, 3401 cm^-1^, 1681 cm^-1^, 1619 cm^-1^, 667 cm^-1^, and 593 cm^-1^ have been identified in references of gypsum[^18,19^](https://paperpile.com/c/29uT4b/1NCe+Rx5D). A relatively strong broad peak at 1097 cm^-1^ is partially due to gypsum but may be indicative of C-O-C stretching of esters and alcohols or is similarly within the range of C-O or Si-O stretching. The peak at 1002 cm^-1^ may be cyclohexane ring vibrations or has been identified in kaolin minerals.


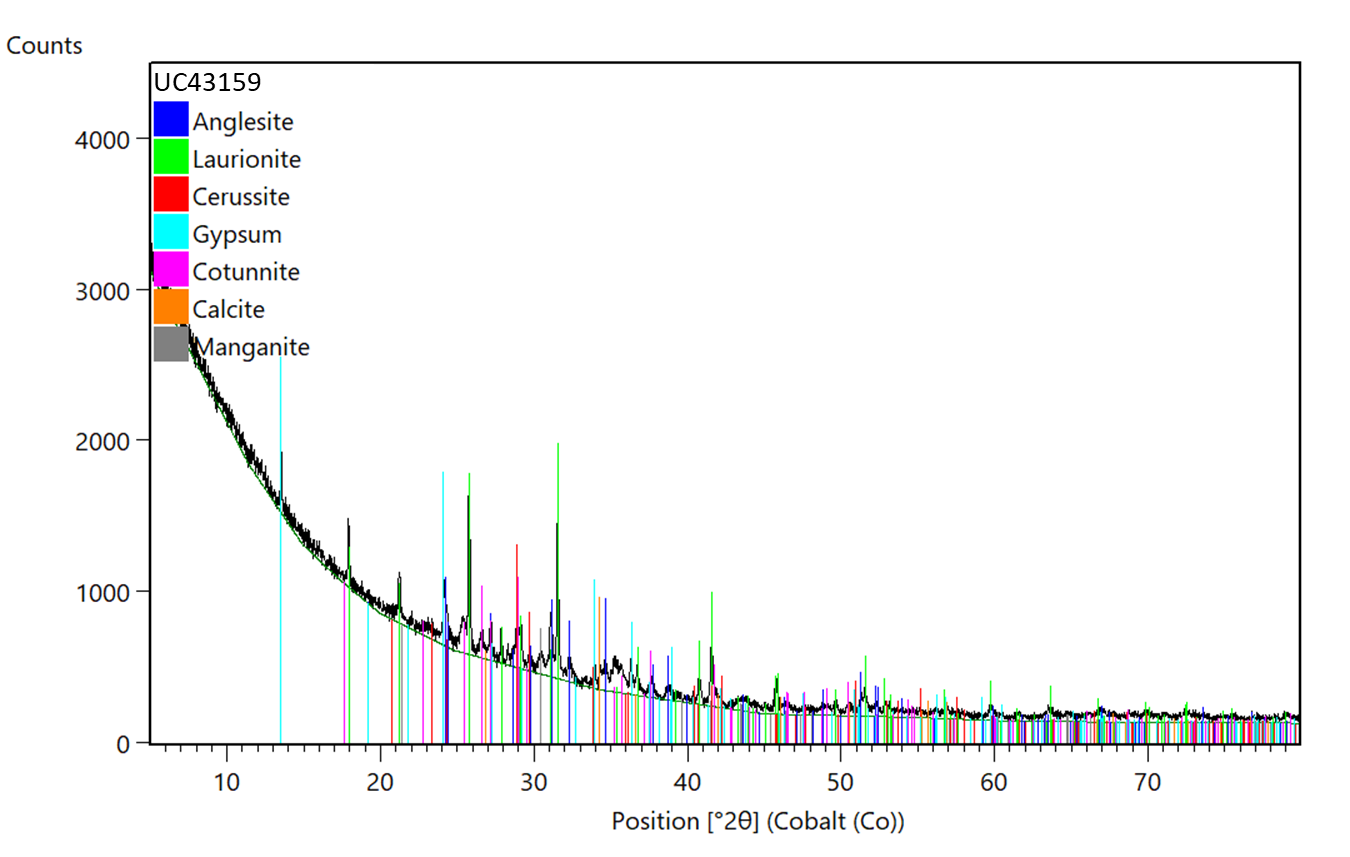


**Figure S9** X-ray diffractogram of the sample from UC43159. Anglesite, Laurionite, Cerussite, Gypsum, Cotunnite, Calcite and Manganite were identified.


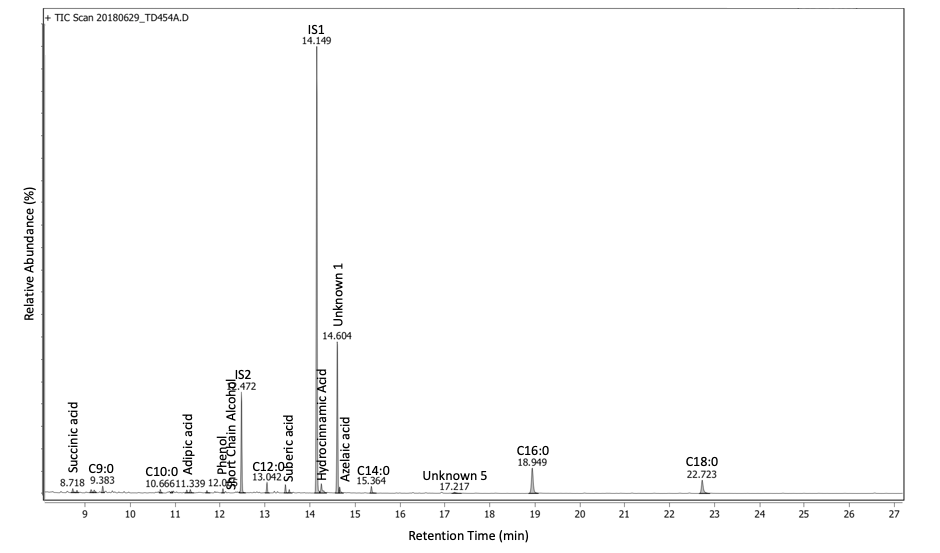

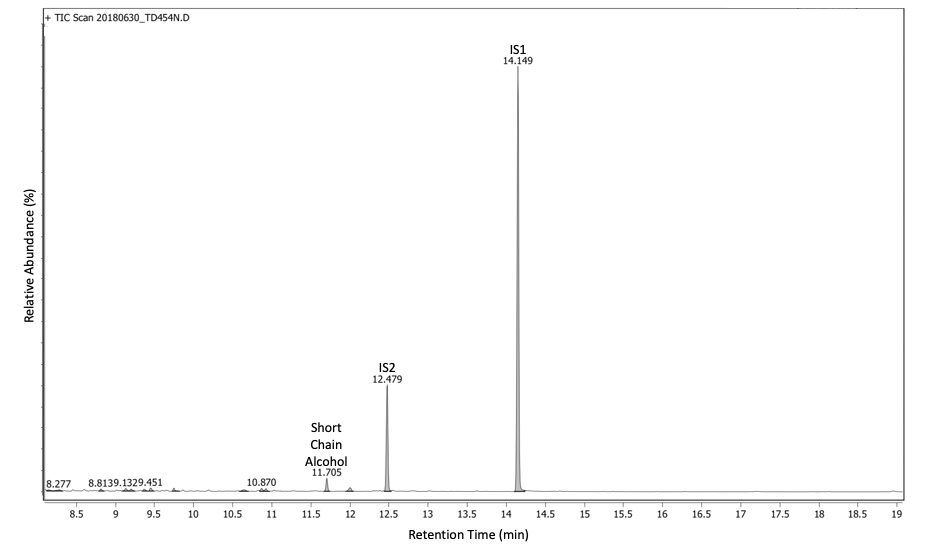


**Figure S10** GC/MS chromatograms obtained on the organic extract of the sample (UC43159). Top and bottom chromatograms correspond to the acidic and neutral fractions, respectively. Peak identifications are reported in Table S3.

**Table S3** Identification of the compounds present in the organic fraction of the sample UC43159. TMS indicates a trimethylsilyl ester.

| **Time (min)** | **Compound** | **Peak Area** |
| --- | --- | --- |
| *Acidic fraction* | | |
| 8.7201 | butanedioic acid, TMS | 325824 |
| 9.3874 | nonanoic acid, TMS | 506211 |
| 11.2591 | hexanedioic acid, TMS | 174782 |
| 11.8843 | undecanoic acid, TMS | 58241 |
| 12.453 | heptanedioic acid, TMS | 126192 |
| 12.4763 | hexadecane (IS2) | 8110570 |
| 12.7519 | 4-benzoic acid, TMS | 35540 |
| 13.0425 | dodecanoic acid, TMS | 751420 |
| 13.5414 | octanedioic acid, TMS | 214686 |
| 14.1511 | tridecanoic acid, TMS (IS1) | 40328031 |
| 14.28 | vanillic acid, TMS | 73983 |
| 14.617 | terephthalic acid, TMS | 63582 |
| 14.6595 | nonanedioic acid, TMS | 429988 |
| 15.3662 | tetradecanoic acid, TMS | 600401 |
| 15.9976 | decanedioic acid, TMS | 9528 |
| 16.932 | pentadecanoic acid, TMS | 95028 |
| 18.9431 | hexadecanoic acid, TMS | 3895284 |
| 20.91 | heptadecanoic acid, TMS | 13298 |
| 22.723 | octadecanoic acid, TMS | 2127490 |
| 24.9959 | DHA, TMS | 12588 |
| 27.2009 | diisooctyl phthalate | 6308 |

**Table S3** Continued.

| **Time (min)** | **Compound** | **Peak Area** |
| --- | --- | --- |
| *Neutral Fraction* | | |
| 8.1467 | glycerol, TMS |  |
| 12.4779 | hexadecane (IS2) | 10581152 |
| 14.1513 | tridecanoic acid (IS1) | 43875888 |
| 18.9569 | hexadecanoic acid, TMS | 125585 |
| 22.7226 | octadecanoic acid, TMS | 137355 |
| 27.1868 | diisooctyl phthalate | 268948 |

**UC42810**


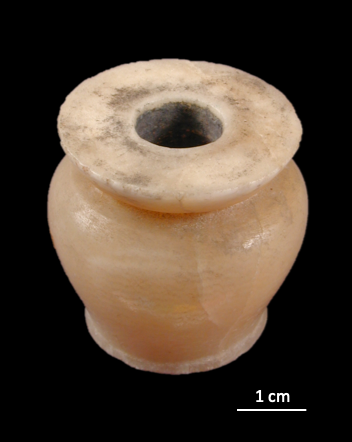


**Figure S11** Picture of a kohl pot UC42810 from Abydos, Middle Kingdom, kept at the Petrie Museum. Object description: Calcite kohl pot with kohl remaining inside and traces on flat topped rim, rim sloped in to keel above constricted neck, rounded shoulder body tapered to flat base with slightly projecting rim. Sample description: black residue observed, sample taken on interior of neck in the area of highest concentration. Image credit: Courtesy of the Petrie Museum of Egyptian Archaeology, UCL.


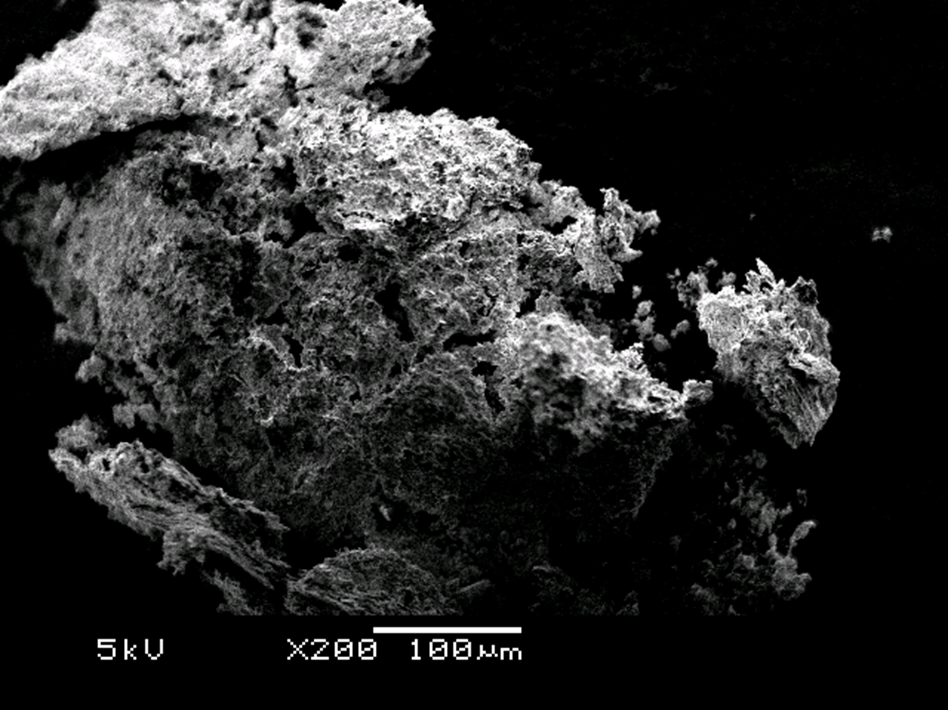


**Figure S12** SEM/EDS image of the sample taken from the content of the object UC42810. Images were acquired between 3-5 kV, with a spot intensity of 53, in secondary electron detector mode (SED). Elemental Composition: Major: O (65.35) C (28.92), Minor: Si (1.28) Ca (1.19), Trace: Pb (0.93) Al (0.53) Cl (0.38) Mg (0.35) Fe (0.34) P (0.24) S (0.18) Na (0.13) Mn (0.1) K (0.07).

**
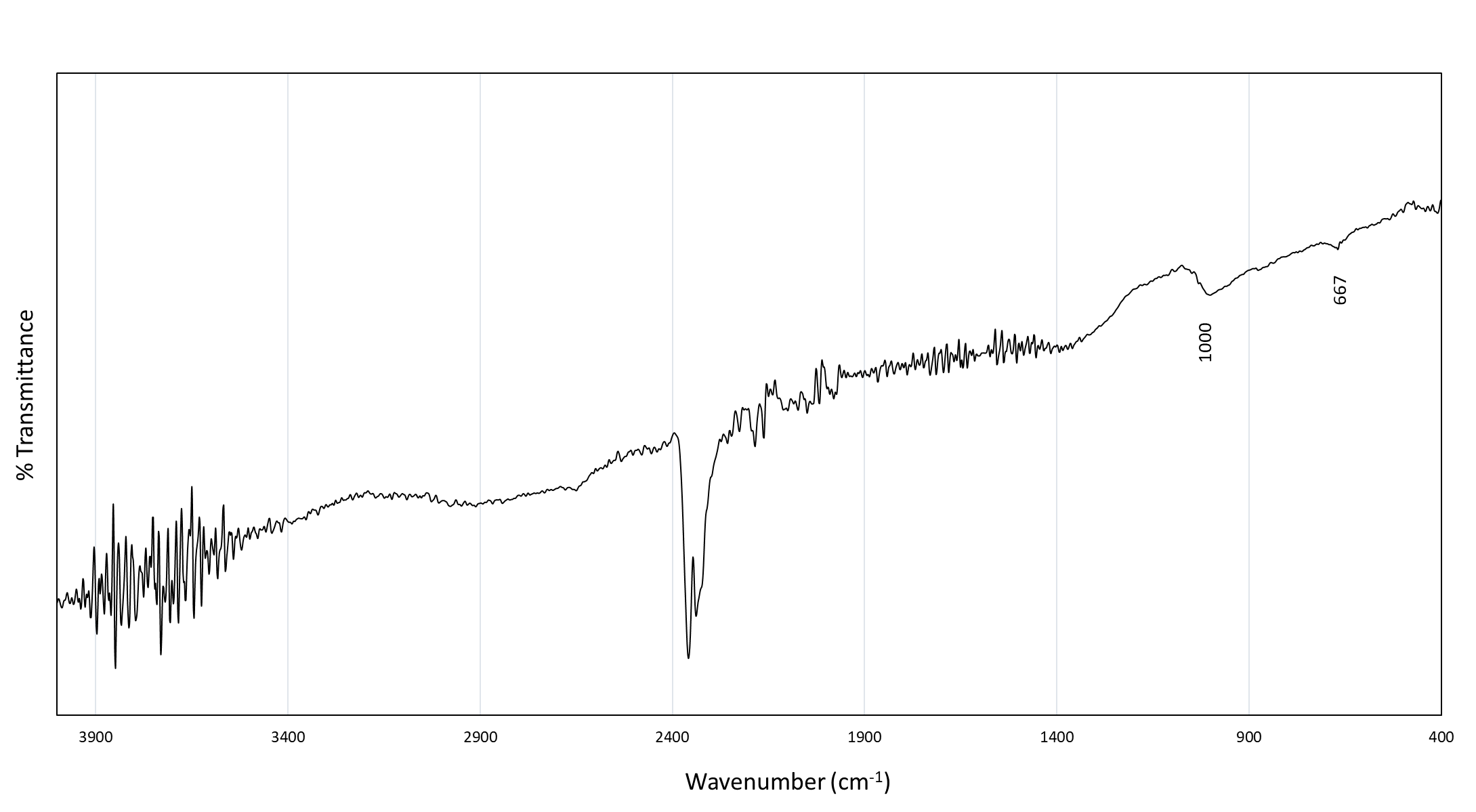
**

**Figure S13** FTIR spectrum of the sample from UC42810 displayed in percentage transmittance. The only identifiable peaks attributed to the sample were at 1000 cm^-1^ (cyclohexane bending or Si-O and Al-O stretching attributed to kaolin minerals[^25^](https://paperpile.com/c/29uT4b/UjEu)), while 667 cm^-1^ could not be attributed to a particular source, although it has been identified in gypsum[^18^](https://paperpile.com/c/29uT4b/1NCe).

**
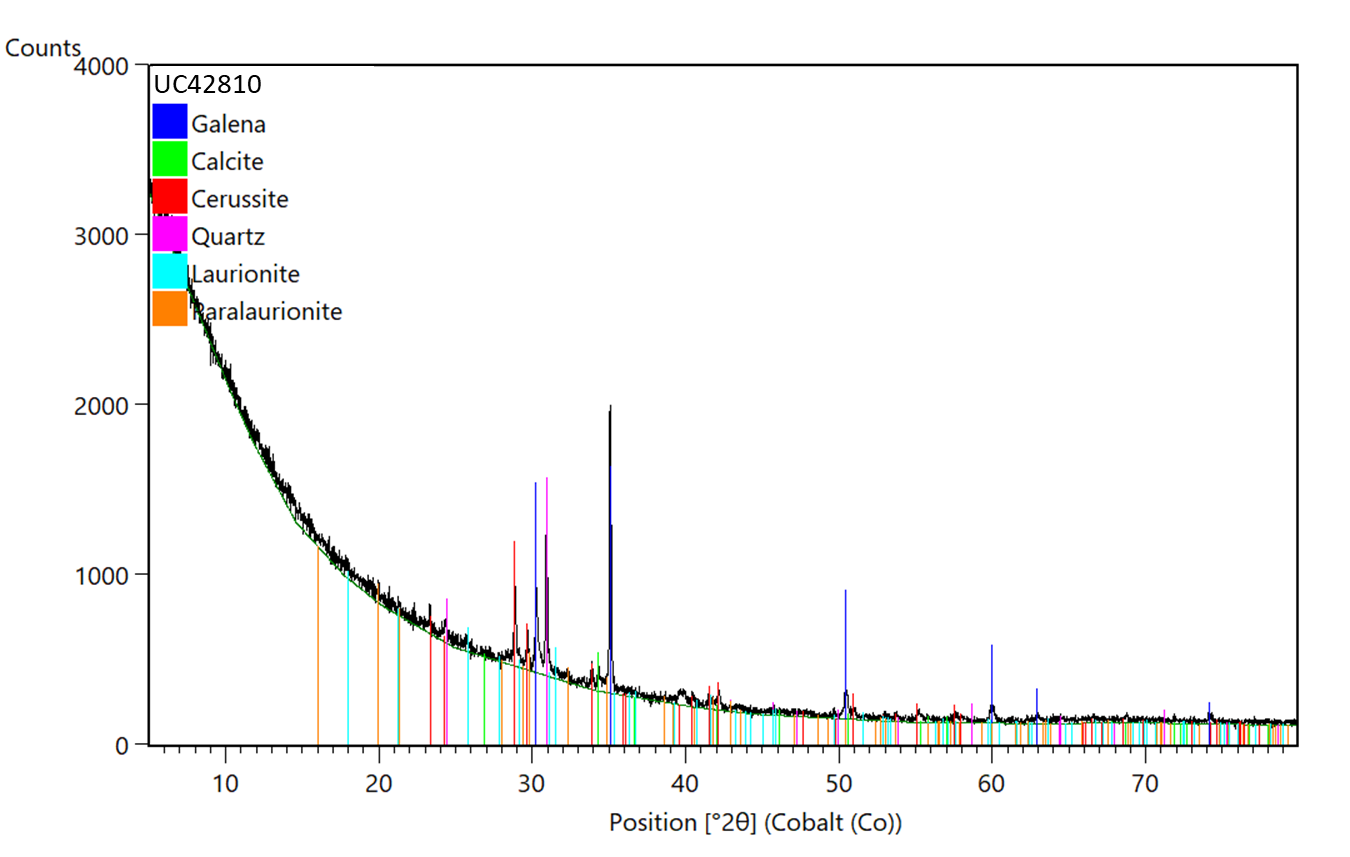
**

**Figure S14** X-ray diffractogram of the sample from UC42810. Galena, Calcite, Cerussite, Quartz, Laurionite and Paralaurionite were identified.


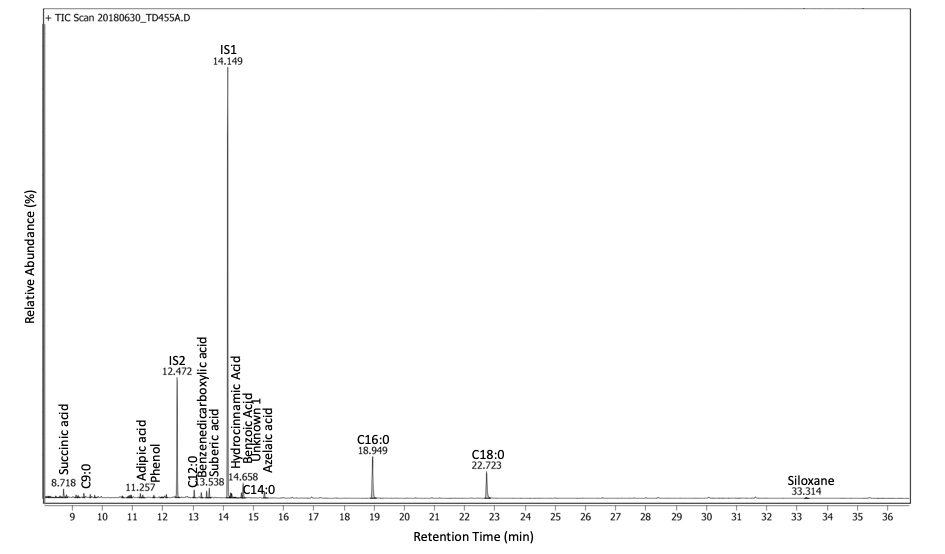


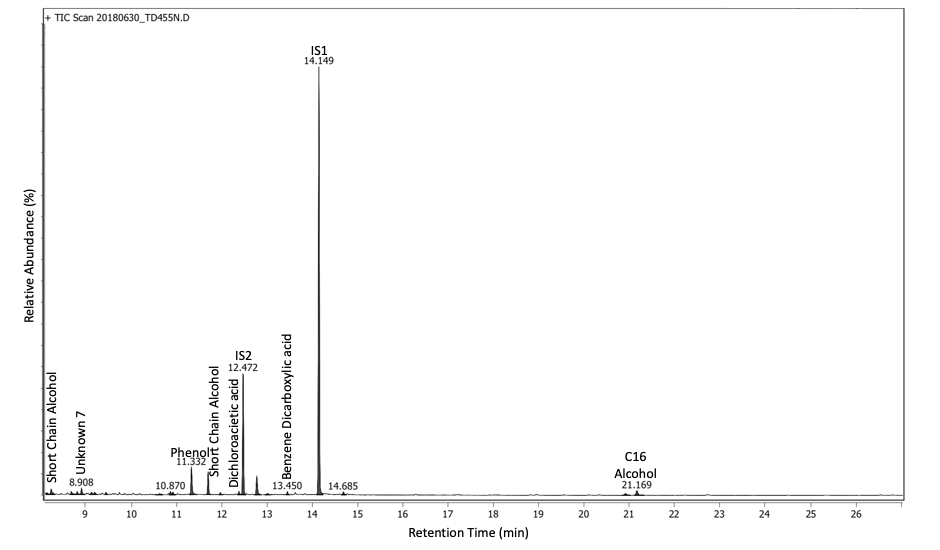


**Figure S15** GC/MS chromatograms obtained on the organic extract of the sample UC42810. Top and bottom chromatograms correspond to the acidic and neutral fractions, respectively. Peak identifications are reported in Table S4.

**Table S4** Identification of the compounds present in the organic fraction of the sample (UC42810). TMS indicates a trimethylsilyl ester.

| **Time (min)** | **Compound** | **Peak Area** |
| --- | --- | --- |
| *Acidic fraction* | | |
| 8.719 | butanedioic acid, TMS | 677621 |
| 9.387 | nonanoic acid, TMS | 187043 |
| 11.2593 | hexanedioic acid, TMS | 268456 |
| 12.4528 | heptanedioic acid, TMS | 314943 |
| 12.4772 | hexadecane (IS2) | 10401784 |
| 12.7521 | 4-benzoic acid, TMS | 46288 |
| 13.042 | dodecanoic acid, TMS | 572224 |
| 13.5389 | octanedioic acid, TMS | 678257 |
| 14.1499 | tridecanoic acid, TMS (IS1) | 39590724 |
| 14.2809 | vanillic acid, TMS | 260270 |
| 14.6611 | nonanedioic acid, TMS | 1234266 |
| 15.3651 | tetradecanoic acid, TMS | 642053 |
| 15.9964 | decanedioic acid, TMS | 39506 |
| 17.7298 | undecanedioic acid, TMS | 16519 |
| 18.9481 | hexadecanoic acid, TMS | 6721735 |
| 24.9914 | DHA, TMS | 35146 |
| 27.1909 | diisooctyl phthalate | 7456 |
| 27.9913 | 7-oxo-DHA, TMS | 35490 |
| 20.918 | heptadecanoic acid | 46538 |
| 16.9326 | pentadecanoic acid | 138660 |
| 20.389 | branched heptadecanoic acid | 33850 |
| 22.7247 | ocadecanoic acid, TMS | 4139086 |

**Table S4** Continued.

| **Time (min)** | **Compound** | **Peak area** |
| --- | --- | --- |
| *Neutral Fraction* | | |
| 8.148 | glycerol, TMS | 75017 |
| 12.4771 | hexadecane (IS2) | 10603797 |
| 14.1499 | tridecanoic acid, TMS (IS1) | 38983135 |
| 15.3637 | tetradecanoic acid, TMS | 17945 |
| 18.9542 | hexadecanoic acid, TMS | 101391 |
| 22.7103 | octadecanoic acid, TMS | 83660 |

**UC43078**


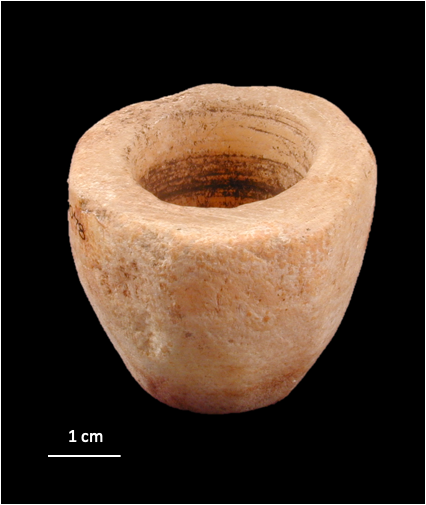


**Figure S16** Picture of a kohl pot UC43078 from Abydos, First Intermediate Period/Middle Kingdom (Dynasty XI), kept at the Petrie Museum. Object description: calcite kohl pot, wide crudely cut with angled shaping still evident, flat topped with circular cut interior, flat base, galena kohl remaining on interior. Sample description: black residue observed, sample taken on interior of neck of vessel. Image credit: Courtesy of the Petrie Museum of Egyptian Archaeology, UCL.


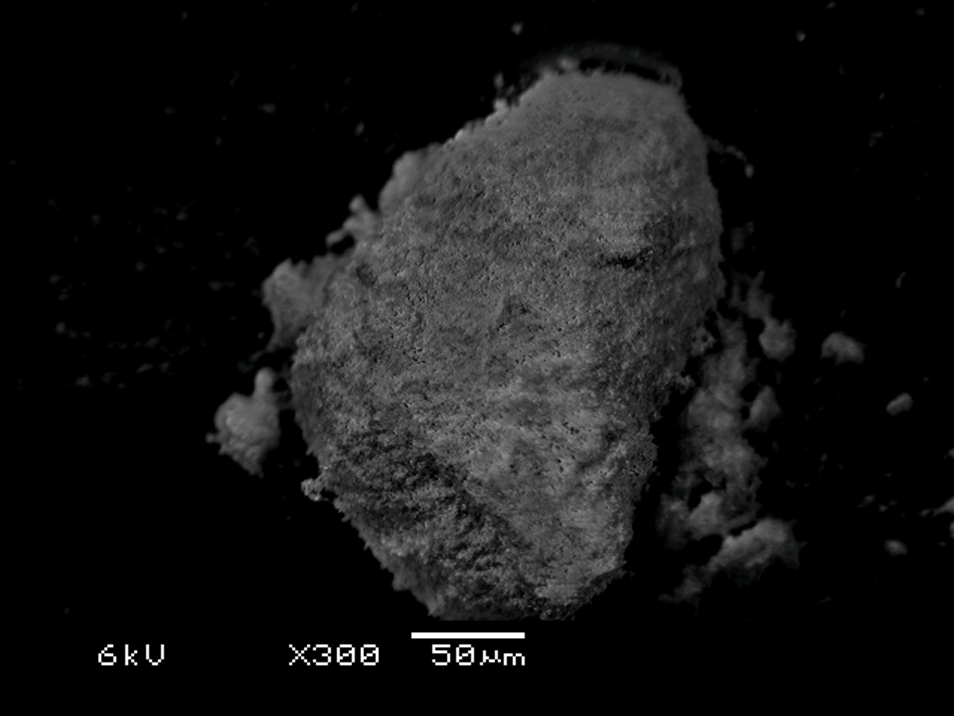


**Figure S17** SEM/EDS image of a microsample taken from the content of the object UC43078. Images were acquired between 3-5 kV, with a spot intensity of 53, in secondary electron detector mode (SED). Elemental composition: Major: O (56.37) Mn (30.14) C (10.02), Trace: Si (0.93) S (0.84) Ca (0.74) Al (0.33) Na (0.20) K (0.16) P (0.08).


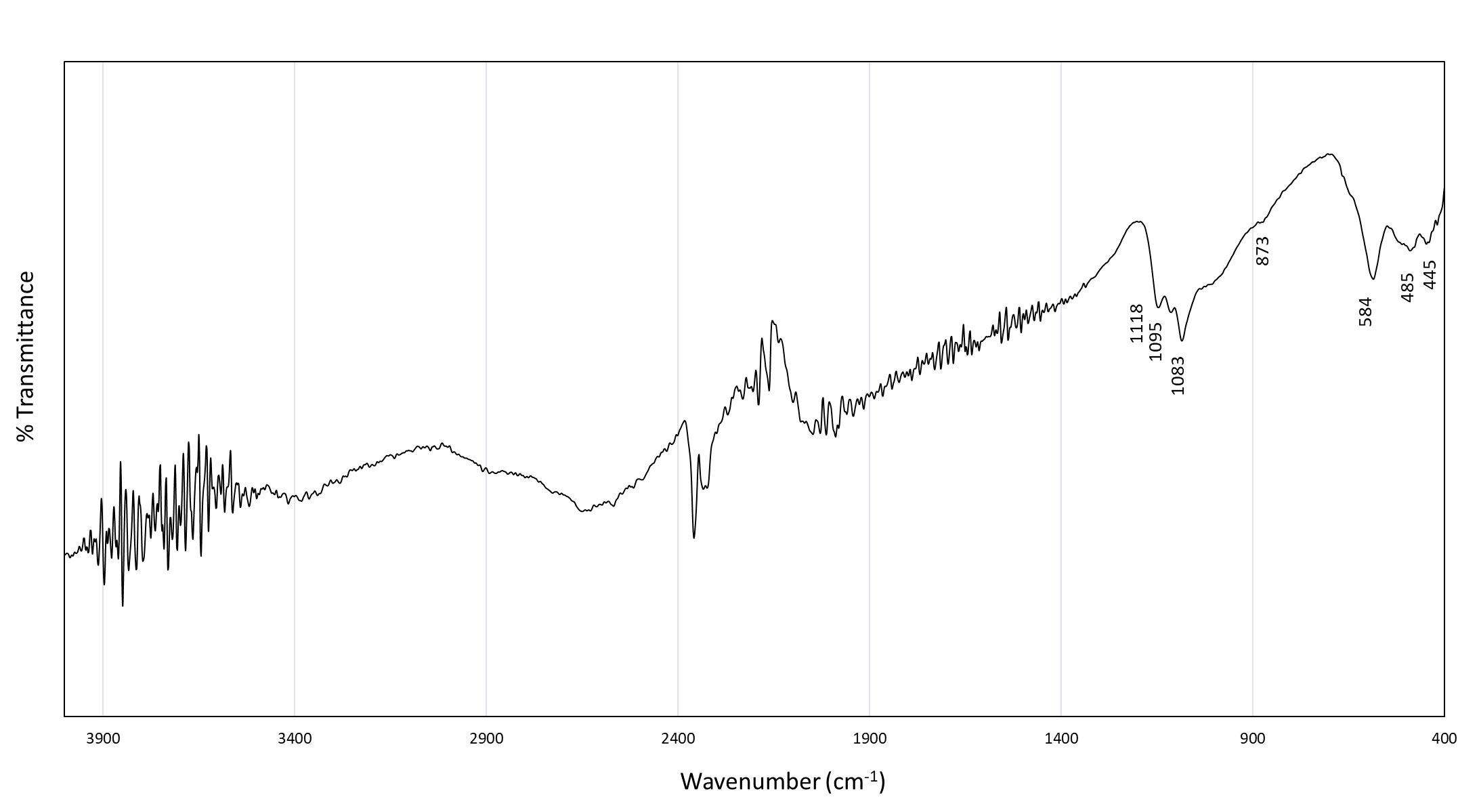


**Figure S18** FTIR spectrum of the sample from UC43078 displayed in percentage transmittance. Identifiable peaks span between 1200 to 400 cm^-1^, with a triplet around 1145 cm^-1^, 1112 cm^-1^ and 1083 cm^-1^, the latter of which may be attributed to Si-O or C-O vibrations[^21,25^](https://paperpile.com/c/29uT4b/UjEu+pfTm). The shoulder at 873 cm^-1^ has been found in carbonate minerals[^18^](https://paperpile.com/c/29uT4b/1NCe). The remaining peaks in the region between 1450 - 500 cm^-1^ could not be attributed to a particular mineral source


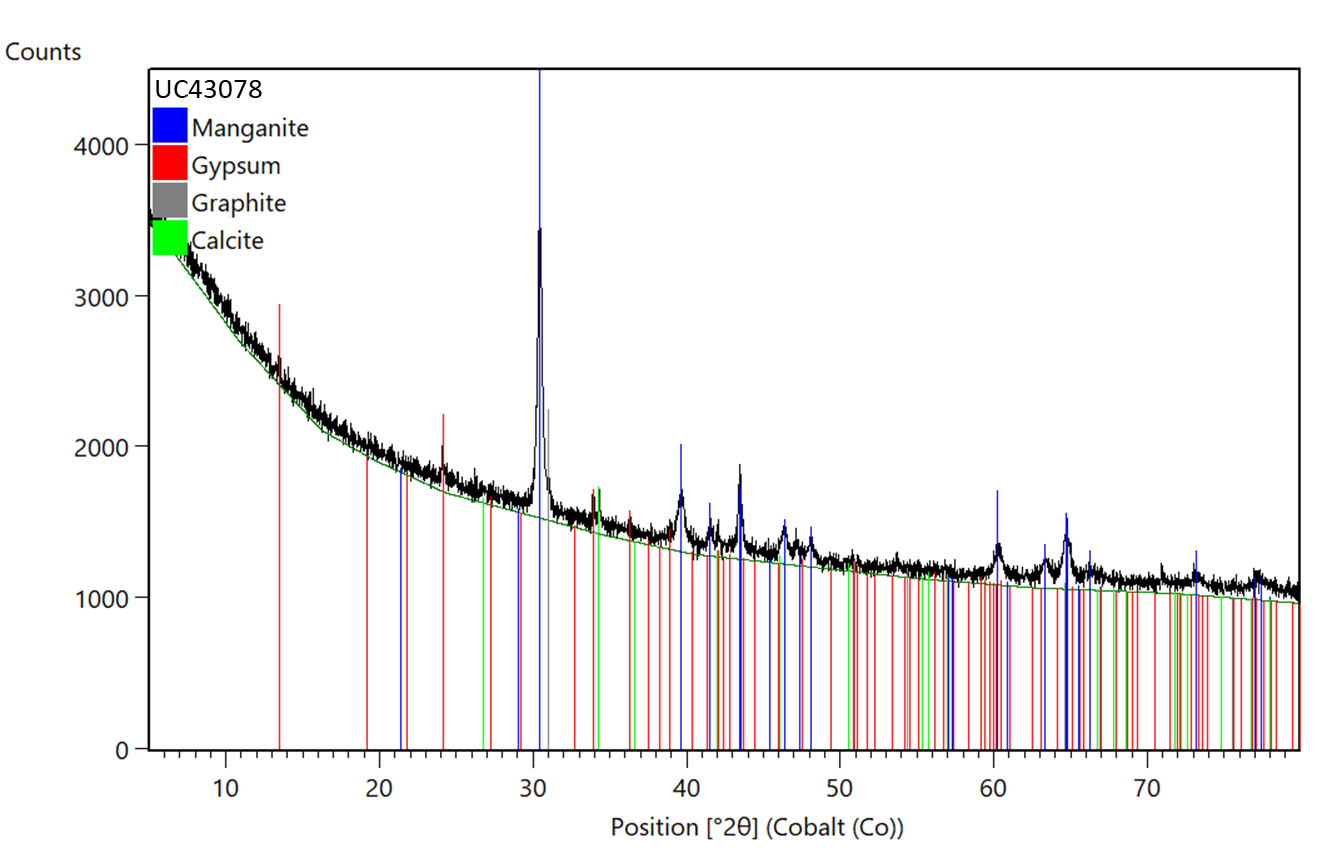


**Figure S19** X-ray diffractogram of the sample from UC43078. Manganite, Gypsum, and Calcite were identified.


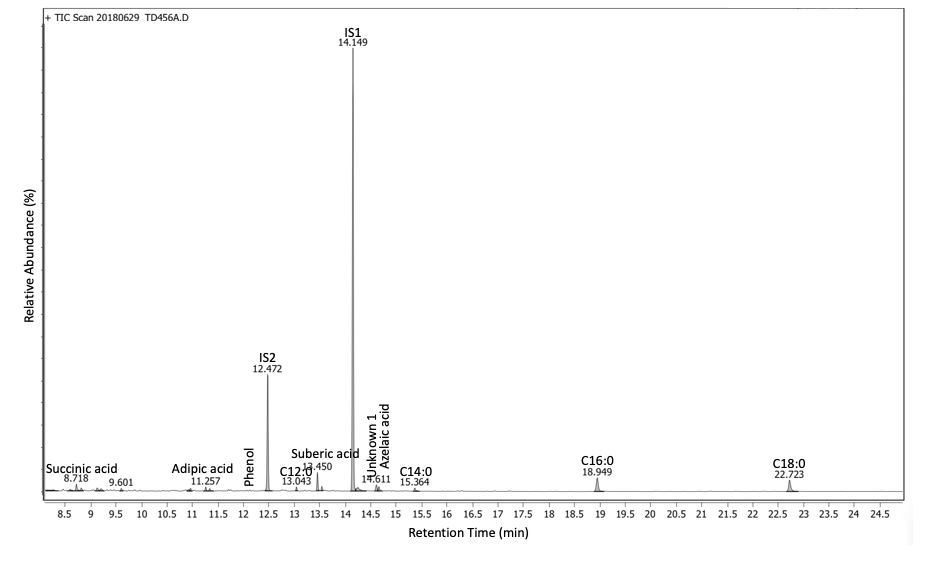

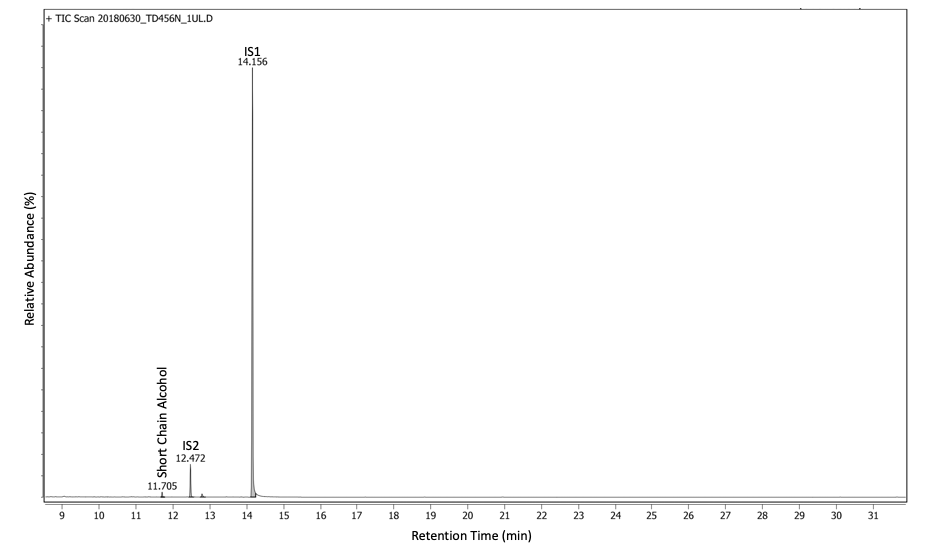


**Figure S20** GC/MS chromatograms obtained on the organic extract of the sample UC43078. Top and bottom chromatograms correspond to the acidic and neutral fractions, respectively. Peak identifications are reported in Table S5.

**Table S5** Identification of the compounds present in the organic fraction of the sample (UC43078). TMS indicates a trimethylsilyl ester.

| **Time (min)** | **Compound** | **Peak area** |
| --- | --- | --- |
| *Acidic fraction* | | |
| 8.7188 | butanedioic acid, TMS | 552140 |
| 11.2591 | hexanedioic acid, TMS | 264579 |
| 12.477 | hexadecane (IS2) | 10182784 |
| 12.752 | 4-benzoic acid, TMS | 53808 |
| 13.0412 | dodecanoic acid, TMS | 280589 |
| 13.5394 | octanedioic acid, TMS | 276305 |
| 14.1504 | tridecanoic acid, TMS (IS1) | 39206373 |
| 14.2821 | vanillic acid, TMS | 49490 |
| 14.6596 | nonanedioic acid, TMS | 294791 |
| 15.2649 | tetradecanoic acid, TMS | 292972 |
| 18.9486 | hexadecanoic acid, TMS | 2094789 |
| 20.911 | heptadecanoic acid, TMS | trace |
| 22.7258 | octadecanoic acid, TMS | 1838986 |
| 24.9953 | DHA, TMS | 48526 |

**UC43148**


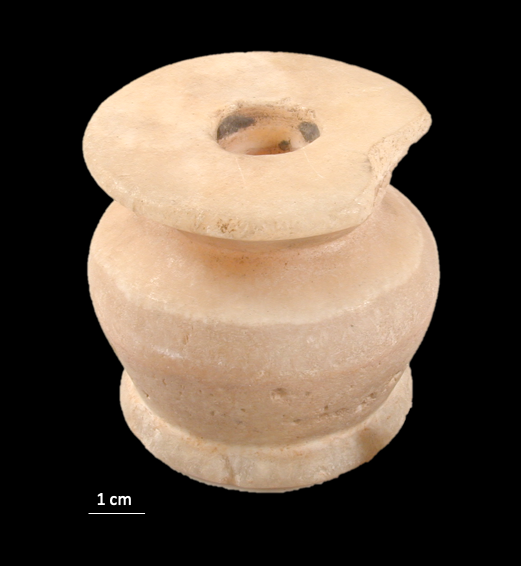


**Figure S21** Picture of a kohl pot UC43148 from Abydos, Middle Kingdom (Dynasty XII), kept at the Petrie Museum. Object description: calcite kohl pot, flat topped rim, constricted neck, rounded shoulder, body tapered to flat base with extended rim, contents in interior. Sample description: black residue observed, sample taken from the interior of the neck, opposite the break in the vessel. Image credit: Courtesy of the Petrie Museum of Egyptian Archaeology, UCL.


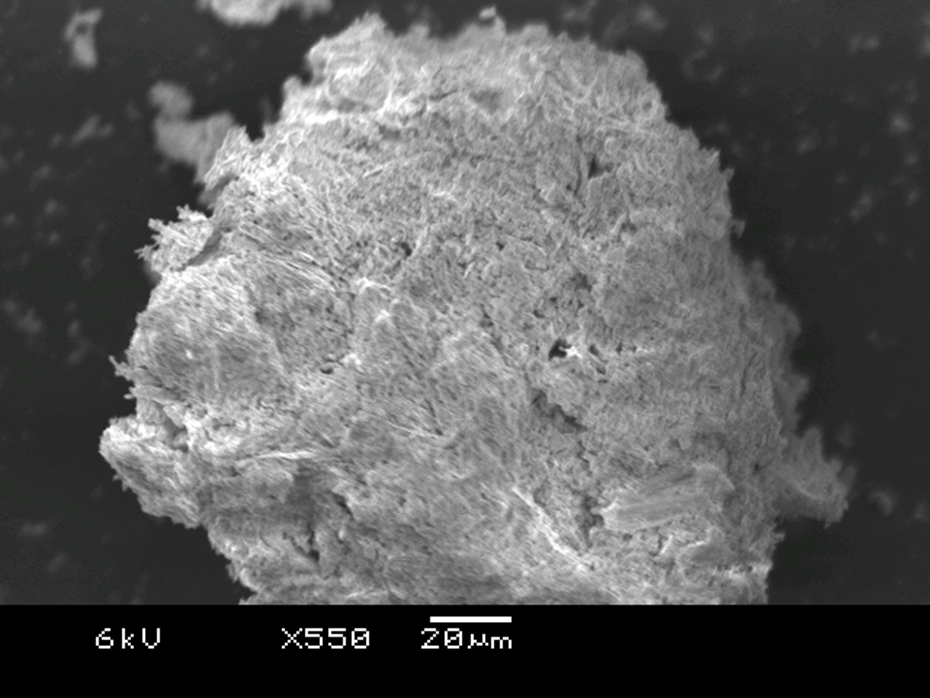


**Figure S22** SEM/EDS image of a microsample taken from the content of the object UC43148. Images were acquired between 3-5 kV, with a spot intensity of 53, in secondary electron detector mode (SED). Elemental Composition: Major: O (60.27) C (18.56) Mn (16.51); Minor: Si (1.15); Trace: Al (0.76) Na (0.70) Ca (0.42) S (0.40) Pb (0.29) Cl (0.28) P (0.23) Mg (0.21) Cu (0.12) K (0.11).


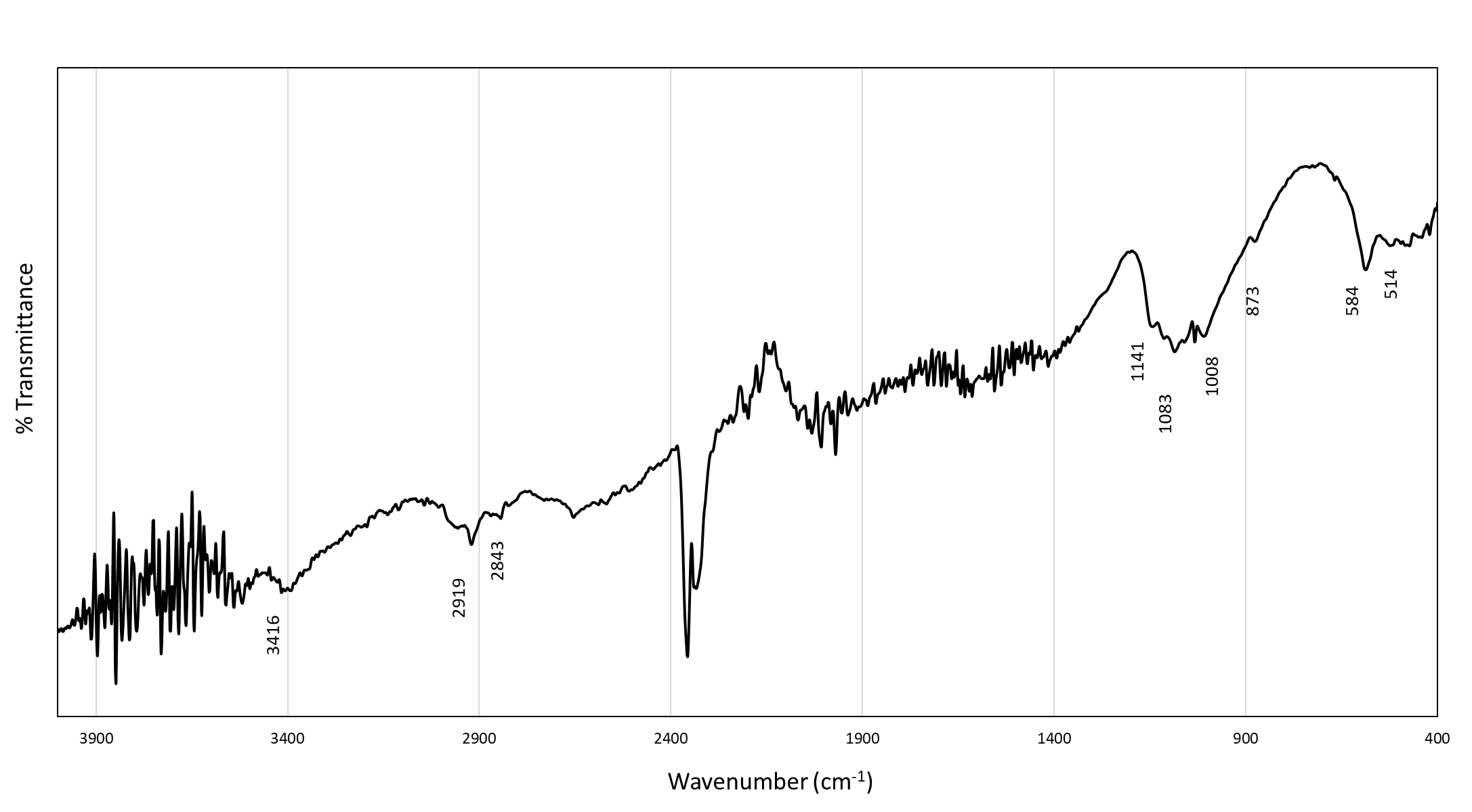


**Figure S23** FTIR spectrum of the sample from UC43148 displayed in percentage transmittance. This spectrum yielded a very weak -OH stretching peak at 3416 cm^-1^ alongside two weak C-H stretching peaks at 2919 and 2843 cm^-1^, respectively. Similar to UC43078, a triplicate at 1141 cm^-1^, 1112 cm^-1^, and 1083 cm^-1^ was observed, in addition to a peak at 1008 cm^-1^. The latter two peaks may be attributed to Si-O or C-O vibrations and Al-O/Si-O vibrations, respectively[^21,25^](https://paperpile.com/c/29uT4b/pfTm+UjEu). The shoulder at 873 cm^-1^ has been found in carbonate minerals[^18^](https://paperpile.com/c/29uT4b/1NCe). While there are similarities in peak locations at 854 cm^-1^ and 514 cm^-1^ an open-source IR reference for sand[^18^](https://paperpile.com/c/29uT4b/1NCe), however, the main markers for silicates (doublet at 794 and 777, alongside a strong 694 peak) are notably absent in this specimen.


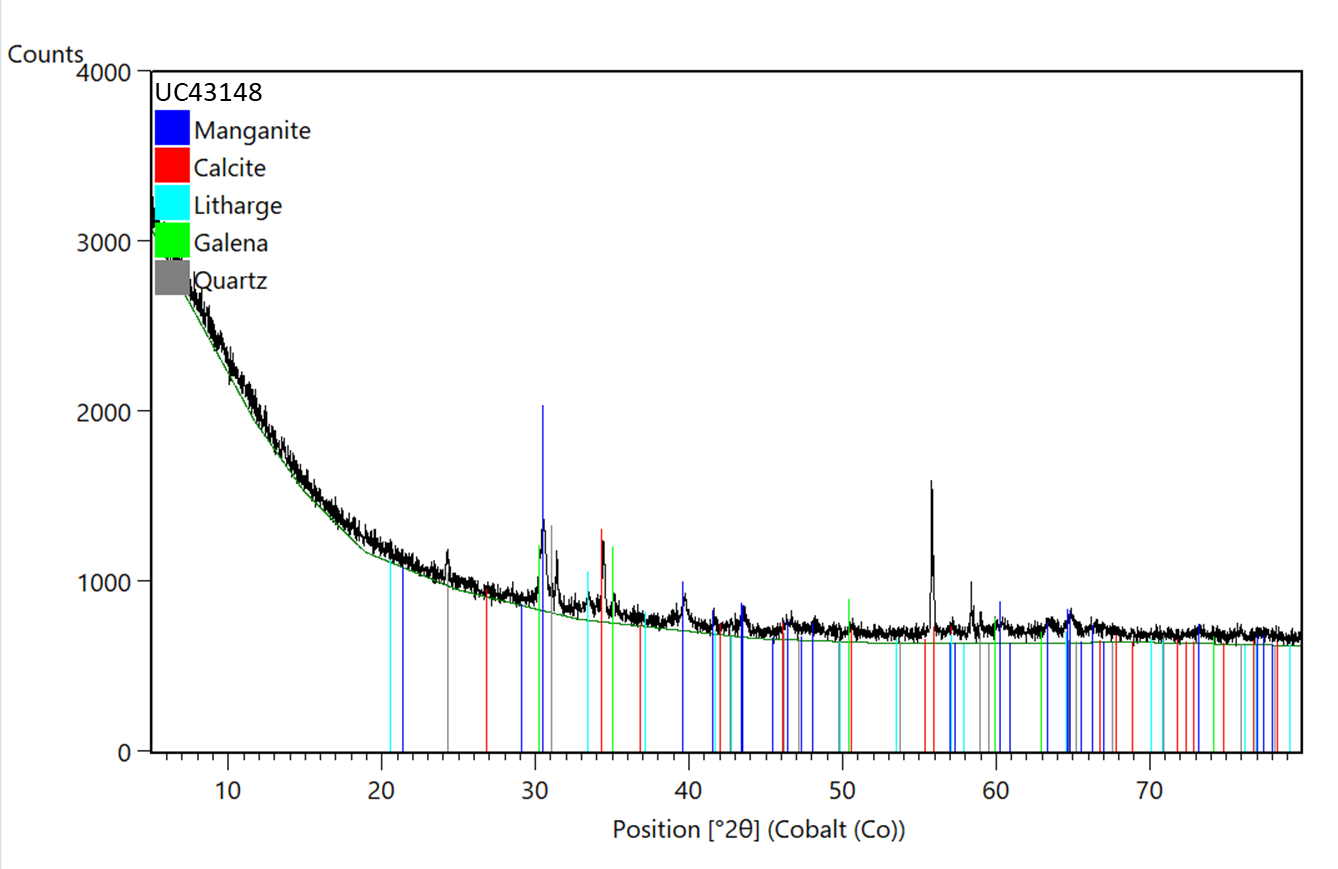


**Figure S24** X-ray diffractogram of the sample from UC43148. Manganite, Calcite, Litharge, Galena and Quartz were identified.


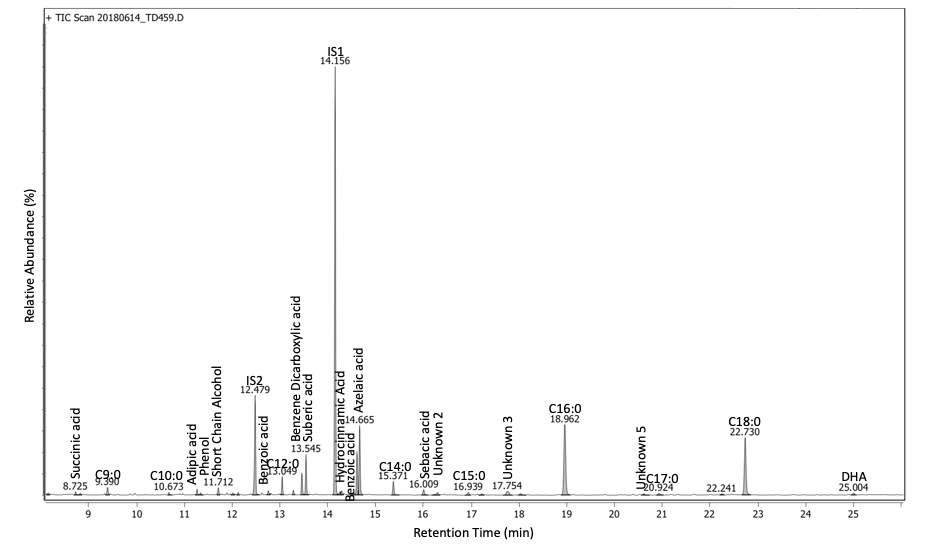

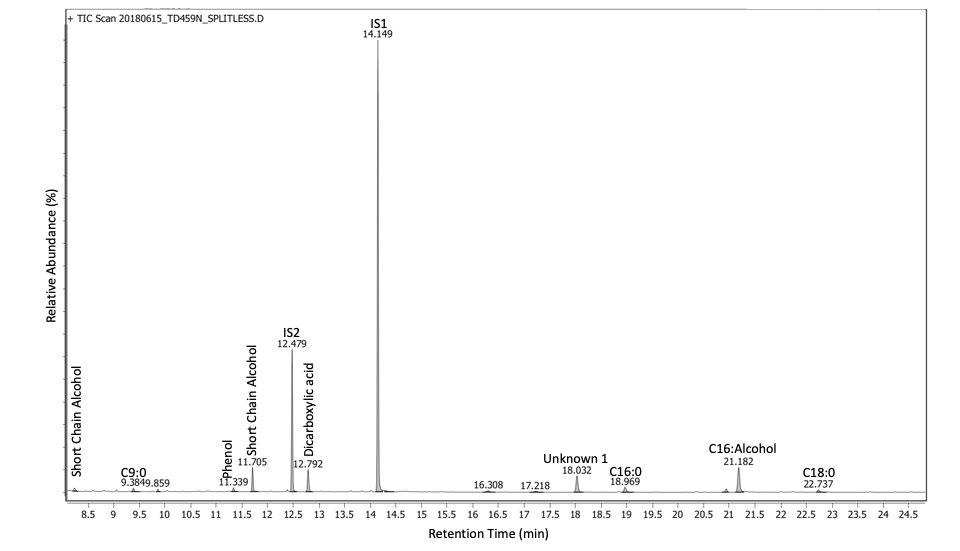
**Figure S25** GC/MS chromatograms obtained on the organic extract of the sample UC43148. Top and bottom chromatograms correspond to the acidic and neutral fractions, respectively. Peak identifications are reported in Table S6.

**Table S6** Identification of the compounds present in the organic fraction of the sample UC43148. TMS indicates a trimethylsilyl ester.

| **Time (min)** | **Compound** | **Peak area** |
| --- | --- | --- |
| *Acidic fraction* | | |
| 8.1548 | glycerol, TMS | 97847 |
| 8.7257 | butanedioic acid, TMS | 224812 |
| 9.3917 | nonanoic acid, TMS | 587075 |
| 11.2653 | hexanedioic acid, TMS | 337557 |
| 12.4595 | heptanedioic acid, TMS | 921668 |
| 12.4832 | hexadecane (IS2) | 8423773 |
| 12.7575 | 4-benzoic acid, TMS | 261538 |
| 13.048 | dodecanoic acid, TMS | 1284818 |
| 13.5456 | octanedioic acid, TMS | 2787622 |
| 14.1553 | tridecanoic acid, TMS (IS1) | 35330873 |
| 14.2904 | vanillic acid, TMS | 227637 |
| 14.6684 | nonanedioic acid, TMS | 5953628 |
| 15.3759 | tetradecanoic acid, TMS | 1231800 |
| 16.0086 | decanedioic acid, TMS | 495466 |
| 16.44 | branched pentadecanoic acid | 52434 |
| 16.94 | pentadecanoic acid | 295389 |
| 18.9575 | hexadecanoic acid, TMS | 10385053 |
| 19.8027 | dodecanedioic acid, TMS | 53684 |
| 20.2168 | branched heptadecanoic acid | 31972 |
| 20.388 | branched heptadecanoic acid | 92507 |
| 20.924 | heptadecanoic acid, TMS | 164925 |
| 22.7307 | octadecanoic acid, TMS | 7575585 |

**Table S6** Continued.

| **Time (min)** | **Compound** | **Peak area** |
| --- | --- | --- |
| 25.0049 | DHA, TMS | 208990 |
| 27.1956 | diisooctyl phthalate | 42001 |
| *Neutral Fraction* | | |
| 8.1454 | glycerol, TMS | 26221 |
| 9.3849 | nonanoic acid, TMS | 107425 |
| 12.4779 | hexadecane (IS2) | 5284860 |
| 13.0461 | dodecanoic acid, TMS | 7584 |
| 14.1492 | tridecanoic acid, TMS (IS1) | 16104674 |
| 18.9685 | hexadecanoic acid, TMS | 18.9685 |
| 22.7362 | octadecanoic acid, TMS | 77945 |
| 26.81 | n-alkane, C25 |  |
| 27.1943 | diisooctyl phthalate | 116009 |
| 28.202 | n-alkane, C26 |  |
| 29.505 | n-alkane, C27 |  |

**UC64751**


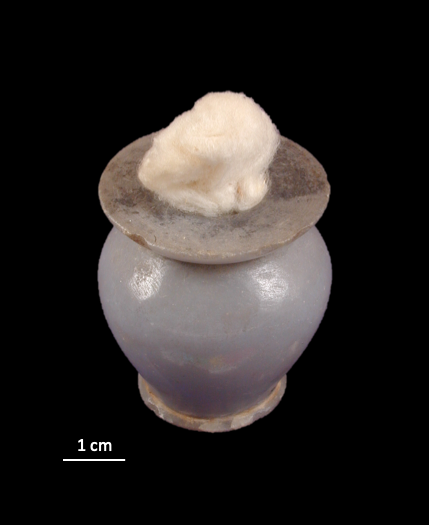


**Figure S26** Picture of a kohl pot UC64751 of unknown provenance, Middle Kingdom (Dynasty XII), kept at the Petrie Museum. Object description: Anhydrite kohl pot, tall body, broad flat rim, narrow flat base; rim and base slightly abraded; modern cotton wool in mouth to keep residue inside; formerly in the collection of Grenfell, then in that of Henry Wellcome. Sample description: sample taken from the loose powder present inside the vessel after removal of the modern cotton wool by a conservator of the museum. Image credit: Courtesy of the Petrie Museum of Egyptian Archaeology, UCL.


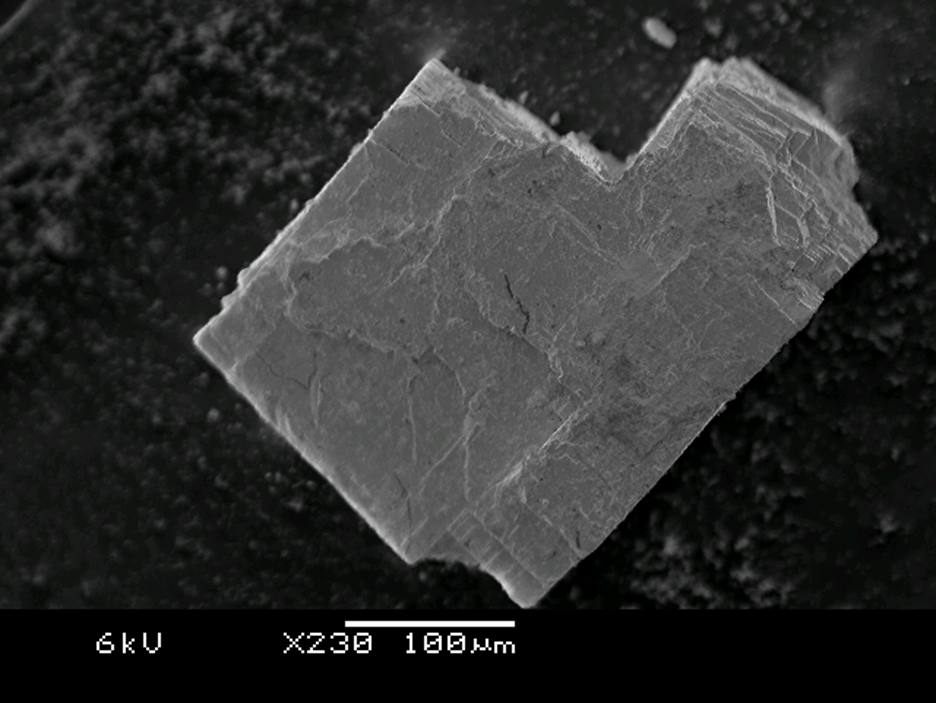


**Figure S27** SEM/EDS image of a microsample taken from the content of the object UC64751. Images were acquired between 3-5 kV, with a spot intensity of 53, in secondary electron detector mode (SED). Elemental Composition: Major: Pb (38.77) S (38.16) C (11.94); Minor: O (6.61) Cl (4.52).


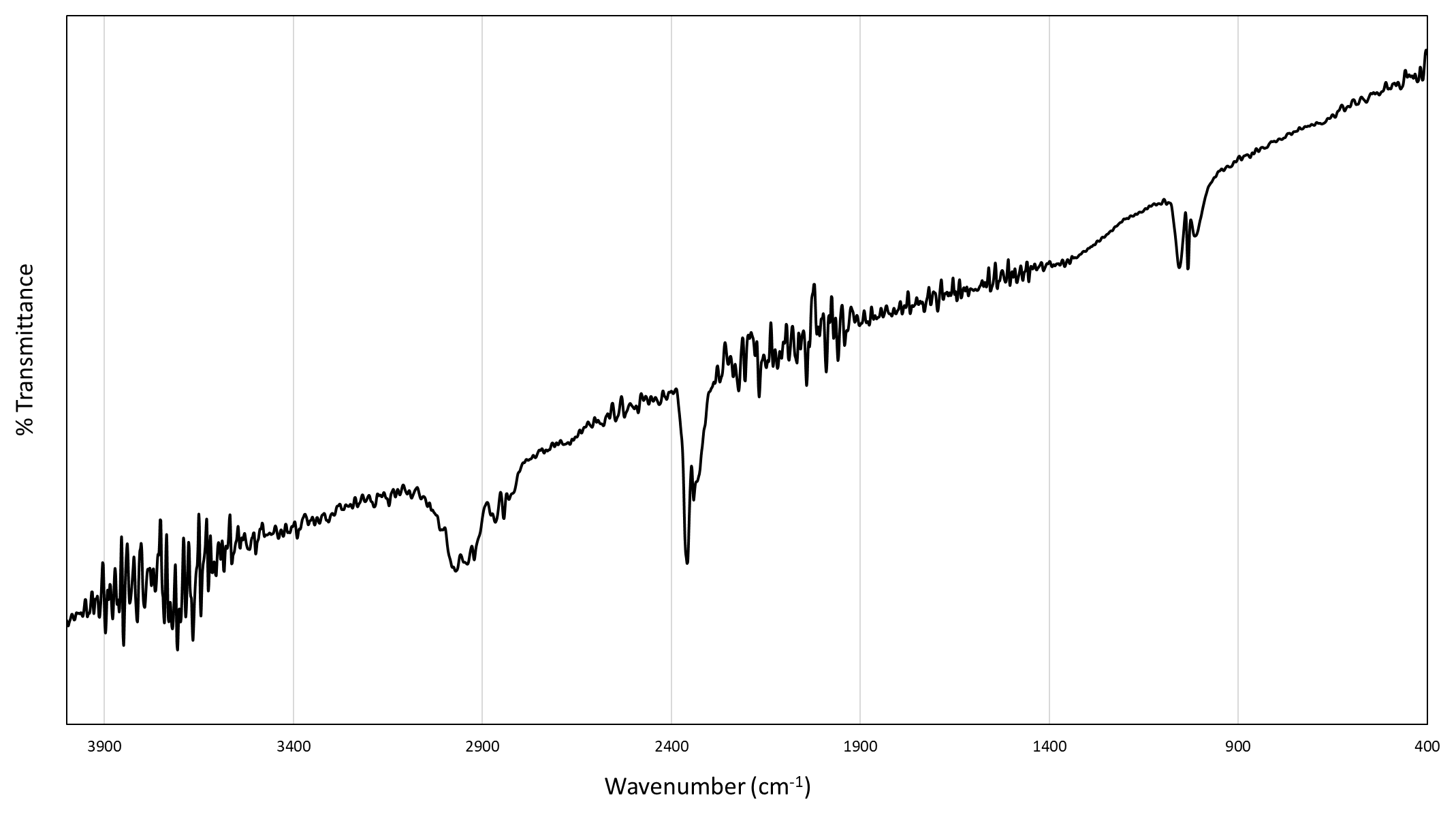


**Figure S28** FTIR spectrum of the sample from UC64751 displayed in percentage transmittance. No peaks were identifiable in this spectrum.


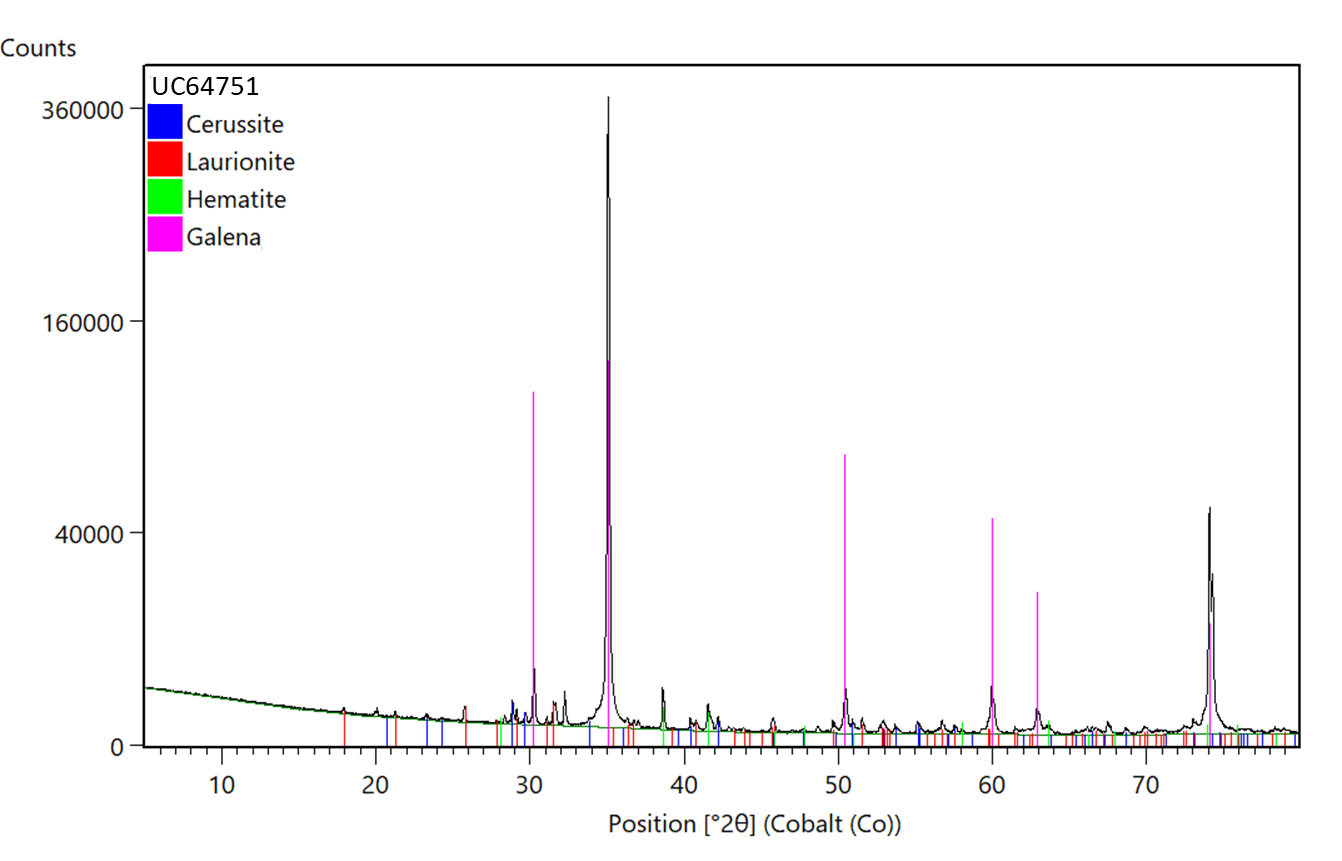


**Figure S29** X-ray diffractogram of the sample from UC64751. Cerussite, Laurionite, Hematite and Galena were identified.


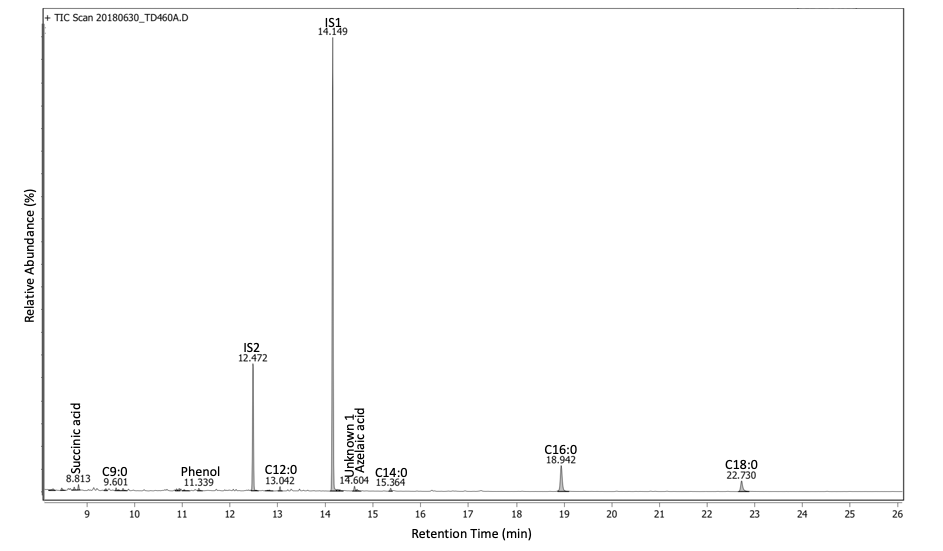

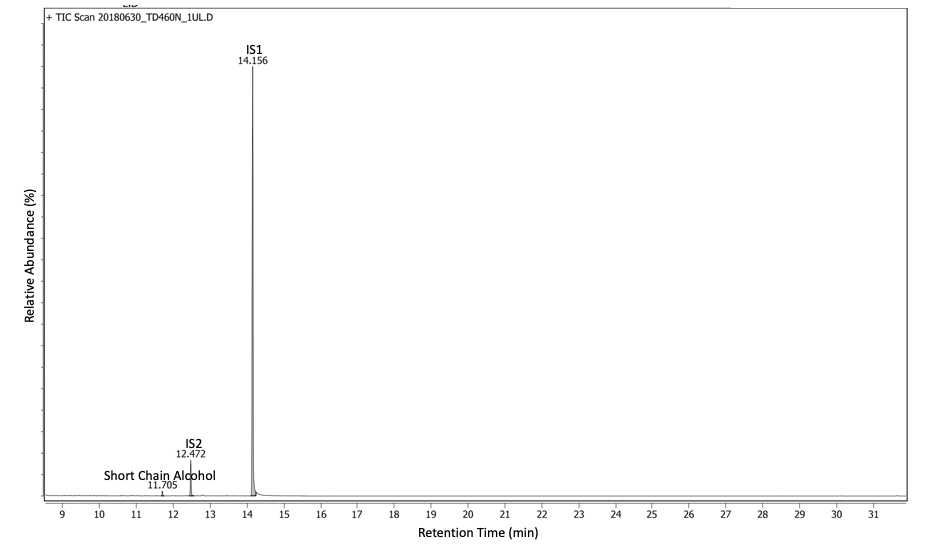


**Figure S30** GC/MS chromatograms obtained on the organic extract of the sample UC64751. Top and bottom chromatograms correspond to the acidic and neutral fractions, respectively. Peak identifications are reported in Table S7.

**Table S7** Identification of the compounds present in the organic fraction of the sample UC64751. TMS indicates a trimethylsilyl ester.

| **Time (min)** | **Compound** | **Peak area** |
| --- | --- | --- |
| *Acidic fraction* | | |
| 8.7228 | butanedioic acid, TMS | 208940 |
| 9.3875 | nonanoic acid, TMS | 76889 |
| 11.2618 | hexanedioic acid, TMS | 46097 |
| 12.4769 | hexadecane (IS2) | 10778824 |
| 13.0424 | dodecanoic acid, TMS | 276374 |
| 13.5373 | octanedioic acid, TMS | 27819 |
| 14.149 | tridecanoic acid, TMS (IS1) | 39092422 |
| 14.6581 | nonanedioic acid, TMS | 138626 |
| 15.3642 | tetradecanoic acid, TMS | 276205 |
| 18.9465 | hexadecanoic acid, TMS | 3784241 |
| 22.7332 | octadecanoic acid, TMS | 1803532 |
| *Neutral Fraction* | | |
| 12.475 | IS2 | 4246331 |
| 14.1552 | IS1 | 64738297 |

**UC46348**


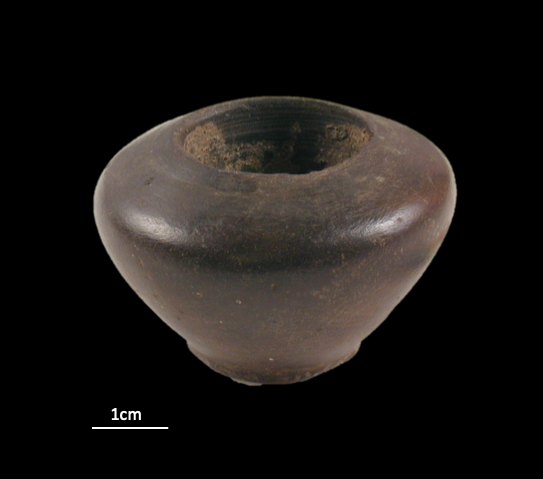


**Figure S31** Picture of a kohl pot UC46348 from Amarna, Middle Kingdom, kept at the Petrie museum. Object description: Dark brown stone (mafic) vessel: kohl pot. No rim. Gently sloping shoulder. Flat circular base. The top of the neck flares outward to meet the flat rim. Ridges on edge of rim. Concentric grooves and adhering matter on the interior wall of the vessel. Sample description: Flake of residue taken from inside the vessel. Image credit: Courtesy of the Petrie Museum of Egyptian Archaeology, UCL.


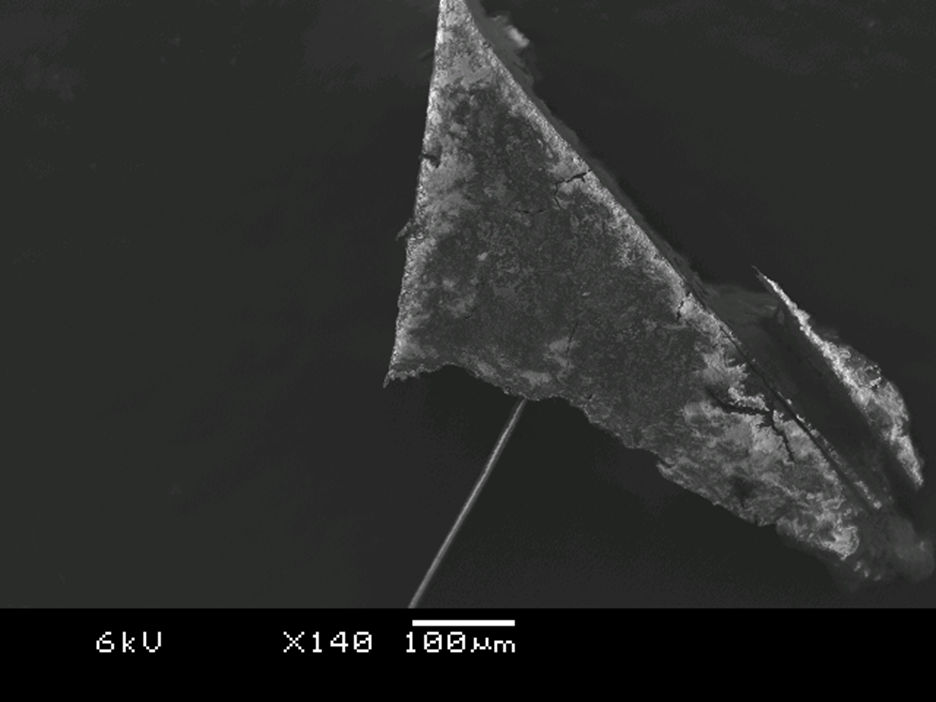


**Figure S32** SEM/EDS image of a microsample taken from the content of the object UC46348. Images were acquired between 3-5 kV, with a spot intensity of 53, in secondary electron detector mode (SED). Elemental Composition: Major: O (66.24) C (31.29); Trace: Si (0.78) Mg (0.45) Al (0.36) Na (0.25) S (0.2) Ca (0.14) Fe (0.13) K (0.05) Cl (0.05) Zn (0.03) P (0.02).


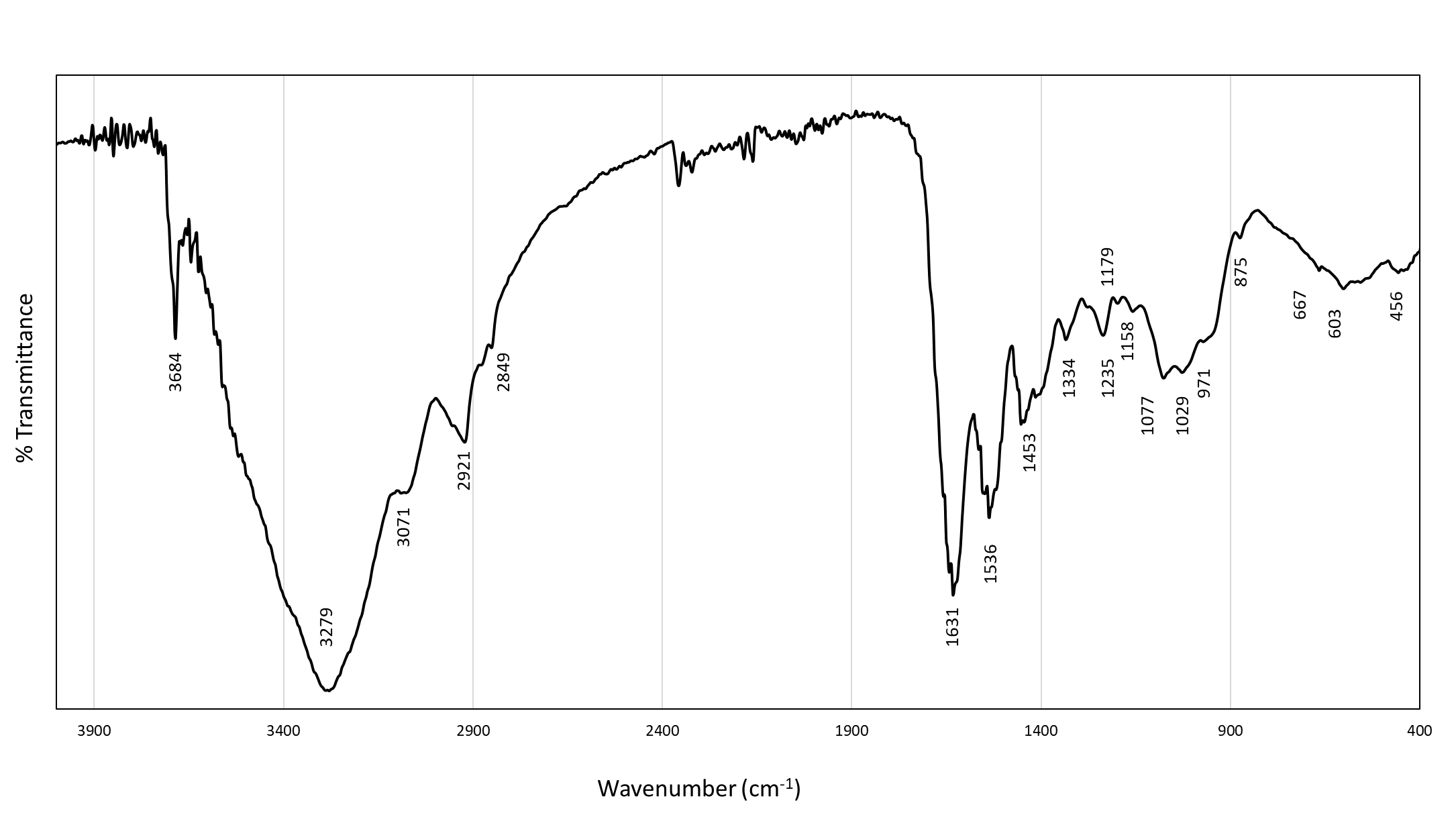


**Figure S33** FTIR spectrum of the sample from UC46348 displayed in percentage transmittance. The spectrum is consistent with a combination of organic and inorganic materials. The presence of a kaolinite group mineral may be interpreted based on characteristic -OH stretching peaks at 3684 cm^-1^ (dominant), and weaker peaks at 3644 cm^-1^ and 3622 cm^-1 [^[^18,25^](https://paperpile.com/c/29uT4b/1NCe+UjEu)^]^. A broad peak at 3279 cm^-1^ may be attributed to an -OH stretching and amide A region, while a weaker peak at 3071 may belong to Amide B. Weaker C-H stretches of aliphatics are identified 2921 cm^-1^ and 2849 cm^-1^. Peaks and assigned attributed from 1700 cm^-1^ included: 1633 cm^-1^ (amide I), 1563 cm^-1^ (COO anion), 1453 cm^-1^ (C-H deformation of CH_2_ and CH_3_), 1415 cm^-1^ (carbonates), 1334 cm^-1^ (aliphatic C-H), 1235 cm^-1^ (amide III), 1179 cm^-1^ (SiO4), 1029 cm^-1^ (PO4), 875 cm^-1^ (carbonates)[^18,22,23^](https://paperpile.com/c/29uT4b/1NCe+GKPQ+KFYJ). Additional peaks at 1077 cm^-1^ and 971 cm^-1^ may be attributed to carbohydrate moieties[^22^](https://paperpile.com/c/29uT4b/KFYJ).


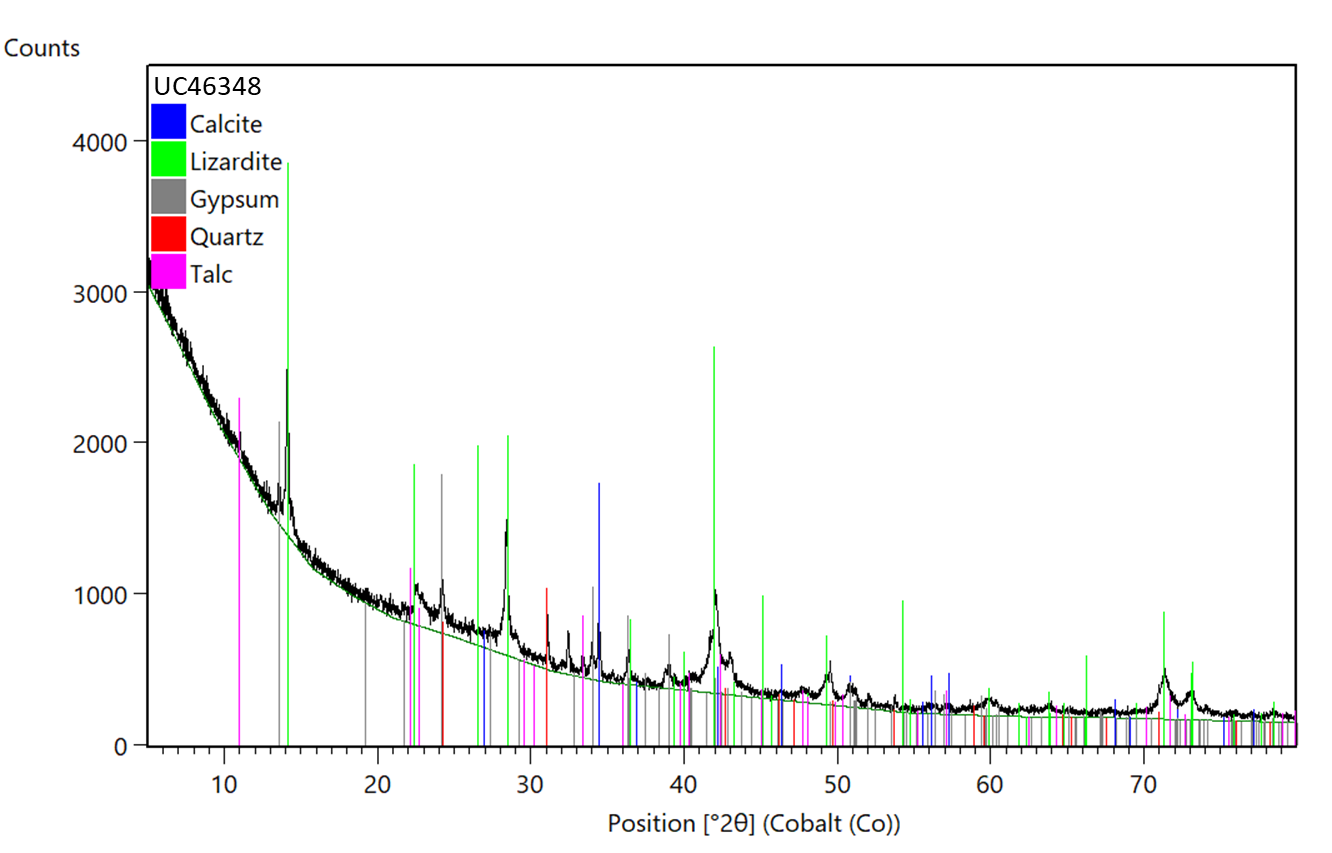


**Figure S34** X-ray diffractogram of the sample from UC46348. Calcite, Lizardite, Gypsum, Quartz and Talc were identified.


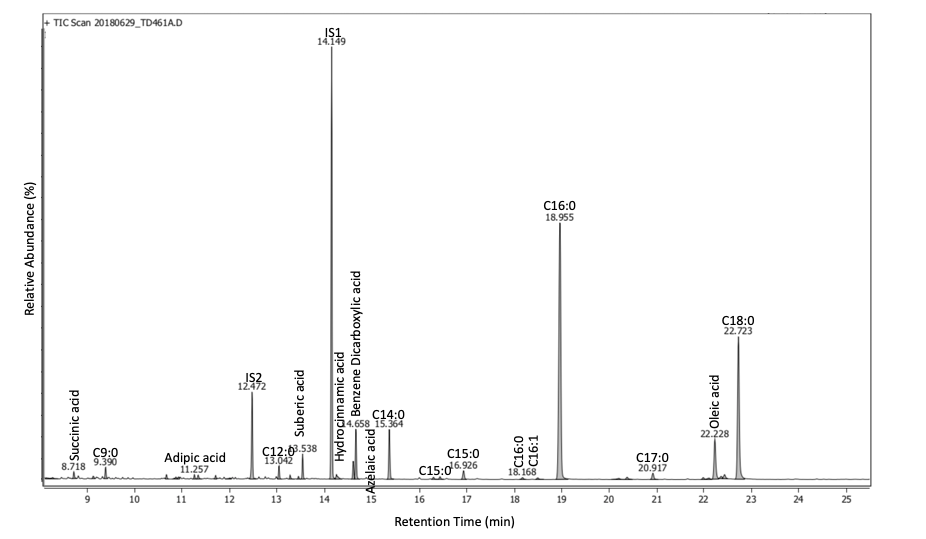

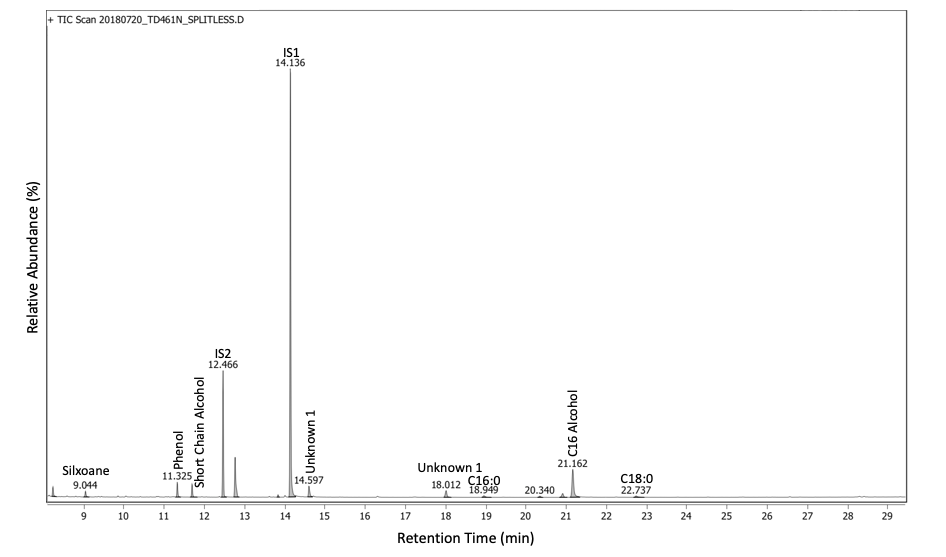


**Figure S35** GC/MS chromatograms obtained on the organic extract of the sample UC46348. Top and bottom chromatograms correspond to the acidic and neutral fractions, respectively. Peak identifications are reported in Table S8.

**Table S8** Identification of the compounds present in the organic fraction of the sample from UC46348. TMS indicates a trimethylsilyl ester.

| **Time (min)** | **Compound** | **Peak area** |
| --- | --- | --- |
| *Acidic fraction* | | |
| 8.7215 | butanedioic acid, TMS | 563409 |
| 9.3888 | nonanoic acid, TMS | 973037 |
| 11.2598 | hexanedioic acid, TMS | 330477 |
| 11.885 | undecanoic acid, TMS | 65208 |
| 12.4532 | heptanedioic acid, TMS | 527296 |
| 12.4763 | hexadecane (IS2) | 7560674 |
| 12.7519 | 4-benzoic acid, TMS | 115066 |
| 13.0418 | dodecanoic acid, TMS | 979761 |
| 13.5387 | octanedioic acid, TMS | 1834487 |
| 14.1497 | tridecanoic acid, TMS (IS1) | 38111793 |
| 14.278 | vanillic acid, TMS | 122763 |
| 14.6616 | nonanedioic acid, TMS | 4387130 |
| 15.3656 | tetradecanoic acid, TMS | 4785421 |
| 15.9962 | decanedioic acid, TMS | 119539 |
| 16.2936 | branched pentadecanoic acid | 70906 |
| 16.43 | branched pentadecanoic acid | 214411 |
| 16.905 | pentadecanoic acid, TMS | 1056335 |
| 17.7341 | undecanedioic acid, TMS | 39338 |
| 18.4931 | palmitoleic acid, TMS | 180058 |
| 18.9493 | hexadecanoic acid, TMS | 41843426 |
| 20.1977 | branched heptadecanoic acid | 117960 |
| 20.3681 | branched heptadecanoic acid | 150329 |

**Table S8** Continued.

| **Time (min)** | **Compound** | **Peak area** |
| --- | --- | --- |
| 20.917 | heptadecanoic acid, TMS | 856499 |
| 22.2282 | octadecenoic acid, TMS | 5750387 |
| 22.431 | octadecenoic acid, TMS | 577145 |
| 22.7244 | octadecanoic acid, TMS | 21088981 |
| 23.9247 | nonadecanoic acid, TMS | 45481 |
| 25.9049 | eicosanoic acid, TMS | 74129 |
| 28.6706 | docasanoic acid, TMS | 29282 |
| *Neutral Fraction* | | |
| 12.4656 | hexadecane (IS2) | 5752793 |
| 14.1397 | tridecanoic acid, TMS (IS1) | 22118923 |
| 18.9426 | hexadecanoic acid, TMS | 165224 |
| 22.7375 | octadecanoic acid, TMS | 134365 |

**UC7321**


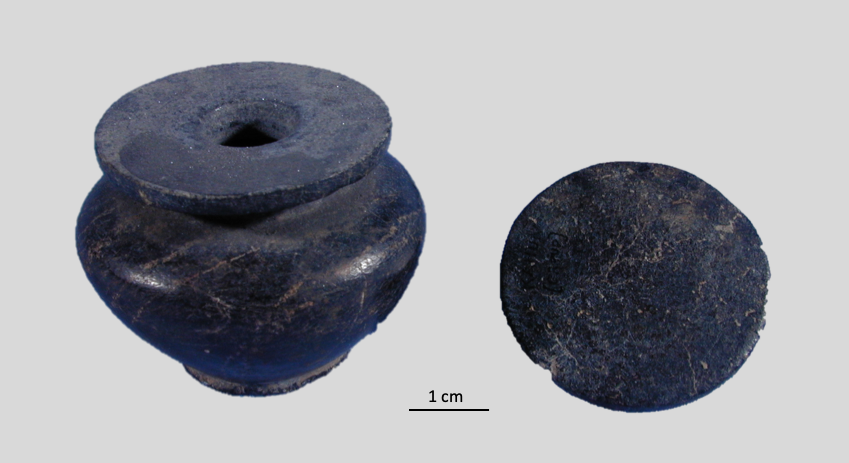


**Figure S36** Picture of a kohl pot UC7321 from Lahun, Late Middle Kingdom, kept at the Petrie Museum. Object description: Black serpentine kohl pot, with lid. Containing powdered kohl. Sample description: Loose powder from inside the vessel. Image credit: Courtesy of the Petrie Museum of Egyptian Archaeology, UCL.


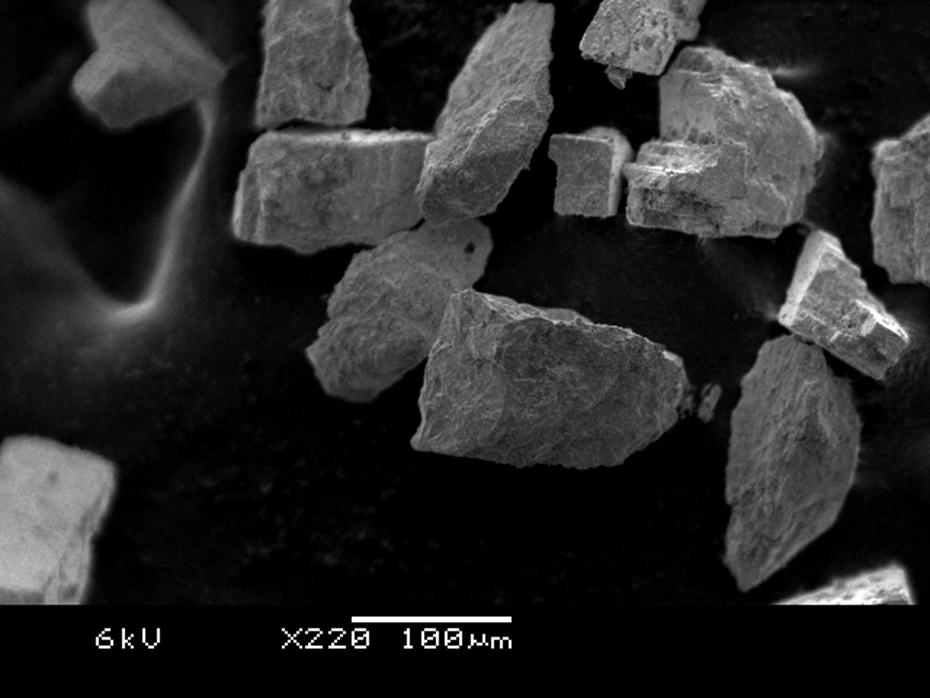


**Figure S37** SEM/EDS image of a microsample taken from the content of the object UC7321. Elemental Composition: Major: C (43.08) O (24.68) Pb (15.71) S (13.15); Minor: Cl (1.35) Zn (1.04); Trace: Fe (0.56) Ca (0.42).


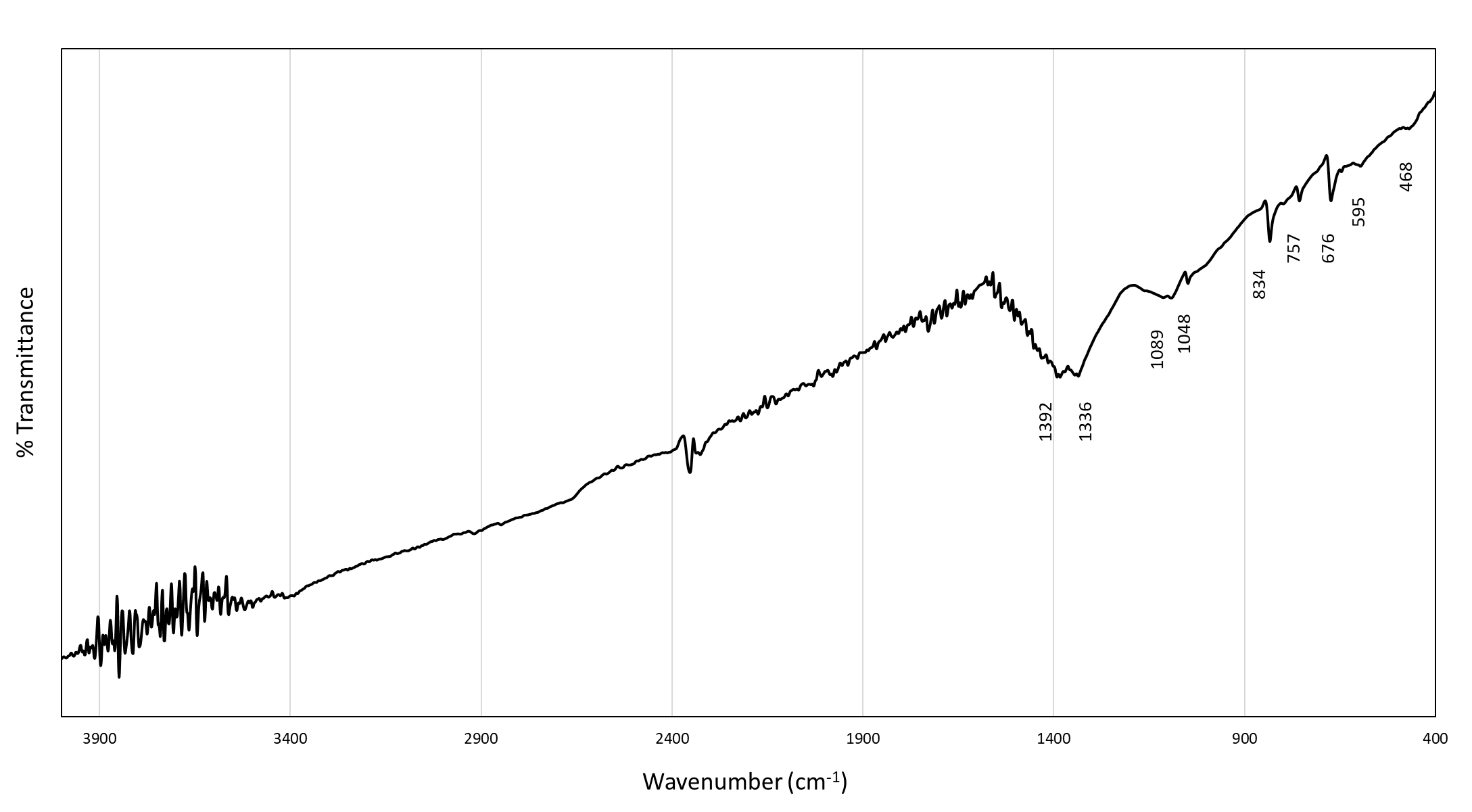


**Figure S38** FTIR spectrum of the sample from UC7321 displayed in percentage transmittance. The notated left drift of IR spectrum baseline likely due to the presence of heavy metals. A broad, strong peak at 1384 cm^-1^ and two weak, sharp peaks sharp peaks at 834 cm^-1^ and 674 cm^-1^ are attributed to cerussite (PbCO_3_) and are consistent with known references[^20^](https://paperpile.com/c/29uT4b/CiyO) (IRUG IMP00237, IMP00268).

**
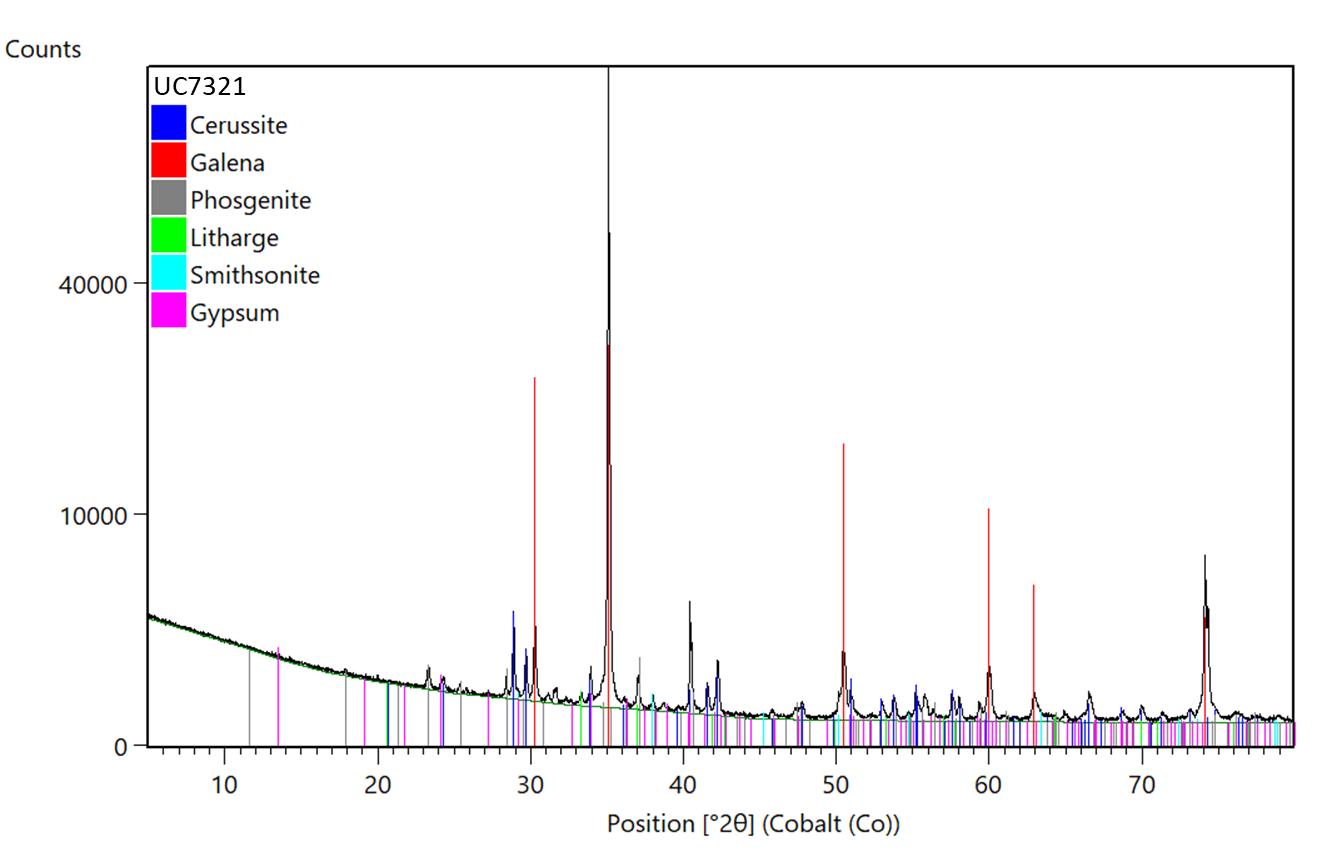
**

**Figure S39** X-ray diffractogram of the sample from UC7321. Cerussite, Galena, Phosgenite, Litharge, Smithsonite and Gypsum were identified.


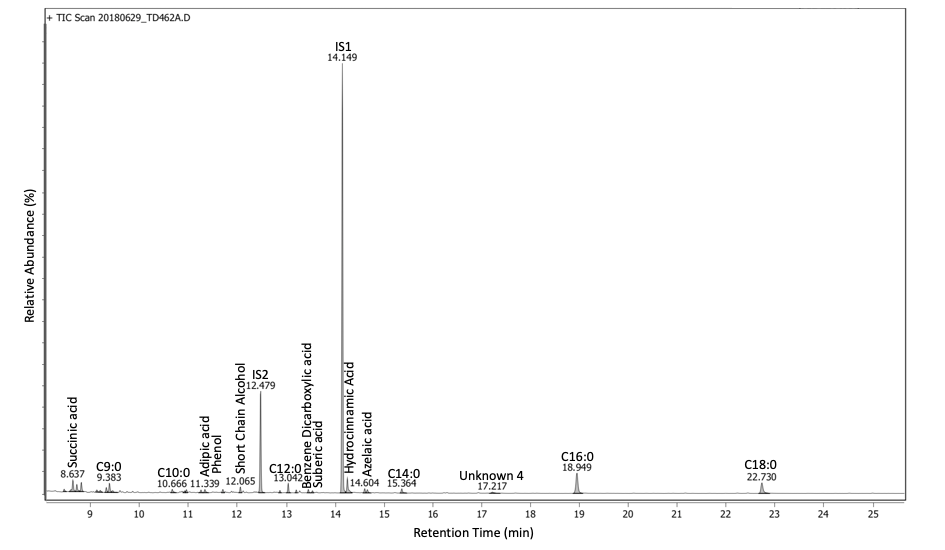

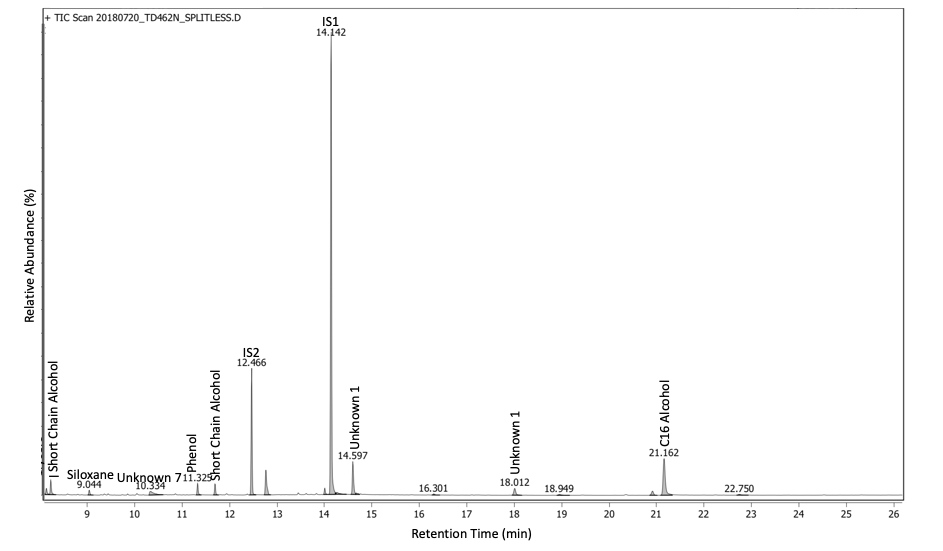


**Figure S40** GC/MS chromatograms obtained on the organic extract of the sample (UC7321). Top and bottom chromatograms correspond to the acidic and neutral fractions, respectively. Peak identifications are reported in Table S9.

**Table S9** Identification of the compounds present in the organic fraction of the sample (UC7321). TMS indicates a trimethylsilyl ester.

| **Time (min)** | **Compound** | **Peak area** |
| --- | --- | --- |
| *Acidic fraction* | | |
| 8.7194 | butanedioic acid, TMS | 509877 |
| 9.3874 | nonanoic acid, TMS | 724791 |
| 11.2591 | hexanedioic acid, TMS | 134846 |
| 12.4776 | hexadecane (IS2) | 8816058 |
| 12.7532 | 4-benzoic acid, TMS | 32408 |
| 13.0424 | dodecanoic acid, TMS | 677288 |
| 13.5394 | octanedioic acid, TMS | 115092 |
| 14.1503 | tridecanoic acid, TMS (IS1) | 37872806 |
| 14.2814 | vanillic acid, TMS | 124132 |
| 14.6602 | nonanedioic acid, TMS | 218341 |
| 15.3648 | tetradecanoic acid, TMS | 401114 |
| 18.9472 | hexadecanoic acid, TMS | 3130298 |
| 22.7292 | octadecanoic acid, TMS | 1733310 |
| *Neutral Fraction* | | |
| 8.1399 | glycerol, TMS | 263357 |
| 12.4663 | IS2 | 5654412 |
| 14.1411 | IS1 | 22215855 |
| 21.1619 | C27 alcohol | 21.162 |

**UC6742a**


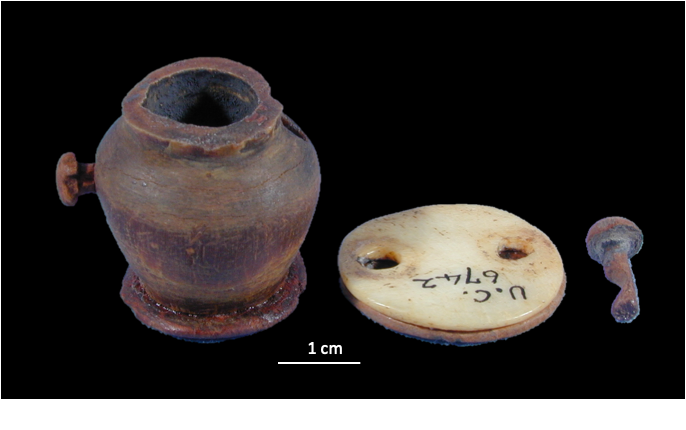


**Figure S41** Picture of a kohl pot (UC6742a) from Lahun, New Kingdom (early Dynasty XVIII), kept at the Petrie Museum. Object description: Horn kohl pot with wooden base, remainder of kohl (galena eye paint) inside; found inside casket UC6741. Sample description: Residue observed, sample taken from inside the vessel body. Image credit: Courtesy of the Petrie Museum of Egyptian Archaeology, UCL.


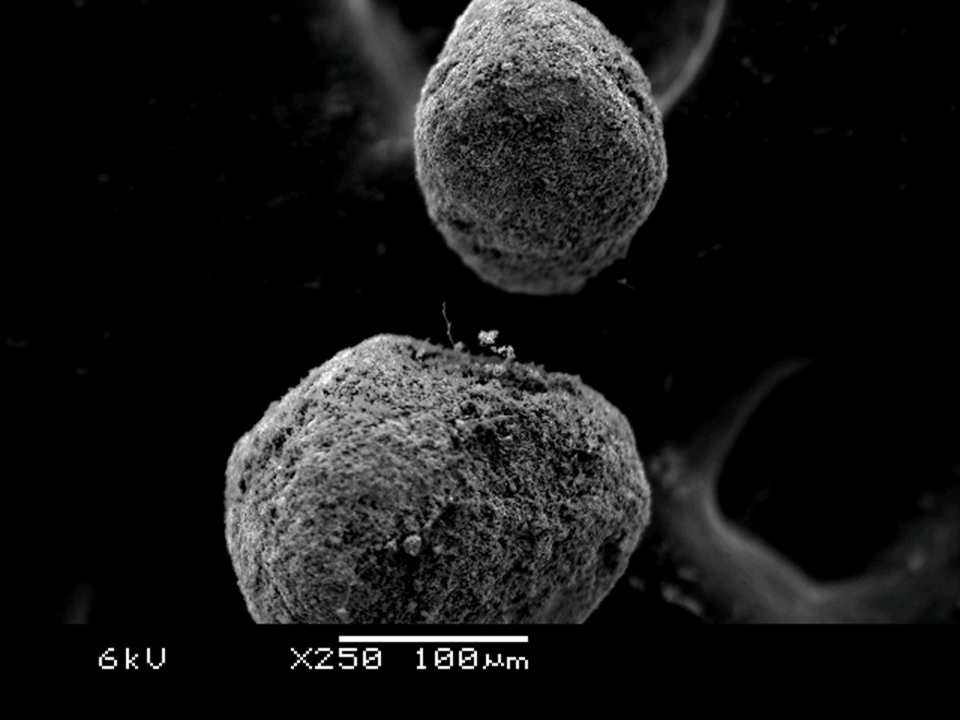


**Figure S42** SEM/EDS image of a microsample taken from the content of the object UC6742a. Images were acquired between 3-5 kV, with a spot intensity of 53, in secondary electron detector mode (SED). Elemental Composition: Major: O (65.87) C (26.59); Minor: Zn (2.98) S (2.59); Trace: Pb (0.96) Cl (0.27) Ca (0.25) Si (0.23).


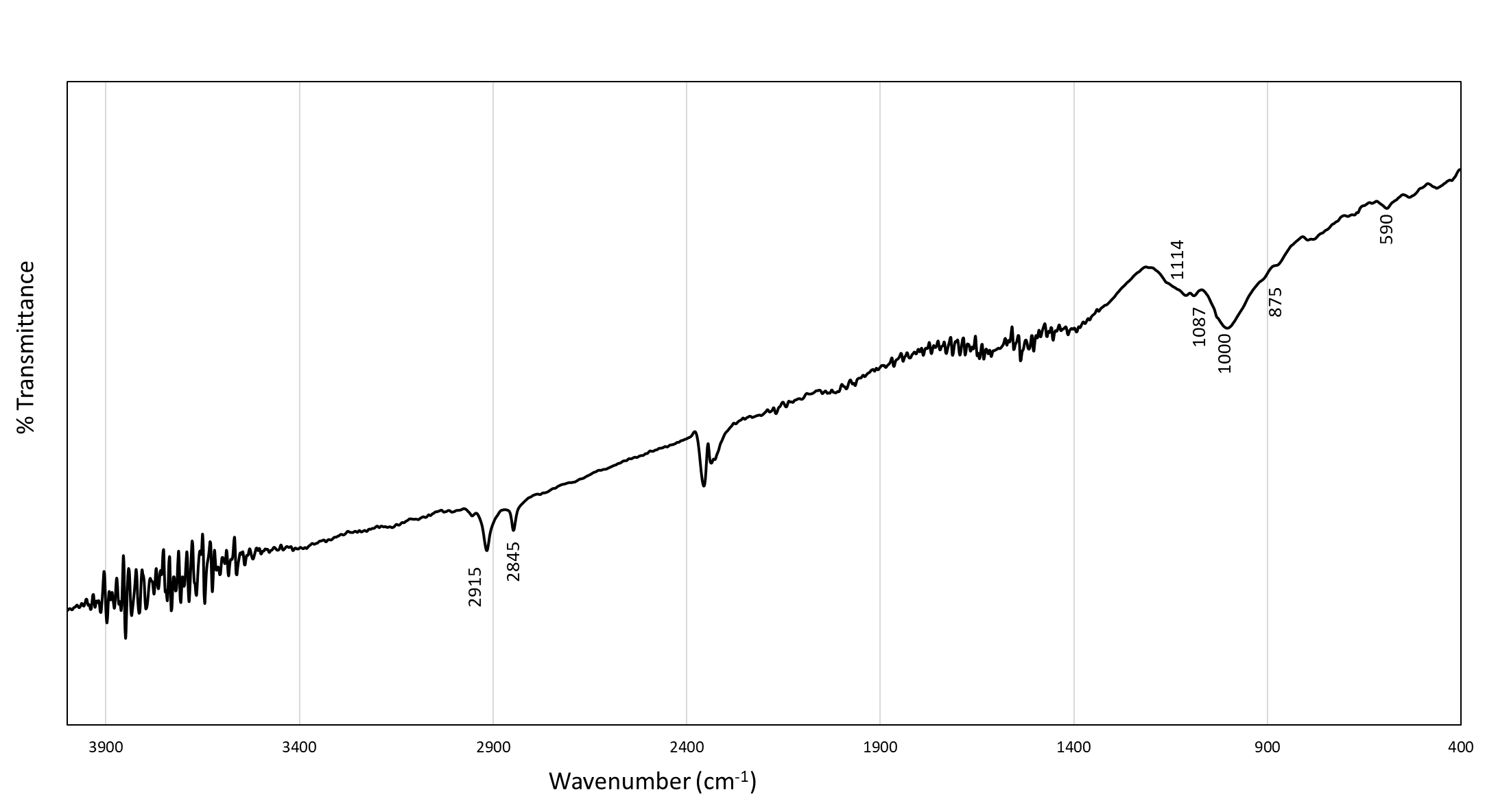


**Figure S43** FTIR spectrum of the sample a from UC6742 displayed in percentage transmittance. Left drift of baseline of IR spectrum consistent with heavy metals identified by SEM/EDS analysis. Weak signals associated with C-H stretches of aliphatics were identified at 2915 and 2845 cm^-1^ ^[^[^21^](https://paperpile.com/c/29uT4b/pfTm)^]^. This supports the presence of elemental carbon as a major component identified in the EDS analysis. Peaks in fingerprint between 1450 - 500 cm^-1^ region were too weak to confirm the presence of additional mineral constituents.


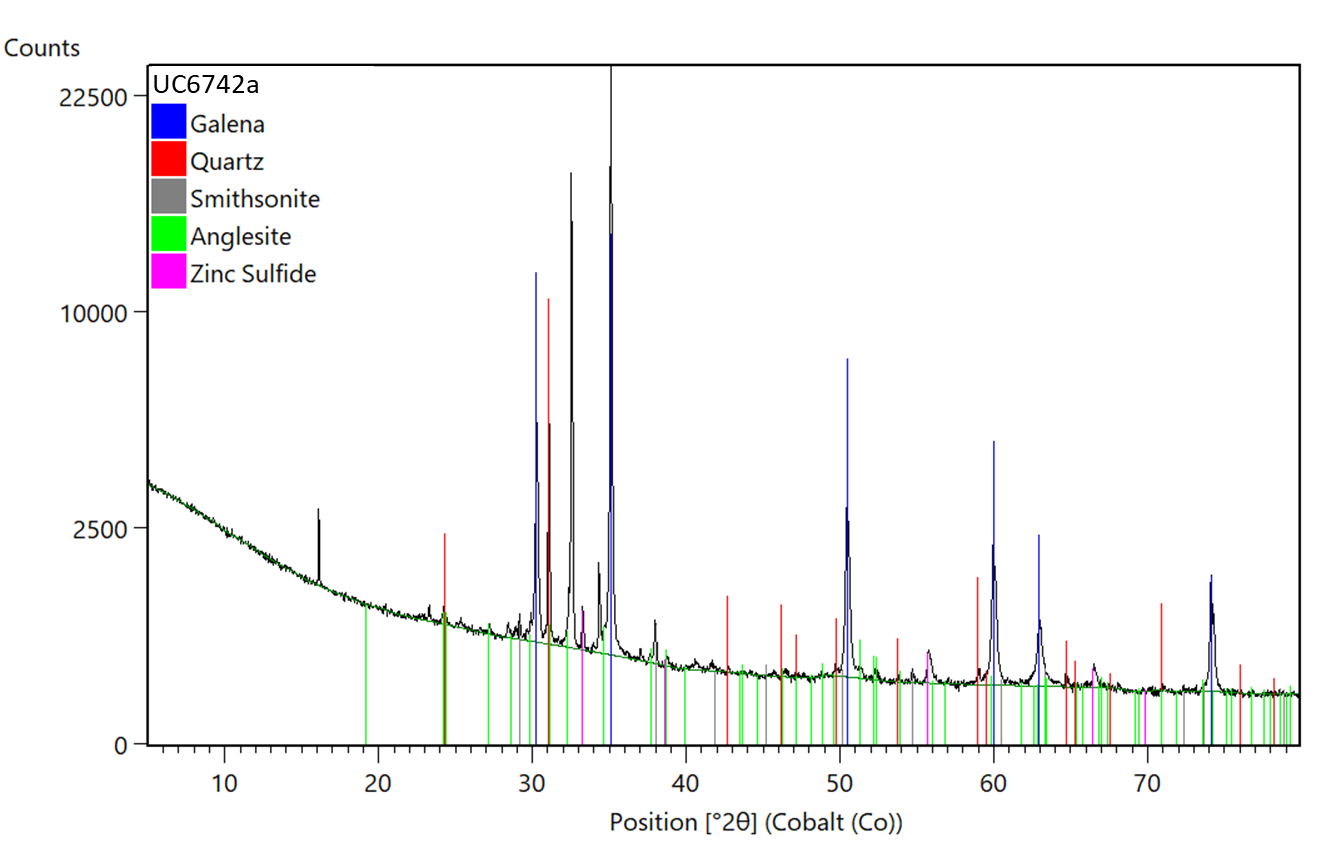


**Figure S44** X-ray diffractogram of the sample from UC6742a. Galena, Quartz, Smithsonite, Anglesite and Zinc Sulfide were identified.


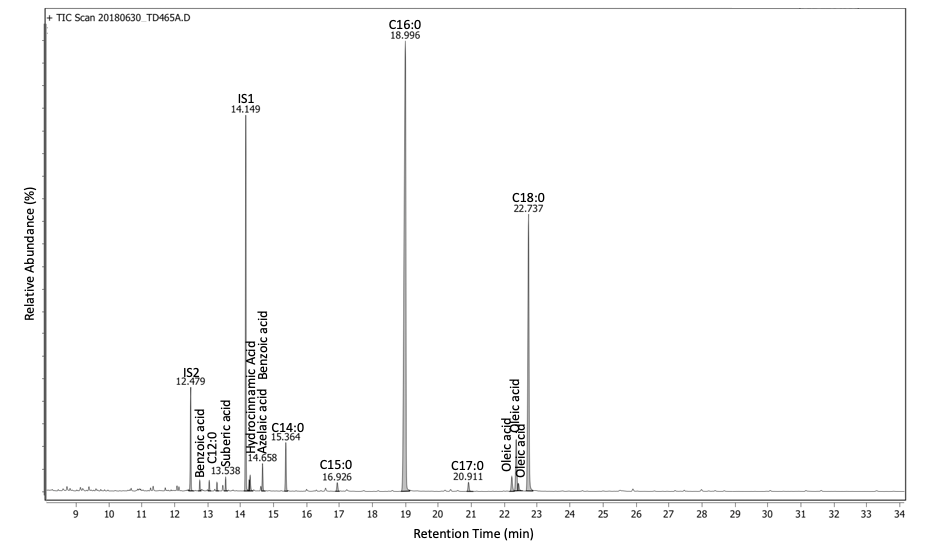

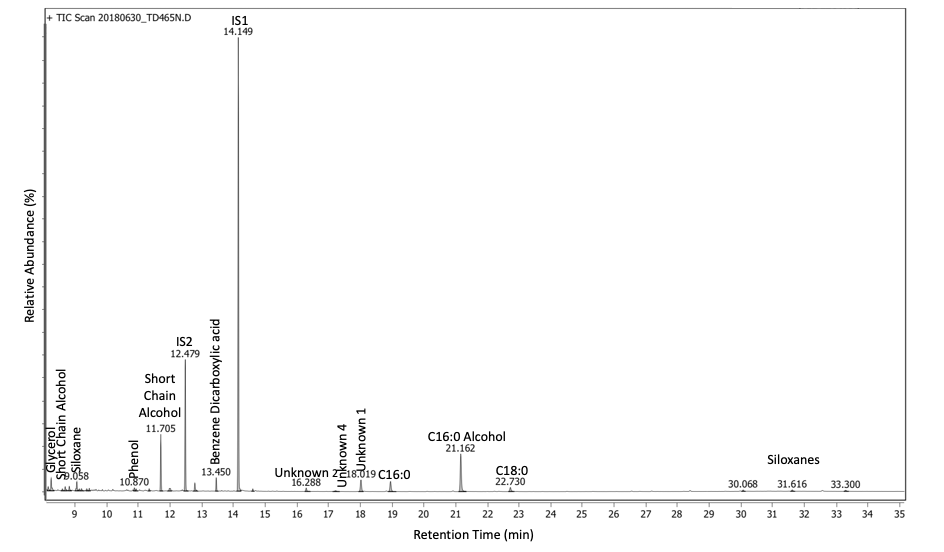


**Figure S45** GC/MS chromatograms obtained on the organic extract of the sample (UC6742a). Top and bottom chromatograms correspond to the acidic and neutral fractions, respectively. Peak identifications are reported in Table S10.

**Table S10** Identification of the compounds present in the organic fraction of the sample (UC6742a). TMS indicates a trimethylsilyl ester.

| **Time (min)** | **Compound** | **Peak area** |
| --- | --- | --- |
| *Acidic fraction* | | |
| 8.7224 | butanedioic acid, TMS | 414325 |
| 9.389 | nonanoic acid, TMS | 381866 |
| 11.262 | hexanedioic acid, TMS | 258661 |
| 11.8866 | undecanoic acid, TMS | 65208 |
| 12.4534 | heptanedioic acid, TMS | 399262 |
| 12.4785 | hexadecane (IS2) | 10963016 |
| 12.7542 | 3-benzoic acid, TMS | 1030574 |
| 13.0427 | dodecanoic acid, TMS | 991481 |
| 13.5403 | octanedioic acid, TMS | 1270306 |
| 14.1519 | tridecanoic acid, TMS (IS1) | 42072679 |
| 14.2836 | vanillic acid, TMS | 1795102 |
| 14.6611 | nonanedioic acid, TMS | 3020386 |
| 15.3664 | tetradecanoic acid, TMS | 5981668 |
| 15.9971 | decanedioic acid, TMS | 213890 |
| 16.0419 | syringic acid, TMS | 37960 |
| 17.7438 | undecanedioic acid, TMS | 92690 |
| 18.9922 | hexadecanoic acid, TMS | 122469504 |
| 19.8381 | ferulic acid, TMS | 1146 |
| 20.9134 | heptadecanoic acid, TMS | 1587203 |
| 21.169 | C18 alcohol, TMS | 40824 |
| 22.221 | octadecenoic acid, TMS (cis-) | 2617563 |
| 22.3594 | octadecenoic acid, TMS (trans-) | 9964648 |

**Table S10** Continued.

| **Time (min)** | **Compound** | **Peak area** |
| --- | --- | --- |
| 22.7355 | octadecanoic acid, TMS | 53681033 |
| 23.741 | possible, 14OH C16 dicarboxylic acid | 12454 |
| 23.97 | 15OH C16 dicarboxy | 5057 |
| 24.375 | nonadecanoic acid, TMS | 67940 |
| 25.0008 | DHA, TMS | 31668 |
| 25.904 | eicosanoic acid, TMS | 371407 |
| 27.1834 | diisooctyl phthalate | 145821 |
| 27.99 | monopalmatin, TMS | 247207 |
| 28.6817 | docosanoic acid, TMS | 79468 |
| 31.162 | C24:0 FA | 129340 |
| *Neutral Fraction* | | |
| 8.1554 | glycerol, TMS | 253836 |
| 12.4784 | hexadecane (IS2) | 12684523 |
| 14.1511 | tridecanoic acid, TMS (IS1) | 46899583 |
| 15.3629 | tetradecanoic acid, TMS | 34085 |
| 16.301 | branched pentadecanoic acid, TMS | 97289 |
| 16.437 | branched pentadecanoic acid, TMS | 80330 |
| 16.926 | pentadecanoic acid, TMS | 1316325 |
| 18.9487 | hexadecanoic acid, TMS | 1773472 |
| 20.205 | branched heptadecanoic acid, TMS | 164968 |
| 20.374 | branched heptadecanoic acid, TMS | 281075 |
| 22.7286 | octadecanoic acid, TMS | 735678 |
| 29.496 | HC27 | 6972 |

**UC7890**


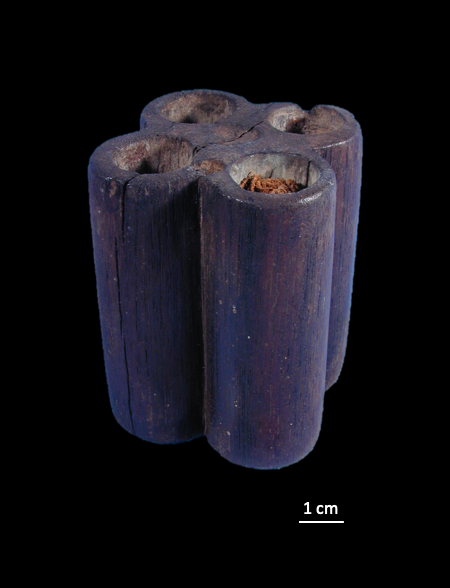


**Figure S46** Picture of a kohl pot (UC7890) from Gurob, New Kingdom (Dynasty XIX), kept at the Petrie Museum. Object description: Wooden kohl-pot; quadruple tubes, one with green powder (UC7890b), plug of linen probably belongs, one with black kohl (UC7890a), one empty, one cut through the bottom; bronze wire loop for holding stick, holes for another loop above. Sample description: Black sample (UC7890a) from inside the tube next to empty cylinder, green sample (UC7890b) taken from inside quad tube, specimen had a linen textile plug. Image credit: Courtesy of the Petrie Museum of Egyptian Archaeology, UCL.

**UC7890a**

**
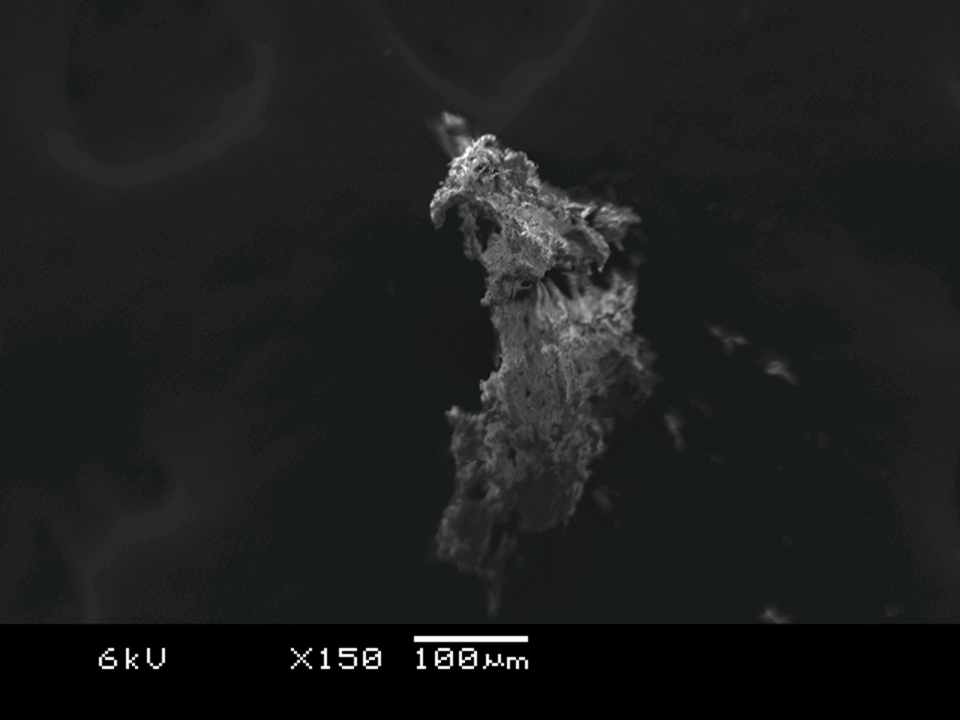
**

**Figure S47** SEM/EDS image of a microsample taken from the content of the object (UC7890a). Images were acquired between 3-5 kV, with a spot intensity of 53, in secondary electron detector mode (SED). Elemental Composition: Major: O (66.46) C (33.09); Trace: Na (0.34) Cl (0.03) Mg (0.02) Al (0.02) S (0.01) K (0.01) Ca (0.01).


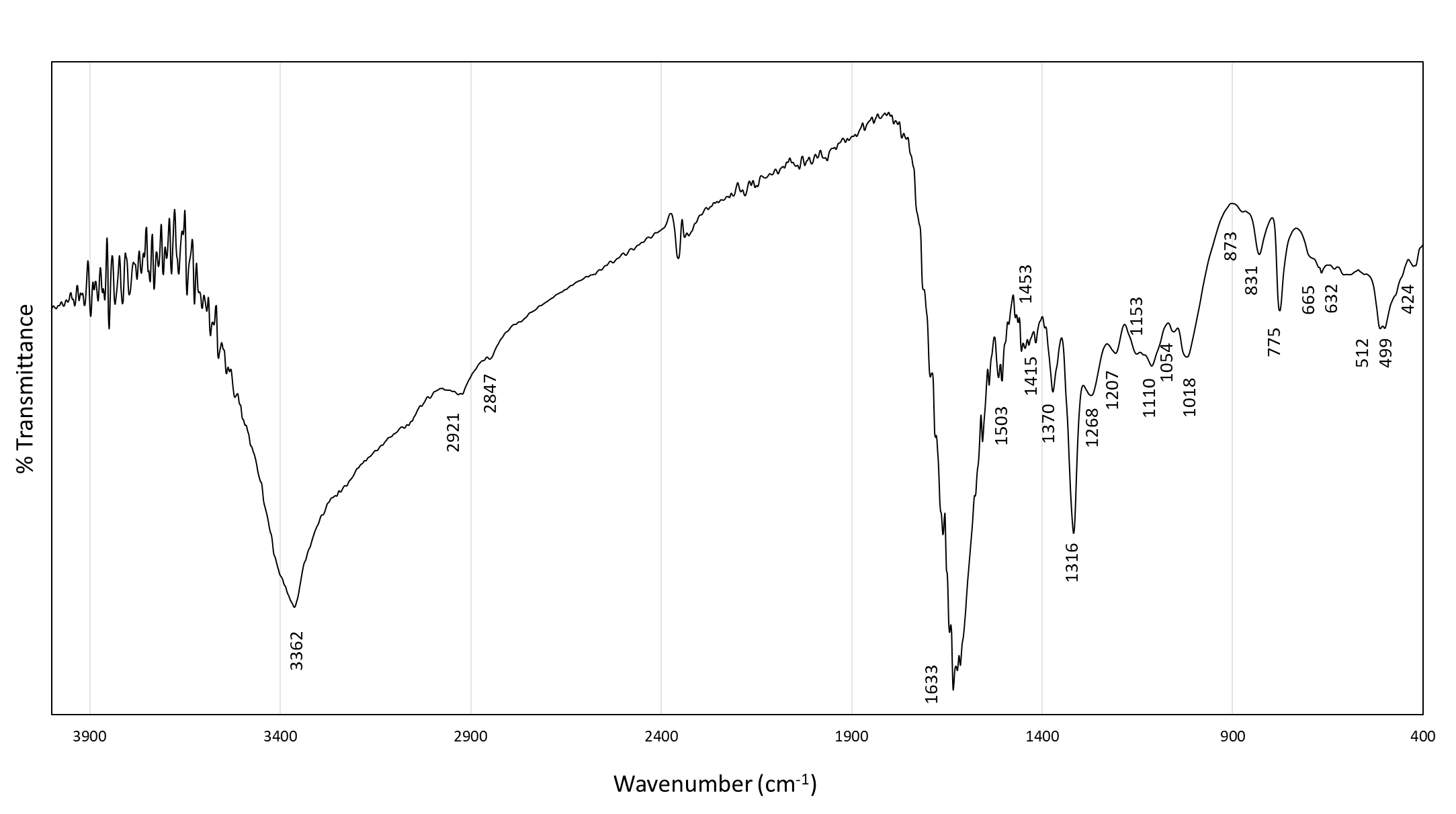


**Figure S48** FTIR spectrum of the sample a from UC7890 displayed in percentage transmittance. The obtained IR spectrum yielded a mixture of organic and inorganic constituents. It included: a strong, sharp peak attributed to an -OH stretch at 3360 cm^-1^, and weak C-H aliphatic stretch peaks at 2919 cm^-1^ and 2847 cm^-1^. Weak, sharp peaks 1503 cm^-1^ may be attributed to C=C-C of aromatic rings, while 1453 cm^-1^ may be associated with C-H deformation of CH_2_ and CH_3_; 1415 cm^-1^ may correspond to in-plane bending of vinyl (C-H) of olefinic groups, or a carbonate anion. A peak at 1370 cm^-1^ can be interpreted as C-H deformation of saturated aliphatic groups or -CH_3_ bending. Other notable peaks included: 1268 cm^-1^ (C-O stretch), 1110 cm^-1^ (C-O-C of esters and alcohols), and 1019 cm^-1^ (C-O bending or in plane C-H bending of aromatic groups)[^21^](https://paperpile.com/c/29uT4b/pfTm). Peaks at 1633 cm^-1^ (broad, asymmetric C-O vibration) and 1316cm^-1^ (symmetric C-O vibration), alongside peaks at 873, 775, 665, and 512 cm^-1^ may be attributed to calcium oxalate monohydrate (mainly whewellite)^71^, and is consistent with the XRD results identifying whelwellite in this sample. The remaining peaks could not be attributed to a particular source.


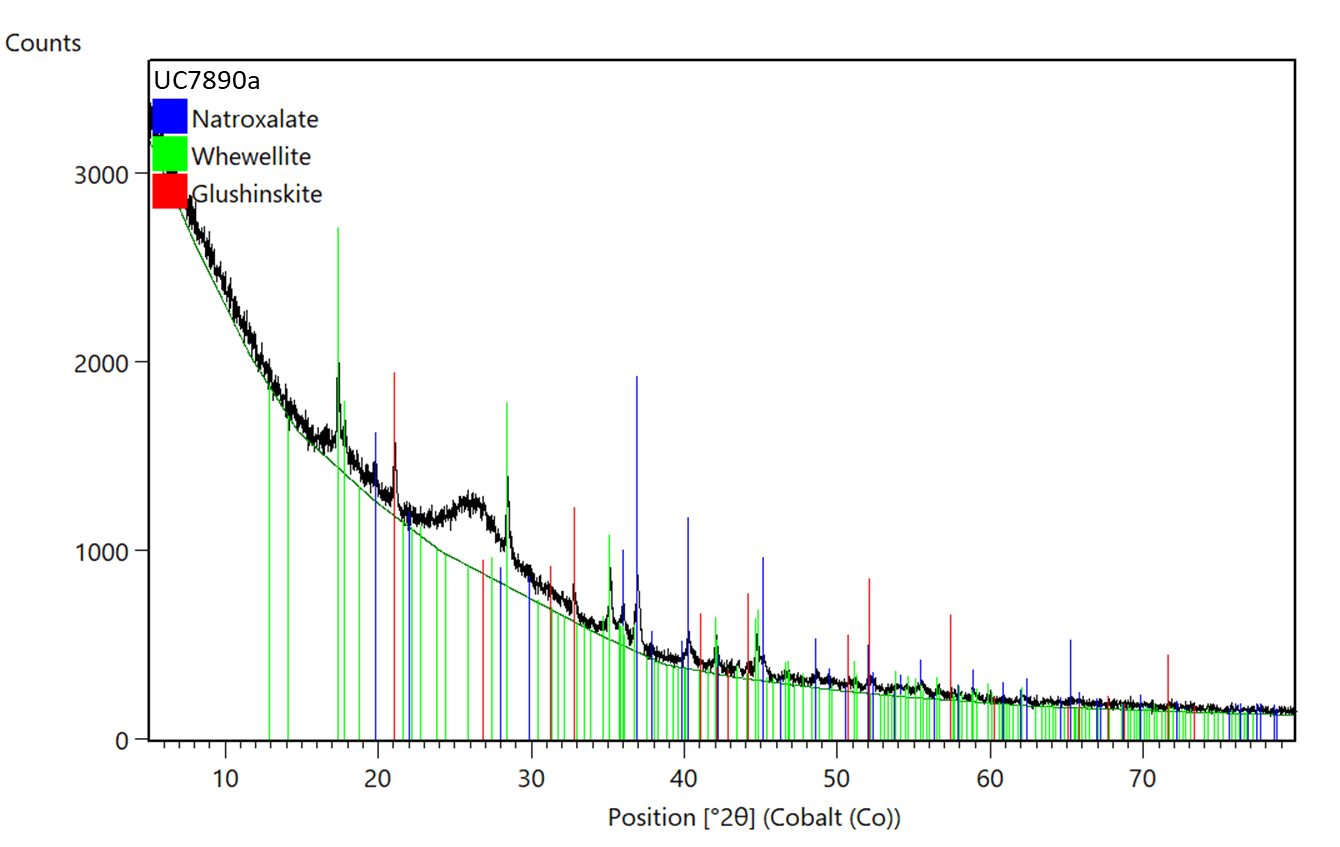


**Figure S49** X-ray diffractogram of the sample from UC7890a. Natroxalate, Whewellite and Glushinskite were identified.


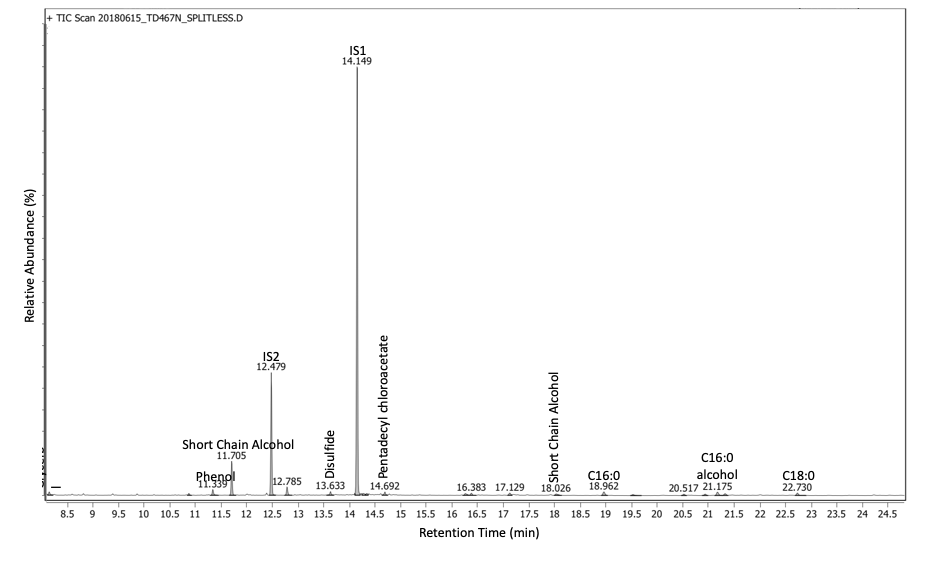

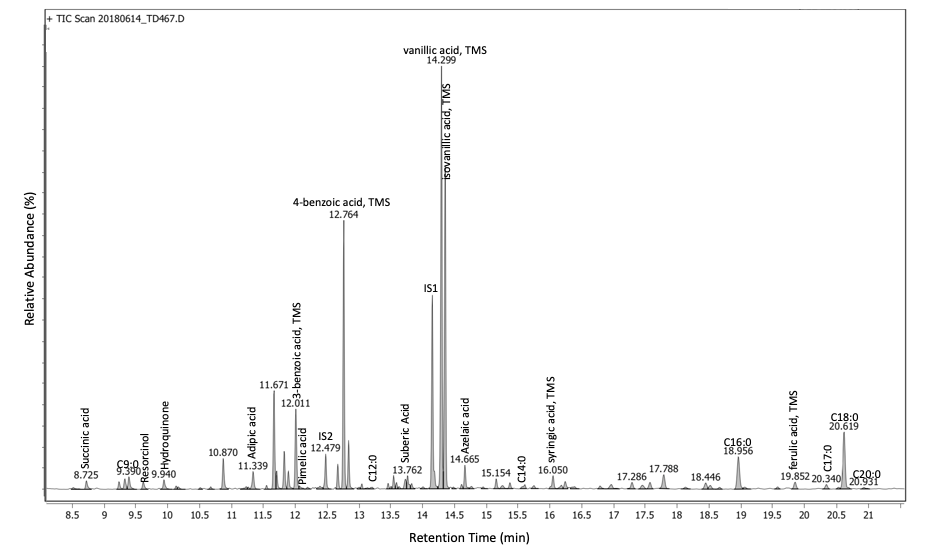


**Figure S50** GC/MS chromatograms obtained on the organic extract of the sample (UC7890a). Top and bottom chromatograms correspond to the acidic and neutral fractions, respectively. Peak identifications are reported in Table S11.

**Table S11** Identification of the compounds present in the organic fraction of the sample (UC7890a). TMS indicates a trimethylsilyl ester.

| **Time (min)** | **Compound** | **Peak area** |
| --- | --- | --- |
| *Acidic fraction* | | |
| 8.725 | butanedioic acid, TMS | 1338728 |
| 9.3924 | nonanoic acid, TMS | 1614468 |
| 9.6761 | resorcinol, TMS | 1231494 |
| 9.9423 | hydroquinone | 5106 |
| 11.2654 | hexanedioic acid, TMS | 121727 |
| 12.0107 | 3-benzoic acid, TMS | 7353217 |
| 12.4595 | heptanedioic acid, TMS | 389240 |
| 12.4819 | hexadecane (IS2) | 4034294 |
| 12.7636 | 4-benzoic acid, TMS | 30223480 |
| 13.048 | dodecanoic acid, TMS | 474758 |
| 13.5463 | octanedioic acid, TMS | 1223089 |
| 14.1546 | tridecanoic acid, TMS (IS1) | 23061116 |
| 14.2978 | vanillic acid, TMS | 50923664 |
| 14.346 | isovanillic acid, TMS | 50923664 |
| 14.6671 | nonanedioic acid, TMS | 2495428 |
| 15.3732 | tetradecanoic acid, TMS | 683758 |
| 16.0493 | syringic acid, TMS | 1746866 |
| 18.9589 | hexadecanoic acid, TMS | 6649194 |
| 19.8496 | ferulic acid, TMS | 1200275 |
| 20.931 | heptadecanoic acid | 220839 |
| 22.7341 | octadecanoic acid, TMS | 4906970 |
| 25.913 | eicosanoic acid, TMS | 119331 |

**Table S11** Continued.

| **Time (min)** | **Compound** | **Peak area** |
| --- | --- | --- |
| *Neutral Fraction* | | |
| 8.154 | glycerol, TMS | 209581 |
| 9.3896 | nonanoic acid, TMS | 54999 |
| 12.4811 | hexadecane (IS2) | 9031731 |
| 13.0446 | dodecanoic acid, TMS | 13382 |
| 14.1538 | tridecanoic acid, TMS (IS1) | 33499163 |
| 18.9643 | hexadecanoic acid, TMS | 187487 |
| 22.734 | octadecanoic acid, TMS | 128259 |
| 27.1921 | diisooctyl phthalate | 207043 |

**UC7890b**

**
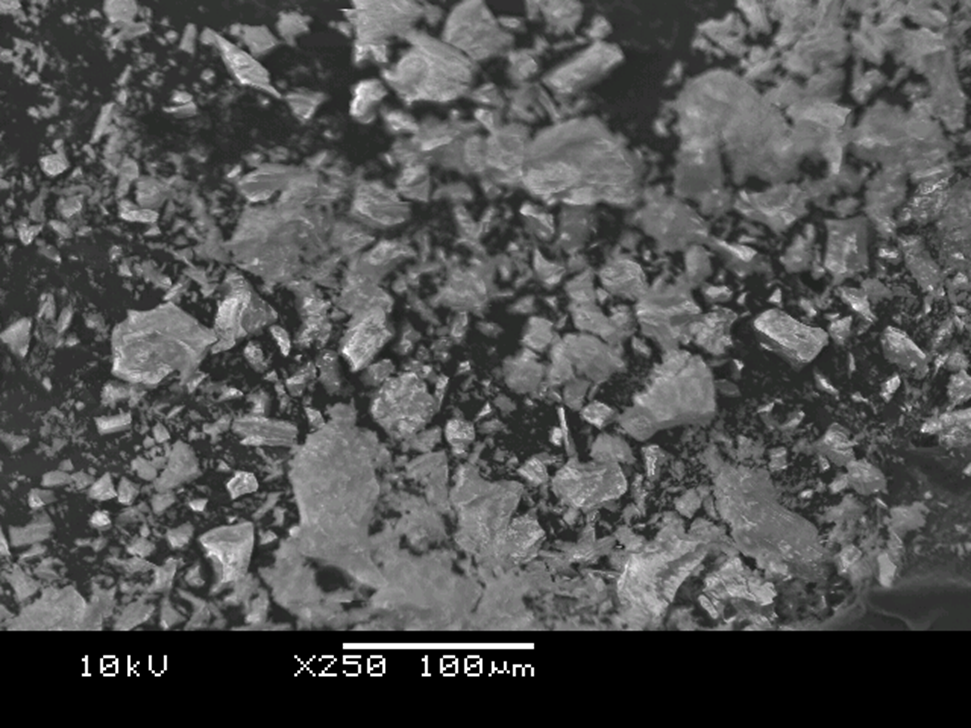
**

**Figure S51** SEM/EDS image of a microsample taken from the content of the object (UC7890b). Images were acquired between 3-5 kV, with a spot intensity of 53, in secondary electron detector mode (SED). Elemental Composition: Major: C (42.30) O (37.93); Minor: Si (8.36) Cu (3.98) Al (2.01) Cl (1.89) Ca (1.86); Trace: K (0.55) Fe (0.38) P (0.29) Mg (0.28) S (0.16).


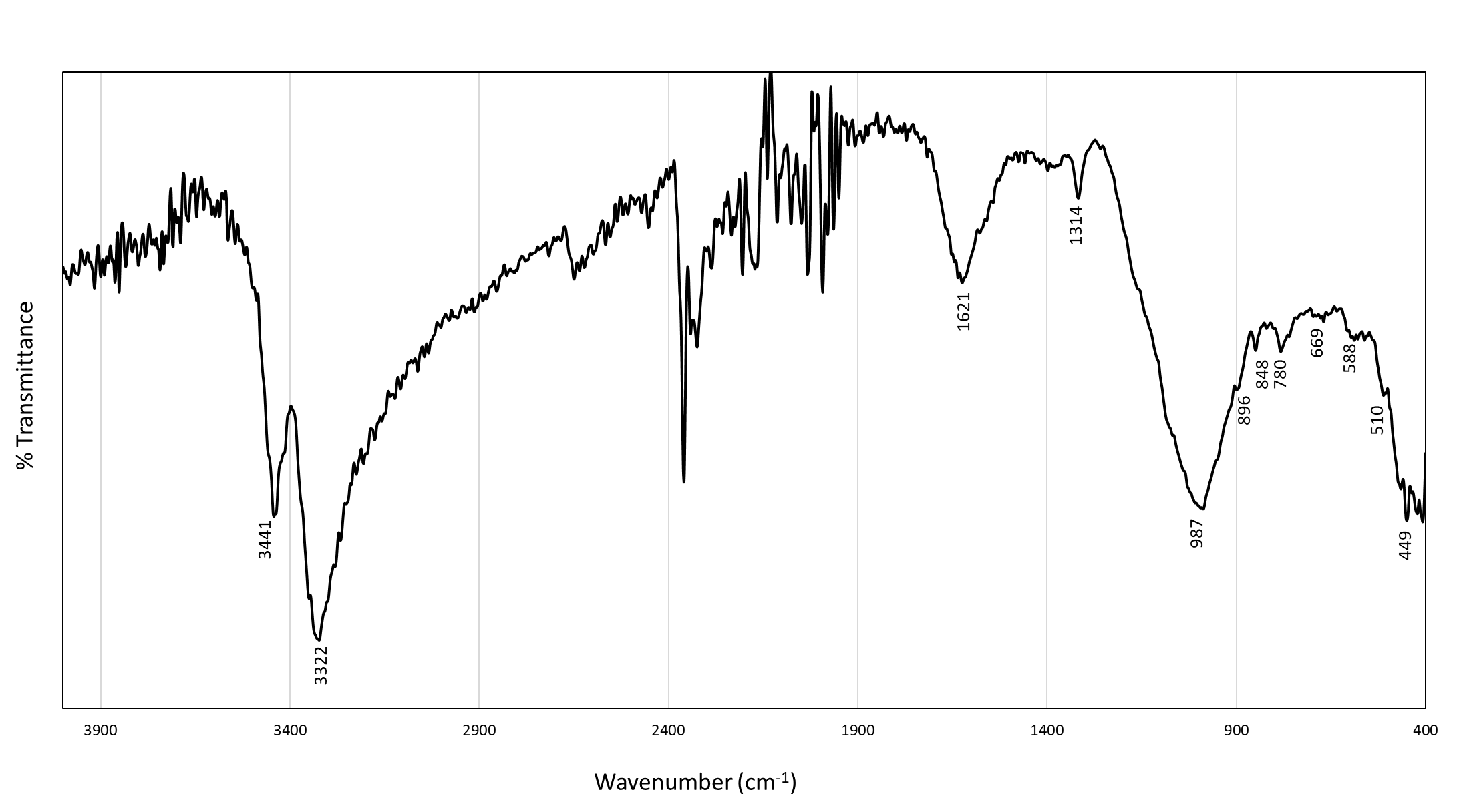


**Figure S52** FTIR spectrum of the sample b from UC7890 displayed in percentage transmittance. The IR spectrum indicates the presence of a mixture of inorganic and organic materials. Bands typical of calcium oxalate (CaC_2_O_4_) were identified at 1621 cm^−1^, 1314 cm^−1^ and 780 cm^−1^ and can be attributed to asymmetric C=O stretching, symmetric C=O stretching, and O–C=O stretching, respectively. The remaining identified peaks are consistent with atacamite (Cu_2_Cl(OH)_3_) and are consistent with references to material and archaeological specimens. Strong peaks at 3441 cm^-1^ and 3322 cm^-1^ may be attributed to -OH stretching vibrations, while the bands at bands at 987 cm^−1^, 896 cm^−1^, and 848 cm^−1^ may be due to -OH deformation. The CuCl and CuO vibrations may be interpreted based on the presence of peaks at 588 cm^−1^, 510 cm^−1^, and 449 cm^−1^ ^[^[^20,41^](https://paperpile.com/c/29uT4b/CiyO+bHgA)^]^ (IRUG IMP00198). These findings are consistent with the XRD results. Peaks at 780 and 449 cm^-1^ were identified in experimentally made Egyptian Blue pigments^72^, however, none of the remaining peaks match with IR spectra from known samples of archaeological Egyptian blue or green (particularly the notable triplet peaks between 1000-1160 cm), nor from the geological material from which they derived^73,74^. It is therefore unlikely to determine that Egyptian blue or green were the sources of this material. Peaks indicative of silicon-containing minerals, as per the elemental composition were not identifiable in the IR spectrum. XRD analysis confirmed the presence of quartz (SiO_2_) in this sample.


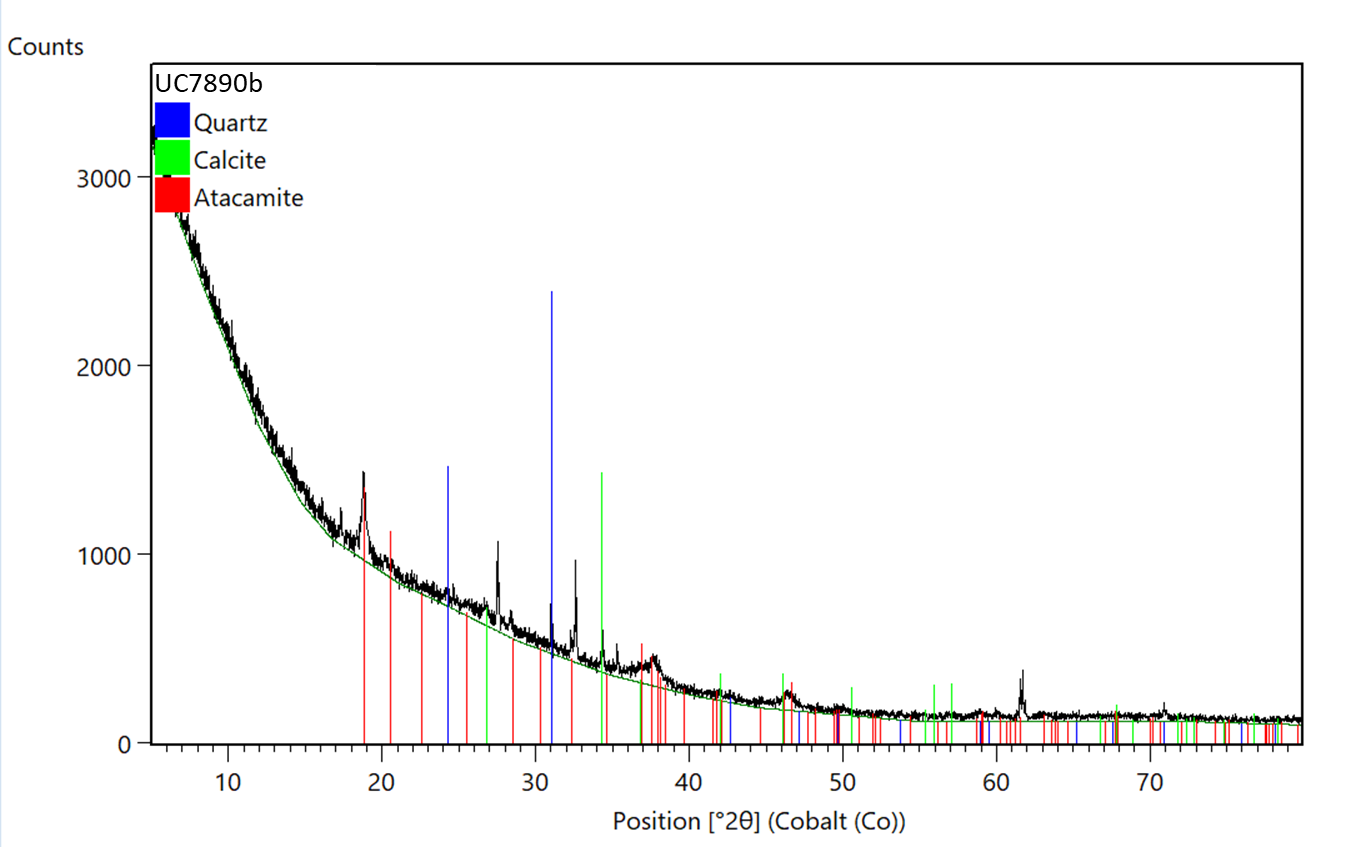


**Figure S53** X-ray diffractogram of the sample from UC7890b. Quartz, Calcite and Atacamite were identified.

GC/MS analysis was not conducted on the sample UC7890b as analyses with SEM/EDS and FTIR did not indicate any significant presence of organic materials.

**UC43107**


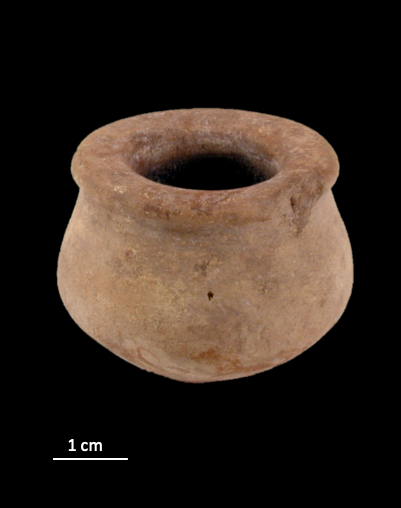


**Figure S54** Picture of a kohl pot (UC43107) from Abydos, Second Intermediate Period, kept at the Petrie Museum. Object description: Small marl ware pottery jar. Kohl on the interior. Sample description: Residue observed, sample taken from inside vessel. Image credit: Courtesy of the Petrie Museum of Egyptian Archaeology, UCL.

**
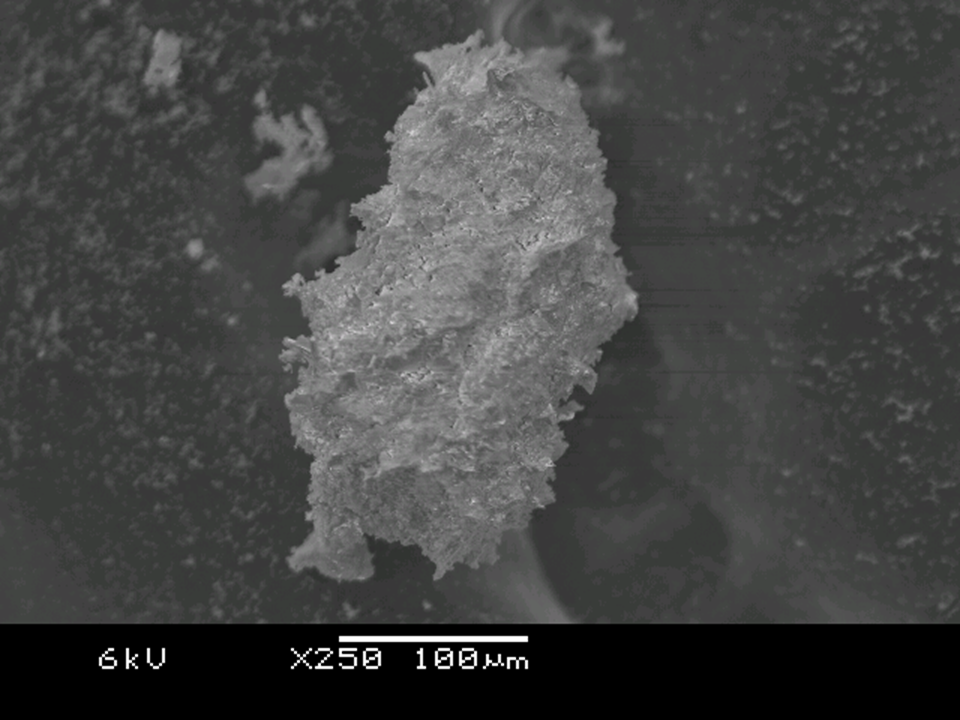
**

**Figure S55** SEM/EDS image of a microsample taken from the content of the object UC43107. Images were acquired between 3-5 kV, with a spot intensity of 53, in secondary electron detector mode (SED). Elemental Composition: Major: O (59.41) Si (17.94); Minor: Al (6.12) Cu (4.00) Ca (3.28) Na (2.18) Cl (1.75) K (1.69) Fe (1.65) Mg (1.04); Trace: P (0.49) S (0.30) Ti (0.16).


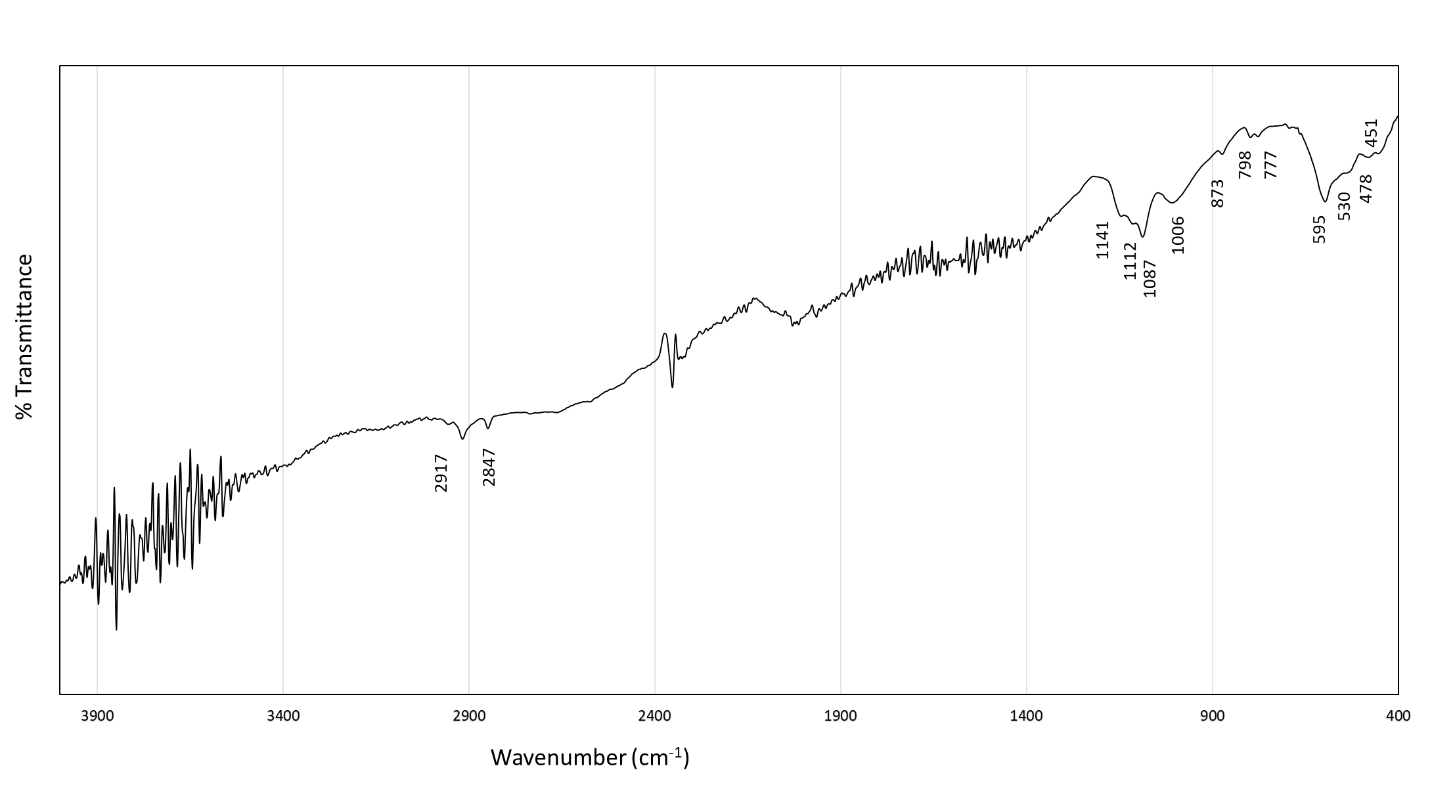


**Figure S56** FTIR spectrum of the sample from UC43107 displayed in percentage transmittance. Left baseline drift of IR spectrum consistent with heavy metal presence identified by SEM/EDS. Weak signals associated with C-H stretches of aliphatics were identified at 2917 and 2845 cm^-1^ [^21^](https://paperpile.com/c/29uT4b/pfTm) suggesting an additional organic component that was not found in the elemental compositional analysis above. This sample also yielded peaks consistent with the presence of silicates (especially peaks at 794 cm^-1^, 775 cm^-1^, and 692 cm^-1^), and possible aluminium silicon oxide minerals (1006 cm^-1^ and 530 cm^-1^)[^25^](https://paperpile.com/c/29uT4b/UjEu). A peak at 873 cm^-1^ may be tentatively attributed to the presence of calcium carbonate minerals[^18,25^](https://paperpile.com/c/29uT4b/1NCe+UjEu).


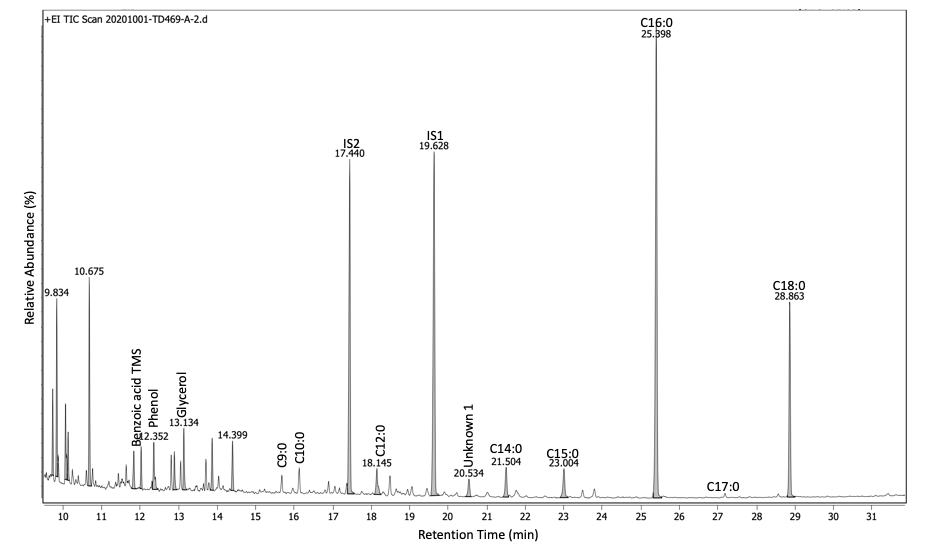

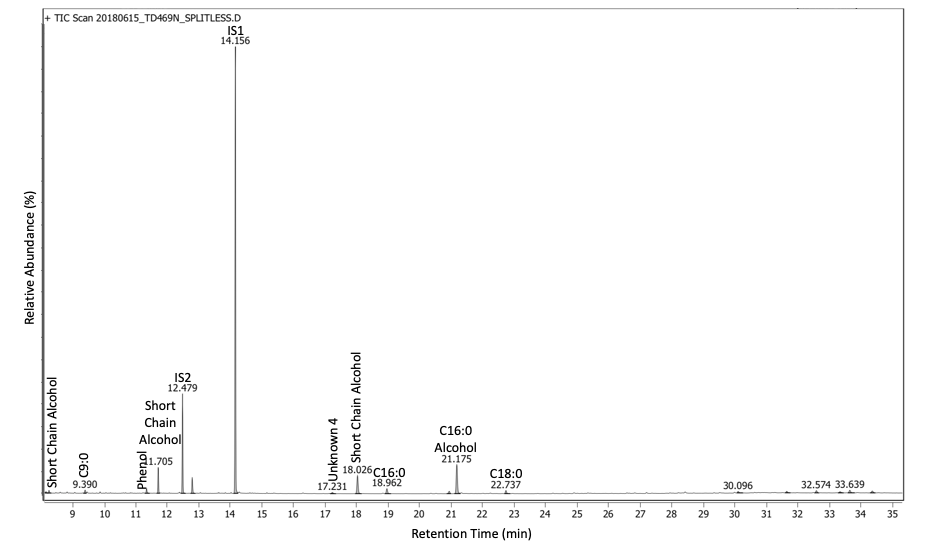
**Figure S57** GC/MS chromatograms obtained on the organic extract of the sample (UC43107). Top and bottom chromatograms correspond to the acidic and neutral fractions, respectively. Peak identifications are reported in Table S12.

**Table S12** Identification of the compounds present in the organic fraction of the sample (UC43107). TMS indicates a trimethylsilyl ester.

| **Time (min)** | **Compound** | **Peak area** |
| --- | --- | --- |
| *Acidic fraction* | | |
| 13.0534 | octanoic acid, TMS | 3908014 |
| 13.2828 | glycerol, TMS | 234705 |
| 14.3992 | nonanoic acid, TMS | 8109275 |
| 16.8949 | undecanoic acid, TMS | 2156549 |
| 17.4414 | hexadecane (IS2) | 2387483 |
| 18.1484 | dodecanoic acid, TMS | 4824878 |
| 19.6383 | tridecanoic acid (IS1) | 96279615 |
| 20.5394 | nonanedioic acid, TMs | 2422774 |
| 20.552 | terephthalic acid, TMS | 4127644 |
| 21.5199 | tetradecanoic acid, TMS | 7942208 |
| 23.4946 | pentadecanoic acid, TMS | 1858429 |
| 25.411 | hexadecanoic acid, TMS | 121470197 |
| 27.193 | heptadecanoic acid, TMS | 876823 |
| 28.574 | octadecenoic acid, TMS | 623391 |
| 28.8737 | octadecanoic acid, TMS | 42693762 |
| 31.4001 | DHA, TMS | 255114 |
| *Neutral Fraction* | | |
| 11.6006 | butyric acid, TMS | 316450 |
| 13.2803 | glycerol, TMS | 816919 |
| 14.3967 | nonanoic acid, TMS | 615071 |
| 17.4394 | hexadecane (IS2) | 66534883 |

**Table S12** Continued.

| **Time (min)** | **Compound** | **Peak area** |
| --- | --- | --- |
| 19.6323 | tridecanoic acid, TMS (IS1) | 76172669 |
| 25.3932 | hexadecanoic acid | 3148913 |
| 28.8618 | octadecanoic acid | 1137644 |

**Appendix 3**

***GC/MS data interpretation and unknown compounds***

**Table S13** List of materials and corresponding biomarkers used for GC/MS data interpretation.

| **Material** | **Compound markers** | **Source** |
| --- | --- | --- |
| Castor oil | ricinolaic acid | Colombini, Modugno and Ribechini (2005)^59^ |
| Bitumen | hopanes and steranes, complete range of homologous series of n-alkanes, pristane and phytane | Colombini [*et al.*](https://paperpile.com/c/29uT4b/dKxG+lmTq) (2005)^42^;  Clark, Ikram and Evershed (2016)^60^ |
| Frankincense/  Olibanum | α-boswellic acid, β-boswellic acid, 3-*O*-acetyl-α-boswellic acid, *O*-acetyl-β-boswellic acid | Evershed *et al.* (1997)^62^;  Culioli [*et al.*](https://paperpile.com/c/29uT4b/6Oj2+SjLr) (2003)^61^ |
| Beeswax | even number long-chain n-alcohols, odd-number long chain n-alkanes, 14-hydroxyhexadecanedioic acid and 15-hydoxyhexadecanedioic acid | [Ribechini *et al.*](https://paperpile.com/c/29uT4b/c2Ss+nfPT) (2008)^39^;  Rageot [*et al.* (2016)](https://paperpile.com/c/29uT4b/c2Ss+nfPT)^63^ |
| Myrrh | alpha- and beta- amyrin, euphanes, oleanes, and cycloartanes | Buckley and Evershed (2001)^51^;  Hamm, Bleton and Tchapla (2004)^64^ |
| Benzoin Resin | cinnamic acid, 4-hydroxybenzaldehyde, resorcinol, vanillin, 3- hydroxybenzoic acid, vanillic acid, cinnamyl alcohol | Mills and White (1987)^43^;  Modugno, Ribechini and Colombini (2006)[^45^](https://paperpile.com/c/29uT4b/evyD+hpEI) |
| Storax Resin | cinnamic acid,, cinnamyl alcohol, oleanonic acid, oleanolic acid | [Modugno, Ribechini and Colombini (2006)^45^](https://paperpile.com/c/29uT4b/evyD) |
| *Pineacae* Resin | abietane diterpenoids, notably dehydroabietic acid (DHA), 7-oxo-DHA, and other oxidised abietanes | [Colombini *et al.* (2005)^42^](https://paperpile.com/c/29uT4b/dKxG) |
| Sandarac (Cupressaceae) | pimaranes (esp. sandaracopimaric acid), sugiol, and communic acid | [Devièse *et al.*](https://paperpile.com/c/29uT4b/Rx5D+ZnyE) (2010)^65^;  [Devièse *et al.* (2017)^19^](https://paperpile.com/c/29uT4b/Rx5D+ZnyE) |

**Table S13** continued.

| Labdanum | laurifolic, cistenolic and labdanolic acids | Hamm, Bleton and Tchapla (2004)^64^;  Colombini and Modugno (2009)[^66^](https://paperpile.com/c/29uT4b/Tte9+Jljk) |
| --- | --- | --- |
| Mastic | moronic acid, masticadienonic acid, iso-masticadienonic acid, nor-olean-17-en-3one | Stern [*et al.* (2003)^67^](https://paperpile.com/c/29uT4b/vBhk+Uf39);  Regert and Rolando (2002)^68^ |

**Table S14** Unknown compounds identified by GC/MS in the samples included in this study.


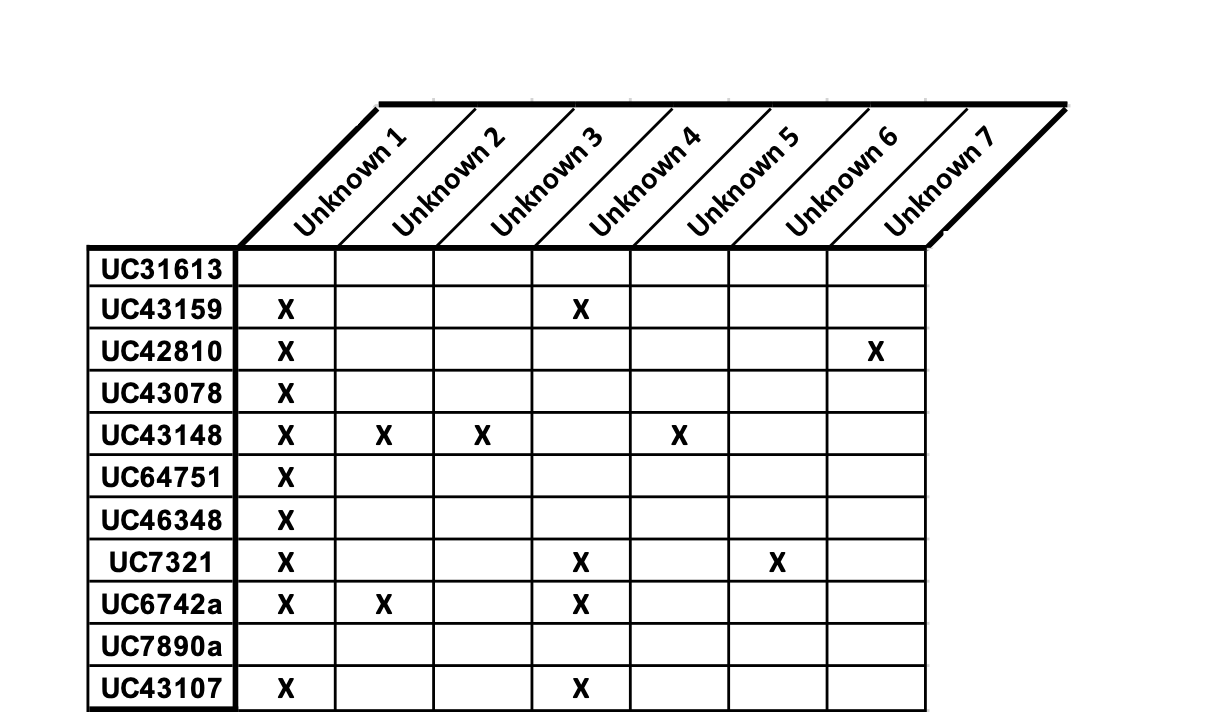


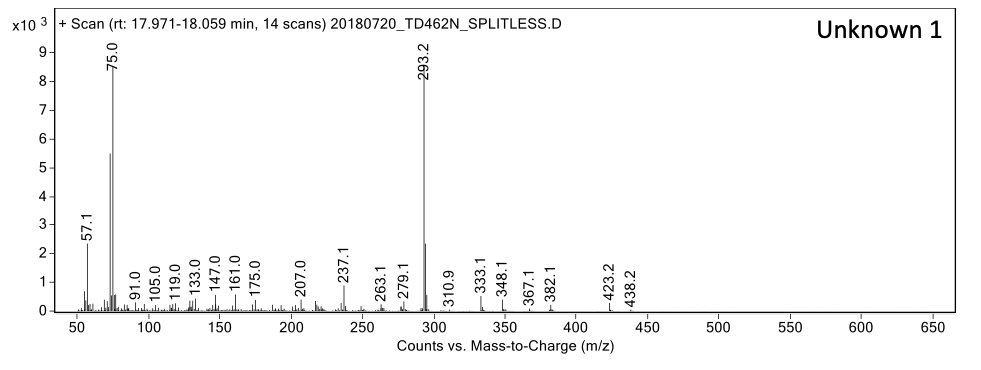
**Figure S58** Mass spectrum of unknown compound 1.


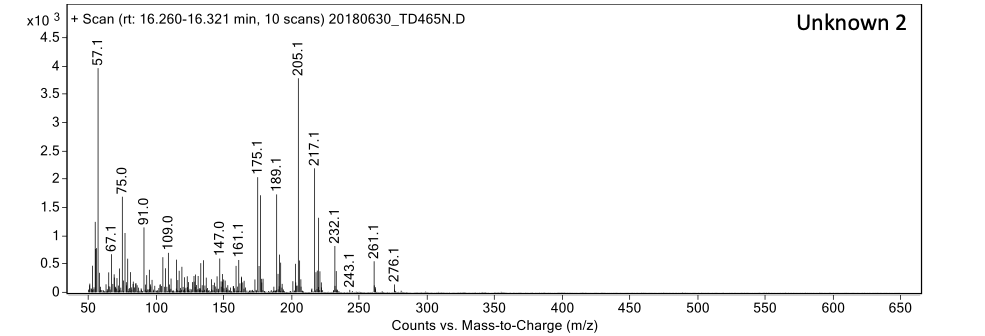
**Figure S59** Mass spectrum of unknown compound 2.


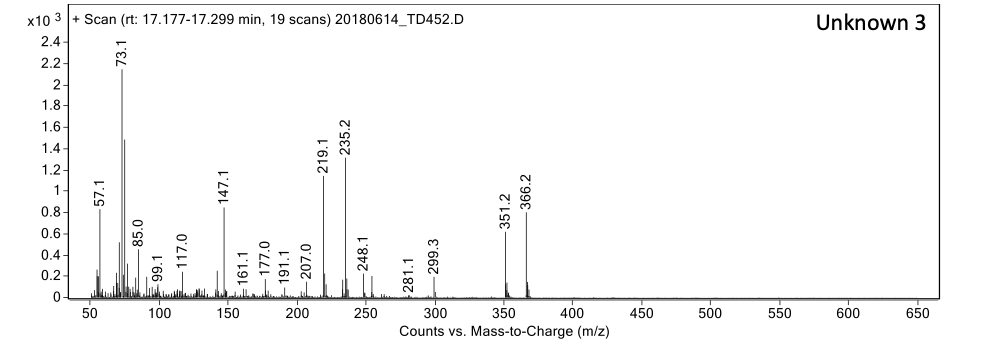
**Figure S60** Mass spectrum of unknown compound 3.


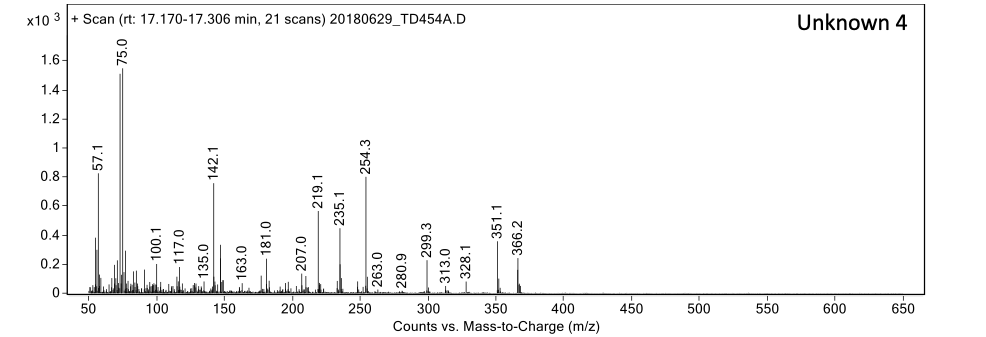
**Figure S61** Mass spectrum of unknown compound 4.


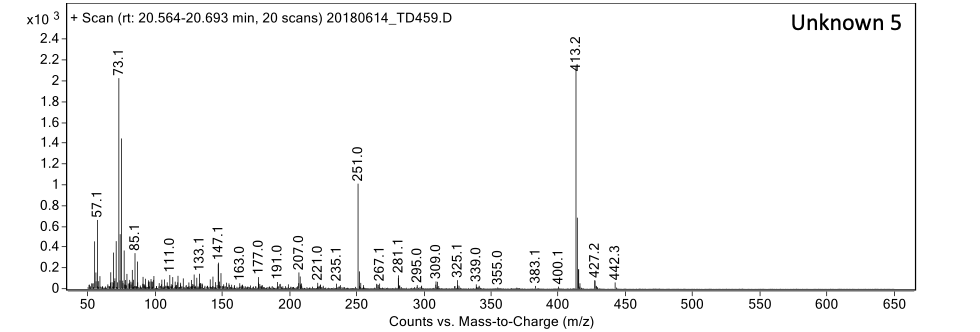
**Figure S62** Mass spectrum of unknown compound 5.


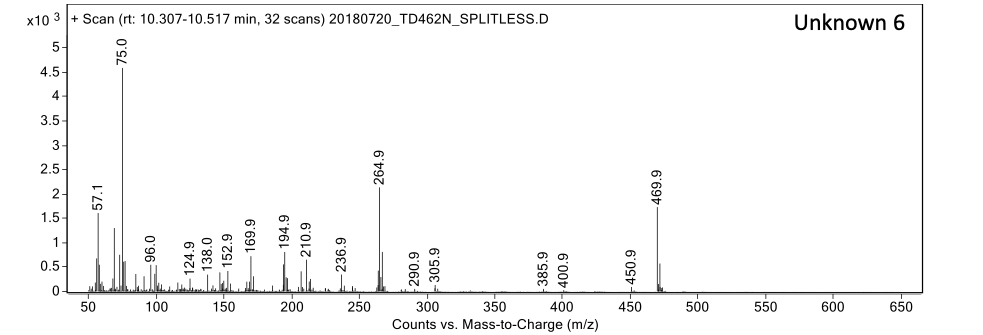
**Figure S63** Mass spectrum of unknown compound 6.


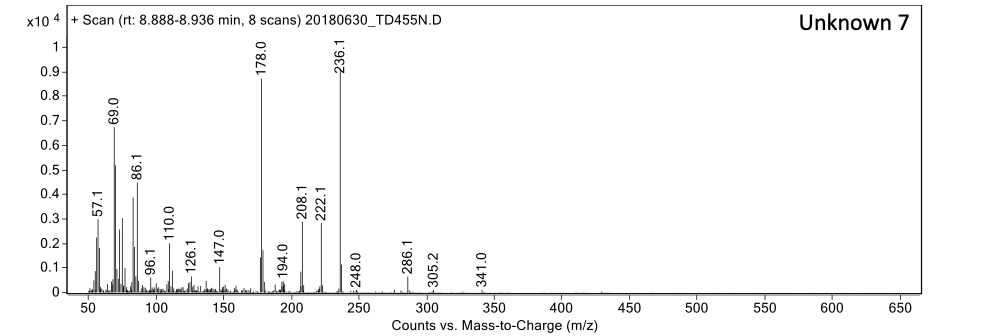
**Figure S64** Mass spectrum of unknown compound 7.

**Appendix 4**

**Patterns, time periods and locations**


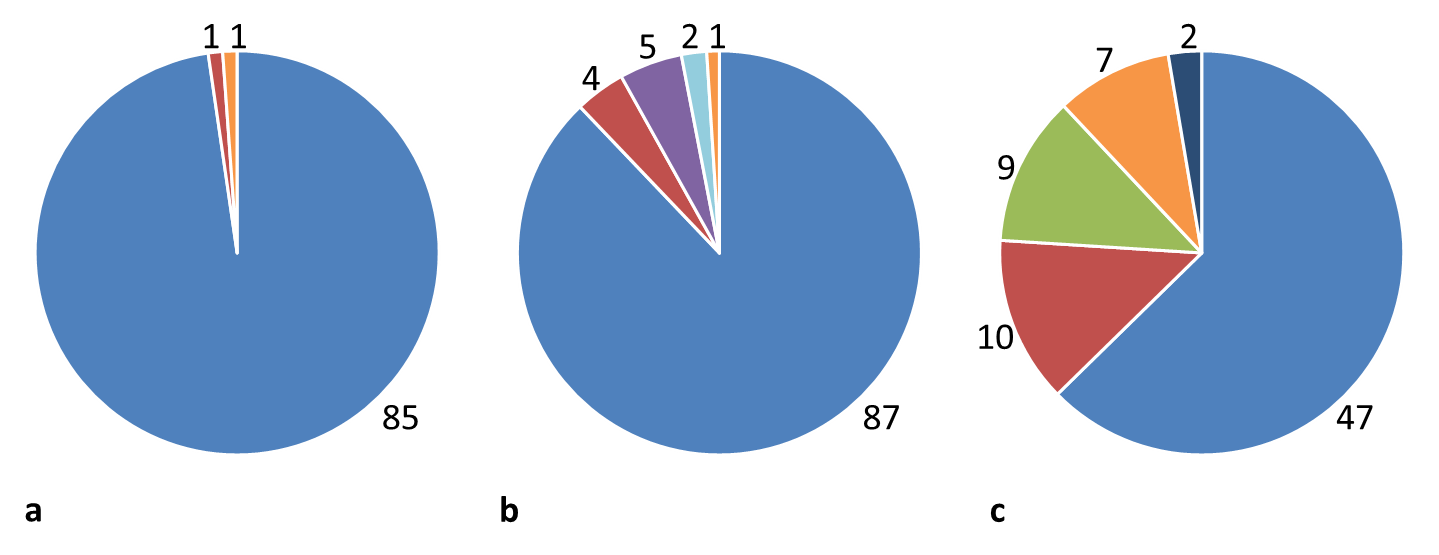
**Figure S65** Inorganic materials identified as the main components in kohl, (a) Previously analysed with modern methods, (b) samples analysed with modern methods including data from this study, (c) Samples analysed pre-1930[^17^](https://paperpile.com/c/9BI6u0/i4YBA). Pb based, Mn based, Cu based, Fe based, organic-based, silicon-based, other.


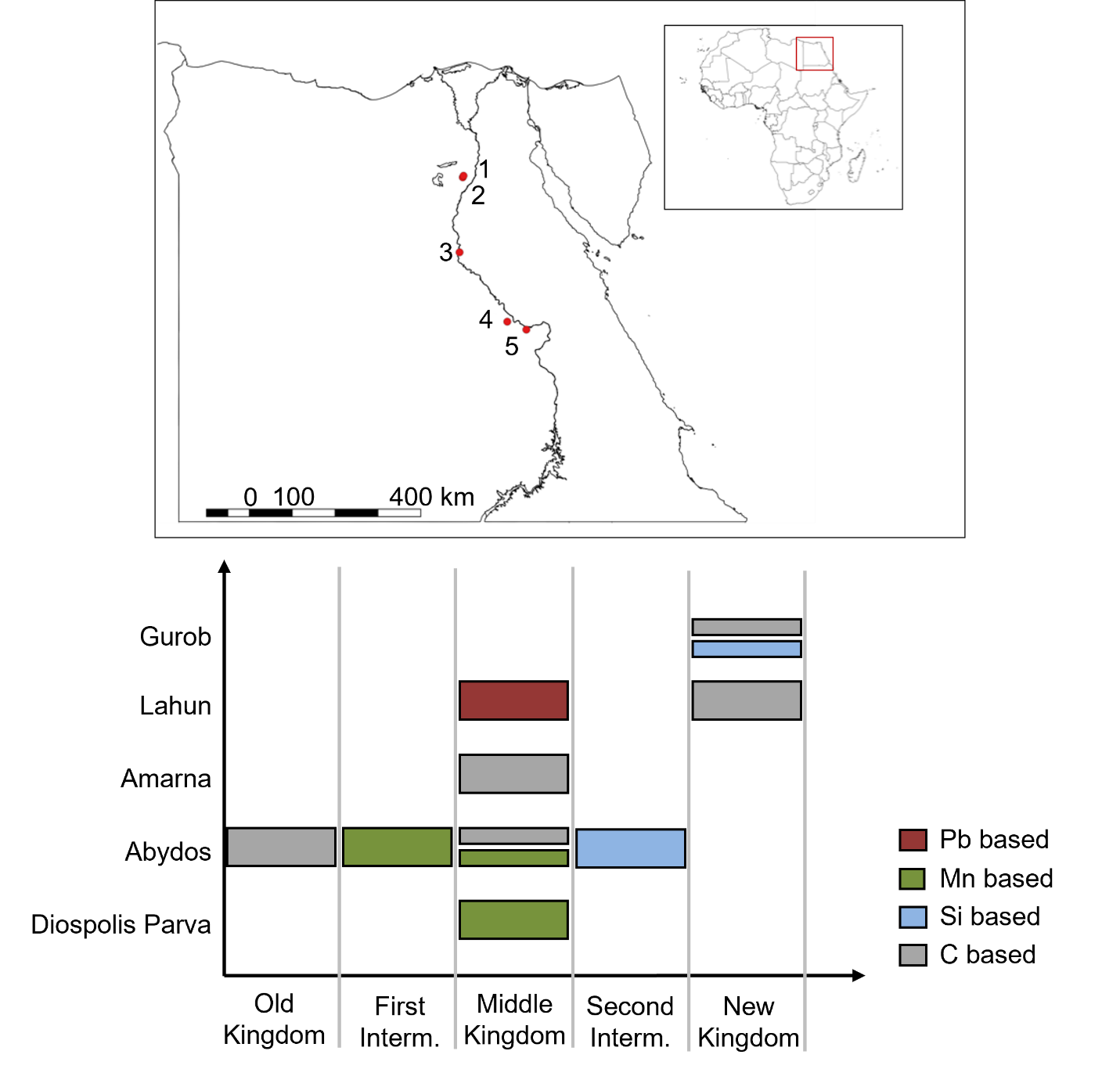

**Figure S66** Graphic highlighting the primary inorganic components by time period and location of the objects sampled from the Petrie Museum collection. One object with unknown provenance (UC64751) is not represented in this figure.

**Figure S67** Time periods of all specimens analysed with modern methods including those analysed in our study.

**Figure S68** Locations of all specimens analysed with modern methods including those analysed in our study. Other (known) only includes locations with three samples or less.

**Appendix 5**

***Materials and Methods***

**Table S15** Accession records extracted from the open-access database<http://petriecat.museums.ucl.ac.uk/> and description of the sampling. “Object provenance” and “Object description” in this table are based on original accession records of the Petrie Museum and reflect the assessments given by F. Petrie at the time the object arrived in the collections. These descriptions are not indicative of results presented in this study.

| **Specimen No.** | **Object Provenance** | **Object description** | **Sample description** |
| --- | --- | --- | --- |
| UC43159 | Abydos, Old Kingdom | calcite cylinder vase, everted rim, chipped and broken all around circumference, tapered to flat base, kohl on interior | black residue observed, sample from inside vessel |
| UC42810 | Abydos, Middle Kingdom | Calcite kohl pot with kohl remaining inside and traces on flat topped rim, rim sloped in to keel above constricted neck, rounded shoulder body tapered to flat base with slightly projecting rim | black residue observed, sample taken on interior of neck in the area of highest concentration |
| UC43078 | Abydos, 1^st^ Intermediate/Middle Kingdom (Dynasty XI) | calcite kohl pot, wide crudely cut with angled shaping still evident, flat topped with circular cut interior, flat base, galena kohl remaining on interior | black residue observed, sample taken on interior of neck of vessel |
| UC43148 | Abydos, Middle Kingdom (Dynasty XII) | calcite kohl pot, flat topped rim, constricted neck, rounded shoulder, body tapered to flat base with extended rim, contents in interior | black residue observed, sample taken from the interior of the neck, opposite the break in the vessel |

**Table S15** Continued.

| **Specimen No.** | **Object Provenance** | **Object description** | **Sample description** |
| --- | --- | --- | --- |
| UC64751 | Unknown, Middle Kingdom | Anhydrite kohl pot, tall body, broad flat rim, narrow flat base; rim and base slightly abraded; modern cotton wool in mouth to keep residue inside; formerly in the collection of Grenfell, then in that of Henry Wellcome | sample taken from the loose powder present inside the vessel after removal of the modern cotton wool by a conservator of the museum |
| UC46348 | Amarna, ‘Middle Kingdom (?)’ | Dark brown stone (mafic) vessel: kohl pot. No rim. Gently sloping shoulder. Flat circular base.The top of neck flares outward to meet flat rim. Ridges on edge of rim. Concentric grooves and adhering matter on interior wall of vessel | Flake of residue taken from inside the vessel. |
| UC7321 | Lahun, Late Middle Kingdom | Black serpentine kohl pot, with lid. Containing powdered kohl. | Loose powder from inside vessel. |
| UC6742a | Lahun, New Kingdom (early Dynasty XVIII) | Horn kohl pot with wooden base, remainder of kohl (galena eyepaint) inside; found inside casket UC 6741. | Residue observed, sample taken from inside the vessel body. |
| UC7890a | Gurob, New Kingdom  (Dynasty XIX) | Wooden kohl-pot; quadruple tubes, one with green powder (Sample B), plug of linen probably belongs, one with black kohl (Sample A), one empty, on cut through the bottom; bronze wire loop for holding stick, holes for another loop above | Black sample from inside the tube next to empty cylinder |
| UC7890b |  |  | Green sample taken from inside quad tube, specimen had a linen textile plug |

**Table S15** Continued.

| **Specimen No.** | **Object Provenance** | **Object description** | **Sample description** |
| --- | --- | --- | --- |
| UC43107 | Abydos, Second Intermediate Period | Small marl ware pottery jar.  Kohl on interior. | Residue observed, sample taken from inside vessel. |
| UC31613 | Diospolis Parva Cemetery, Middle Kingdom (Dynasty XII) | Calcite thin brim kohl pot, with contents. | Residue sample taken from the inside, base of the vessel. |

**Additional References (Supplementary Information)**

71. National Gallery, London, "IMP00499, Calcium oxalate, synthetic, primarily whewellite". in Infrared and Raman Users Group Spectral Database (eds. Price, B. A., Pretzel, B. & Lomax, S. Q.) <http://www.irug.org/jcamp-details?id=1173> (2022).

72. [Panagopoulou, A., Karanasios, K. & Xanthopoulou, G. Ancient Egyptian blue (CaCuSi4O10) pigment by modern solution combustion synthesis method. *Eurasian Chem.-Technol. J.* **18**, 31–37 (2016).](http://paperpile.com/b/29uT4b/NU2o)

73. [Martens, W. & Frost, R. L. An infrared spectroscopic study of the basic copper phosphate minerals: Cornetite, libethenite, and pseudomalachite. *Am. Mineral.* **88**, 37–46 (2003).](http://paperpile.com/b/29uT4b/jCvo)

74. [Abdelaal, S., Mahmoud, N. & Detalle, V. A technical examination and the identification of the wood, pigments, grounds and binder of an ancient Egyptian sarcophagus. *International Journal of Conservation Science* **5**, (2014).](http://paperpile.com/b/29uT4b/YM1I)
